# Introduced *Pheidole* of the world: taxonomy, biology and distribution

**DOI:** 10.3897/zookeys.543.6050

**Published:** 2015-12-09

**Authors:** Eli M. Sarnat, Georg Fischer, Benoit Guénard, Evan P. Economo

**Affiliations:** 1 Department of Entomology, University of Illinois at Urbana-Champaign, 320 Morrill Hall 505 South Goodwin Avenue, Urbana, IL 61801, USA; 2 Okinawa Institute of Science and Technology Graduate University, 1919-1 Tancha, Onna-son, Okinawa, Japan, 904-0495; 3 School of Biological Sciences, The University of Hong Kong, Kadoorie Biological Sciences Building, Pok Fu Lam Road, Hong Kong SAR, China; 4 Department of Ecology and Evolutionary Biology, University of Michigan, 830 N. University St., Ann Arbor, MI 48109 USA

**Keywords:** Applied systematics, big-headed ant, invasive species, invasive ants, identification key, Lucid key, *Pheidole megacephala*, port of entry, species distribution

## Abstract

The objective of this study is to provide a detailed taxonomic resource for identifying and studying ants in the genus *Pheidole* that have established beyond their native ranges. There is an increasing need for systematists to study taxa of specific concern to 21^st^ century environmental, food security and public health challenges. Systematics has an important role to play in both the theoretical and applied disciplines of invasion biology. Few invaders impact terrestrial ecosystems more than ants. Among the world’s 100 worst invasive species is the cosmopolitan and highly destructive *Pheidole megacephala* (Fabricius). Accurate identification of *Pheidole megacephala* is imperative for the success of screening, management and eradication programs designed to protect native ecosystems from the impacts of this destructive species. However, accurate identification of *Pheidole* species is difficult because of their taxonomic diversity, dimorphic worker caste and lack of taxonomic resources. Illustrated keys are included, along with the taxonomic history, taxonomic diagnoses, biological notes and risk statements for the 14 most invasive members of the genus. Global distribution maps based on over 14,000 specimen and literature records are presented for each species. These results of this work will facilitate identification of pest species, determination of climatic and habitat requirements, discovery of pest origins, horizon scanning and assessment of invasion pathways. The following new synonym is proposed, with the senior synonym listed first and the junior synonyms in parentheses: *Pheidole indica* Mayr (= *Pheidole teneriffana* Forel, and its synonyms *Pheidole taina* Aguayo and *Pheidole voeltzkowii* Forel). *Pheidole navigans* Forel, **stat. rev.**, **stat. n.** is removed from synonymy and elevated to species rank. It is proposed that records of *Pheidole moerens* Forel outside of the Mesoamerica and the Caribbean refer instead to *Pheidole navigans* or other heterospecific taxa in the *Pheidole flavens* species complex. We propose that the names *Pheidole anastasii* Emery and *Pheidole floridana* Emery have been widely misapplied to North American outdoor records of *Pheidole bilimeki* Mayr. It is suggested that the synonymy of *Pheidole lauta* Wheeler be transferred from *Pheidole floridana* Emery to *Pheidole bilimeki* Mayr.

## Introduction

The science of systematics has historically focused on the discovery, description and classification of life on earth with relatively little attention given to the ecological or economic impact of the study taxon. Today, there is an increasing need for systematists to study taxa of particular concern to 21^st^ century environmental, food security and public health challenges. Systematics has an important role to play in both the theoretical and applied disciplines of invasion biology (Gotzek et al. 2012; Le Roux and Wieczorek 2009). Although biological invasion is most closely associated with the recent range expansions of species during the Anthropocene, the dispersal of colonist taxa into novel ecosystems is as old as life itself. Study of contemporary invasion ecology and its evolutionary consequences informs a deeper understanding of prehistoric patterns and processes. Correspondingly, study of ecological and evolutionary impacts of ancient colonization events gives historical context to contemporary invasions. Systematics can help bridge this gap separating invasion biology from biodiversity science by advancing integrative theories such as taxon cycles (Economo and Sarnat 2012; Ricklefs and Bermingham 2002). Systematics is also critical to the success of invasive species management. Pest screening, early detection programs and monitoring efficacy all depend on accurate, species-level identifications. Obtaining high-confidence identifications is particularly difficult for hyper-diverse groups such as insects. The few taxonomic resources that exist for insect identification are too often dated, regional, poorly illustrated, and inaccessible to non-specialists.

Invasive species are among the most significant drivers of global change, and few invaders impact terrestrial ecosystems more than ants (Lach and Hooper-Bùi 2009). Of the approximately 15,000 described ants species, more than 100 have established populations outside of their native ranges (McGlynn 1999). Although invasive ants are economically costly in both urban and agricultural areas, the most serious consequences of their introduction may be ecological. Invasive ants can greatly modify ecosystems by reducing native ant diversity, displacing other arthropods, negatively impacting vertebrate populations, and disrupting ant-plant mutualisms (Suarez et al. 2009).

A small subset of introduced ants have become highly destructive invaders, five of which are currently listed among the world’s 100 worst invasive species (Lowe et al. 2000). Unfortunately, detection of non-native ants is hampered by the taxonomic specialization required for accurate species identification of these tiny and overwhelmingly diverse insects. Most of the scientists responsible for identifying ant specimens for pest screening, early-detection programs and monitoring initiatives are not trained ant taxonomists. Although advances in specimen imaging, interactive keys and online resources are welcome developments, increasing the taxonomic capacity for identifying invasive, introduced and commonly intercepted ant species remains a challenge (Sarnat 2011).

Among the world’s 100 worst invasive species (Lowe et al. 2000) is the cosmopolitan and highly destructive *Pheidole megacephala* (Fabricius), known commonly as the big-headed ant. Accurate identification of *Pheidole megacephala* is imperative for the success of screening, management and eradication programs designed to protect native ecosystems from harmful impacts. Many non-specialists – and many myrmecologists – have confused other species of *Pheidole* with *Pheidole megacephala* (Wetterer, 2012). This is not surprising. *Pheidole* (Formicidae: Myrmicinae) is arguably the most speciose monophyletic ant genus in the world, with over 1,000 extant valid species, 138 valid subspecies (Bolton 2014) and hundreds of undescribed species. Accurate identification of *Pheidole* species is especially difficult because of their taxonomic diversity, dimorphic worker caste, and lack of taxonomic resources. The term *‘Pheidole* sp.’ is ubiquitous across ecological and biodiversity publications, including many that focus on tramp ants (e.g. Passera 1994). Recent revisions of *Pheidole* (Eguchi 2001b; 2004b; 2008; Eguchi et al. 2007; Fischer and Fisher 2013; Fischer et al. 2012; Longino 2009; Longino and Cox 2009; Sarnat 2008; Wilson 2003) have advanced the taxonomy of this difficult group. Even at the pace of these past decades, it will be generations before modern identification resources will be available for the majority of known species.

Identification resources for diagnosing the world’s 14 most invasive *Pheidole* species are presented here (Table 1). These resources include a fully illustrated key, specimen photographs and distribution maps, in addition to sections on taxonomic history, taxonomic diagnoses, biology, and risk statements. The results of this work will facilitate identification of pest species, determination of climatic and habitat requirements, discovery of pest origins, horizon scanning, and assessment of invasion pathways.

**Table 1.** Species of *Pheidole* recorded as established outside of their native bioregion. The clade or species group to which each species belongs is listed and defined by the given authority. Clade names are informal designations and are given to convey broad evolutionary relationships among the species. Asterisks (*) note clade designations that are presumed based on morphological similarity.

Species	Clade/Group	Native bioregion	Source
*Pheidole anastasii* Emery	*punctatissima* clade	Neotropics	(Economo et al. 2015, unpublished data; Moreau 2008)
*Pheidole bilimeki* Mayr	*punctatissima* clade	Neotropics	(Economo et al. 2015, unpublished data; Moreau 2008) (as *Pheidole floridana*)
*Pheidole fervens* Smith, F.	*fervens* clade	Indoaustralia	(Economo et al. 2015)
*Pheidole flavens* Roger	*flavens*-complex	Neotropics	(Economo et al. 2015; Moreau 2008)
*Pheidole indica* Mayr	*fervens* clade	Indoaustralia	(Economo et al. 2015, unpublished data)
*Pheidole megacephala* (Fabricius)	*megacephala* group	Afrotropics	(Economo et al. 2015)
*Pheidole navigans* Forel	*flavens*-complex	Neotropics	Unpublished data (see discussion under species account)
*Pheidole noda* Smith, F.	*noda* clade	Indoaustralian	(Economo et al. 2015)
*Pheidole obscurithorax* Naves	*fallax* clade	Neotropics	(Economo et al. 2015; Moreau 2008)
*Pheidole parva* Mayr	*parva* clade	Indoaustralia /Indomalaya	(Economo et al. 2015)
*Pheidole proxima* Mayr	*ampla group	Indoaustralia (Australia)	–
*Pheidole punctatissima* Mayr	*punctatissima* clade	Neotropics	(Economo et al. 2015, unpublished data)
*Pheidole rugosula* Forel	*variabilis clade	Indoaustralia (Australia)	(Economo et al. 2015)
*Pheidole vigilans* (Smith, F.)	undefined	Indoaustralia (Australia)	–

This analysis of the world’s introduced *Pheidole* indicates that two of the most widespread tramp species, *Pheidole indica* Mayr and *Pheidole teneriffana* Forel, each the subject of considerable research and attention, are actually conspecific. This underscores the importance of systematics in understanding biodiversity dynamics in the Anthropocene.

## Defining invasiveness

Biological invasion is a process that is most simply described by successive stages of transport, introduction, establishment and spread (Vermeij 1996). Quantifying invasiveness is a difficult task, but one made easier by the unified framework for biological invasion proposed by Blackburn et al. (2011). We incorporate *Pheidole* taxa into this framework in an effort to advance comparative invasion biology, but acknowledge that these categories fail to convey the idiosyncrasies of each lineage’s invasion history. Separating native range from introduced range is similarly problematic. It is likely that all the treated species are expanding their range within their native biogeographical region as the result of human activity and global environmental change. We therefore define introduced populations strictly as those occurring beyond the lineage’s native biogeographic realm.

Following the Blackburn et al. (2011) framework, we assign four *Pheidole* lineages to category ‘C0’ (individuals released into the wild in location where introduced, but incapable of surviving for a significant period) (Table 2). *Pheidole noda* fits this category, as it is recorded as being introduced only in European greenhouses and is not known to have established permanent populations outside its native range in Asia. The other three species we assigned to the ‘C0’ category all belong to the Mesoamerican *Pheidole punctatissima* clade. All three have been reported as occurring indoors beyond their native bioregion. Of these, *Pheidole bilimeki* is the most widespread and maintains established reproducing outdoor populations in the southern United States. While it can be argued that these northern populations resulted from human-mediated dispersal, they remain contiguous with putatively native Mesoamerican populations, thus we refrain from defining them as introduced.

**Table 2.** Biological characteristics of introduced *Pheidole* species arranged by species name. Sizes (head width measured in mm) are the same as the observed values reported in the species accounts. Invasiveness codes refer to Blackburn et al. (2011). Asterisks indicate name used in reference is considered here to be either a junior synonym or misapplication. References listed: (1) Birkemoe and Aak 2008, (2) Longino and Cox 2009, (3) Naves 1985, (4) Wilson 2003, (5) Morrison 1996, (6) Passera 1994, (7) Reimer 1994, (8) Martínez 1996, (9) Chen et al. 2011, (10) Wilson and Taylor 1967, (11) Boer and Vierbergen 2008, (12) Sarnat and Economo 2012, (13) Longino 2014, (14) Martínez 1992, (15) Fischer and Fisher 2013, (16) Hölldobler and Wilson 1990, (17) Hoffmann 1998, (18) Delabie et al. 1995, (19) Yamamoto et al. 2009, (20) Yamawo et al. 2012, (21) King and Tschinkel 2007, (22) Storz and Tschinkel 2004, (23) Man and Lee 2012, (24) Green and Gunawardana 2006, (25) Berry et al. 1997, (26) Harris et al. 2005, (27) Wilson 1987, (28) Terayama et al., (29) Yamane et al.

Species	Size of major (HW)	Size of minor (HW)	Gyny	Colony structure	Colony foundation	Colony size	Indoor pest	Forages on or nests in vegetation	Invasiveness
*Pheidole anastasii*	0.83–1.05	0.38–0.50	monogynous [1]	polydomous [2]	–	–	yes [1, 2]	yes [2]	C0
*Pheidole bilimeki*	0.75–1.04	0.42–0.52	monogynous [1, 3*, 4*]	polydomous [4*]	dependent [3*]	600–4000 [3*, 4*, 27*]	yes [1, 2]	yes [2]	C0
*Pheidole fervens*	1.13–1.44	0.52–0.63	polygynous [5-7]	polydomous, unicolonial [7, 8]	–	ca. 1000 [9]	yes [10, 11]	yes [12]	D2
*Pheidole flavens*	0.68–0.83	0.34–0.45	–	–	–	2000+[4]	no	yes [13]	D2
*Pheidole indica*	1.32–1.74	0.50–0.65	polygynous [28]	polydomous [14]	dependent [14]	500–1000	no	yes [15]	D2
*Pheidole megacephala*	1.10–1.54	0.50–0.61	polygynous [16]	unicolonial [17]	dependent [17]	10,000+ (?)	yes	yes [11, 18]	E
*Pheidole navigans*	0.84–0.88	0.40–0.45	monogynous	monodomous [3*]	dependent [3*]	600+[3*]	no	–	D2
*Pheidole noda*	1.58–1.82	0.57–0.66	polygynous (?)[19, 28]	–	dependent (?)[19]	3000 [28]	no	yes [20]	D2
*Pheidole obscurithorax*	1.47–1.70	0.60–0.67	monogynous [21]	monodomous [21, 22]	–	~10,000 [21]	no	–	D2
*Pheidole parva*	0.85–0.92	0.39–0.50	–	–	–	–	yes [23]	yes [15]	D2
*Pheidole proxima*	0.95–1.05	0.46	–	monodomous [24]	–	–	yes [25]	–	D2
*Pheidole punctatissima*	0.86–1.06	0.44–0.50	–	–	–	–	yes [2]	yes [2]	C0
*Pheidole rugosula*	0.88	0.45	–	–	–	–	yes [25, 26]	yes [25]	D2
*Pheidole vigilans*	1.30	0.55	–	–	–	–	yes [25]	–	D2

Nine lineages are assigned to category ‘D2’ (individuals surviving in the wild in locations where introduced, with reproduction occurring, and population self-sustaining). The least invasive of these are likely the three Australian species (*Pheidole proxima*, *Pheidole rugosula*, *Pheidole vigilans*) that have established persistent populations in New Zealand, but have not been reported from elsewhere (although *Pheidole vigilans* is reported as introduced in Western Australia). Only *Pheidole rugosula* has been listed in examined interception records, and that was a single New Zealand record from Australia (Ward et al. 2006). *Pheidole parva*, *Pheidole fervens* and *Pheidole indica* are all from the Indomalayan bioregion and have managed to establish reproducing outdoor populations beyond their native range. Although the propagule pressure of these species is relatively strong, as evidenced by their frequent interception at ports of entry (Table 3), the introduced populations of all three tend to be small and relatively localized. *Pheidole obscurithorax* and the *Pheidole flavens*-complex (including *Pheidole flavens* and *Pheidole navigans*) are both Neotropical lineages that have established persistent and actively spreading populations in the southern United States. The former is documented as causing a greater ecological impact, but the latter is more widespread and appears to have greater propagule pressure, as evidenced by high numbers of interception records and establishment of at least temporary populations in California and several Pacific Islands.

**Table 3.** Specimen and literature records of *Pheidole* species intercepted at international ports of entry. The original determinations for specimens included here are available on Antweb.org. [1] Antweb.org (Available from http://www.antweb.org. Accessed 20 March 2015); [2] Boer and Vierbergen 2008; [3] Boer 2015; [4] Ward et al. 2006; [5] Wheeler 1934.

Species	Taxonomic notes	Unique collections	Native bioregion	Bioregion of interception	Record source
*Pheidole bilimeki* Mayr		4	Neotropical	Nearctic	[1]
Pheidole cf. bilimeki		3	Neotropical	Nearctic	[1]
*Pheidole dossena* Wilson		1	Neotropical	Holarctic	[2]
*Pheidole fervens* Smith, F.		235	Indomalaya	Australasia	[4]
*Pheidole fervens* Smith, F.		5	Indomalaya	Nearctic	[1]
*Pheidole fervens* Smith, F.			Indomalaya	Indoaustralia	[5]
*Pheidole fervens* Smith, F.			Indomalaya	Holarctic	[3]
*Pheidole fervida* Smith, F.		2	Indomalaya	Nearctic	[1]
*Pheidole flavens* Roger		2	Neotropical	Nearctic	[1]
*Pheidole flavens*-complex		6	Neotropical	Nearctic	[1]
*Pheidole harrisonfordi* Wilson			Neotropical	Holarctic	[3]
*Pheidole hyatti* Emery			Nearctic	Indoaustralia	[5]
*Pheidole indica* Mayr		1	Indomalaya	Australasia	[4]
*Pheidole indica* Mayr		8	Indomalaya	Nearctic	[1]
*Pheidole indica* Mayr			Indomalaya	Holarctic	[3]
*Pheidole laticornis* Wilson			Neotropical	Holarctic	[3]
*Pheidole megacephala* (Fabricius)		890	Afrotropical	Indoaustralia	[5]
*Pheidole megacephala* (Fabricius)		11	Afrotropical	Nearctic	[1]
*Pheidole noda* Smith, F.		2	Indomalaya	Australasia	[1]
*Pheidole noda* Smith, F.		2	Indomalaya	Nearctic	[1]
*Pheidole noda* Smith, F.			Indomalaya	Indoaustralia	[5]
Pheidole nr. colpigaleata		1	Indomalaya	Nearctic	[1]
Pheidole nr. mantilla		2	Neotropical	Nearctic	[1]
Pheidole nr. marcidula		1	Neotropical	Nearctic	[1]
*Pheidole oceanica* Mayr		< 5	Australasian	Australasia	[4]
*Pheidole pallidula* (Nylander)		2	Holarctic	Nearctic	[1]
*Pheidole pallidula* (Nylander)			Holarctic	Holarctic	[3]
*Pheidole parva* Mayr		1	Indomalaya	Nearctic	[1]
Pheidole cf. parva		1	Indomalaya	Nearctic	[1]
*Pheidole perpusilla* Emery		2	Neotropical	Nearctic	[1]
Pheidole cf. pubiventris		1	Neotropical	Nearctic	[1]
*Pheidole punctatissima* Mayr			Neotropical	Holarctic	[3]
Pheidole cf. punctatissima		12	Neotropical	Nearctic	[1]
Pheidole cf. punctatissima			Neotropical	Indoaustralia	[5]
*Pheidole punctulata* Mayr		1	Afrotropical	Nearctic	[1]
*Pheidole radoszkowskii* Mayr			Neotropical	Holarctic	[3]
*Pheidole rugosula* Forel		1	Australasia	Australasia	[4]
*Pheidole sexspinosa* Mayr		1	Australasian	Australasia	[4]
*Pheidole* sp. mg126	nr. *longispinosa*	1	Afrotropical	Nearctic	[1]
*Pheidole* sp. POE fallax group-a	*fallax* group	1	Neotropical	Nearctic	[1]
*Pheidole* sp. POE pilifera group-a	*pilifera* group	1	Neotropical	Nearctic	[1]
*Pheidole* sp. POE dilligens group-a	*dilligens* group Wilson	1	Neotropical	Nearctic	[1]
*Pheidole* sp. POE-F	*megacephala* group	1	Afrotropical	Nearctic	[1]
*Pheidole* sp. POE-G	*megacephala* group	1	Afrotropical	Nearctic	[1]
*Pheidole* sp. POE-H	*flavens* group Wilson	1	Neotropical	Nearctic	[1]
*Pheidole* sp. POE-I	*flavens* group Wilson	1	Neotropical	Nearctic	[1]
*Pheidole* spec. 1				Holarctic	[3]
*Pheidole* spec. 2				Holarctic	[3]
*Pheidole subarmata* Mayr			Neotropical	Holarctic	[3]
*Pheidole susannae* Forel		2-5	Neotropical	Holarctic	[2]
*Pheidole susannae* Forel		1	Neotropical	Nearctic	[1]
*Pheidole umbonata*		< 5	Indoaustralia	Australasia	[4]

*Pheidole megacephala* is the only species assigned to category ‘E’ (fully invasive species, with individuals dispersing, surviving and reproducing at multiple sites across a greater or lesser spectrum of habitats and extent of occurrence). The vast majority of introduced *Pheidole* specimen and occurrence records are attributed to *Pheidole megacephala*. It is the most geographically widespread species in the entire genus and its impact on native ecosystems and agriculture are extensively documented.

## Characteristics of introduced *Pheidole*

### General characteristics and characters associated with invasion success

All *Pheidole* species treated here have a dimorphic worker caste. Their colonies typically have hundreds to thousands of workers. They are all generalist foragers that feed on some combination of dead arthropods, living arthropods, seeds and human foodstuffs. The aforementioned characteristics are shared by nearly all of their congeners, however, and cannot be considered promoters of invasion success among *Pheidole*. There is a suite of biological characters that are broadly associated with introduced populations of invasive ants, including unicoloniality and omnivory (Holway et al. 2002). Unicoloniality – defined as the ability to form expansive and polygynous (multiple queened) supercolonies – has only been observed in *Pheidole megacephala* (Table 1). Only three other species (*Pheidole fervens*, *Pheidole indica* and *Pheidole noda*) are reported to be at least facultatively polygynous. Four species besides *Pheidole megacephala* are reported to exhibit polydomous populations (*Pheidole anastasii*, *Pheidole bilimeki*, *Pheidole fervens* and *Pheidole indica*).

One interesting pattern deserving further study is the propensity of introduced *Pheidole* to use vegetation for either foraging or nesting (Table 1). *Pheidole* species, in general, are most strongly associated with the ground, and exploitation of the vegetative or arboreal strata is relatively uncommon. One potential reason that foraging and nesting in vegetation is overrepresented among the introduced species is that, if quarantine interception records are any indicator, human-mediated dispersal events are predominately associated with commercial trade of plants or plant material (Suarez et al. 2005; Ward et al. 2006). Furthermore, none of the introduced *Pheidole* species are strictly arboreal, and their capacity for occurring on vegetation reflects the type of broad habitat tolerances required for successful establishment.

## Taxonomic patterns

Although referring to each of the included lineages as a discrete biological species is convenient, there are at least some instances – including the *Pheidole flavens* species complex and *Pheidole megacephala* complex – that defy such neat classification. A disproportionate number of synonyms and infraspecific names in the genus *Pheidole* belong to the lineages treated here, and this pattern holds true across the Formicidae. We offer several explanations for this pattern. The first is attributed to nomenclatural artifact. Taxonomists unfamiliar with distant faunas and working outside of a global context often described introduced populations as new species. The second explanation for the myriad names associated with invasive species reflects a truly biological pattern: *invasive populations tend to be derived from geographically widespread and morphologically variable lineages*. Geographically widespread species have greater propagule pressure because they are broadly exposed to opportunities for human-mediated dispersal (Theoharides and Dukes 2007). The taxonomic work undertaken during this study suggests the phenotypic diversity of many of these introduced lineages is only a thumbnail of a much broader morphological spectrum observed across their respective native ranges.

### Morphological patterns

The 14 *Pheidole* species treated here do not adhere to a particular morphotype, especially when phylogenetic relationship is corrected for. Although none of the species occupy the extreme ends of the genera’s size spectrum, they do range from small to large. None of these species exhibit aberrant or specialized morphology, such as spinescence. (Although not treated in this review, the *Pheidole sexspinosa* complex is a spinescent lineage that is considered a tramp ant around the Pacific and has likely increased its range with the help of human-mediated transport.)

### Phylogenetic patterns

Invasive *Pheidole* species are not evenly dispersed across the phylogeny (Economo et al. 2015). Rather, a few lineages tend to be responsible for spawning successful invaders. In particular, the *punctatissima* clade, *flavens* clade, *fervens* clade have each given rise to multiple introduced species (Table 1). The most parsimonious explanation for this pattern is that at least some promoters of invasion success are plesiomorphic traits inherited from common ancestors. More generally, all of the known clades to which invasive *Pheidole* belong can broadly be considered tramp groups composed of species that exhibit relatively wide geographic ranges, few habitat constraints, and high infraspecific variability.

### Biogeographical patterns

Strong biogeographical patterns among introduced *Pheidole* are difficult to find. One pattern shared by all introduced *Pheidole* is that they invariably occupy low elevation habitat. This is not surprising, as connectivity is much greater between lower elevation sites (e.g. coastlines and shipping ports) than among higher elevation sites (e.g. montane forests). The invasive *Pheidole* invariably come from tropical and subtropical lineages, but this pattern broadly reflects the richness patterns across the entire genus (Economo et al. 2015). The Neotropical, Afrotropical and Indomalayan regions have all produced *Pheidole* lineages that have invaded other bioregions. Australia is nominally home to three invasive *Pheidole* species, but the introduced populations of all three are restricted to the island of New Zealand (in addition to Lord Howe Island in one instance) and are not likely capable of invading another continental system. Although the common recipient of non-native *Pheidole* introductions, and ant introduction in general (McGlynn 1999), Oceania is the only tropical bioregion from which a successful invader has not evolved. *Pheidole sexspinosa* Mayr and possibly *Pheidole oceanica* Mayr are native to Oceania and widely considered tramp species, but thus far there is no evidence that either has ever successfully established outside the Pacific.

## Methods

### Taxon selection

The taxa treated here represent all *Pheidole* species known to have been introduced outside of their native biogeographic region. These taxa span the spectrum from species that have become naturalized across the globe (such as the highly invasive *Pheidole megacephala*) to species known only to have established temporary indoor populations beyond their native region (such as *Pheidole noda*). We do not include species that are repeatedly intercepted by quarantine but are never recorded as establishing non-native populations. The species included here represent the vast majority of published *Pheidole* quarantine interceptions records (Table 3), and have proven the most capable among their congeners of establishing beyond their native range.

In addition to the quarantine intercepts, there are many synanthropic tramp species of *Pheidole* that are likely expanding across their native bioregion with the inadvertent assistance of human exploration and commerce. This is particularly true in Oceania, where species such as *Pheidole oceanica*, *Pheidole umbonata* Mayr and *Pheidole sexspinosa* are widespread across the entire region. However, we were unable to confirm any records of their introduction outside of Oceania. While excluded from our current study, we advise readers to be aware of these and similarly widespread species. Their expansive ranges increase the propagule pressure for anthropogenic dispersal, and their high tolerance for habitat disturbance pre-adapts them for establishing beach-head populations outside their native ranges.

### Occurrence and specimen records

Our biogeographic data are taken from the Global Ant Biodiversity Informatics (GABI) project, a database consolidating literature, museum, and biodiversity database records on ant species distributions (Suppl. material 1). Each literature record for an occurrence outside the putative native range was examined by reviewing the primary reference and evaluating it for veracity and accuracy. Specimen records included in the GABI database were similarly evaluated. Literature records considered to be derivative (e.g. checklists referring to a previously published record) and records from online checklists were excluded unless the primary reference or specimen record was confirmed. A confirmed literature record in the context of this study means only that the valid name or a synonym was verified as appearing in the text, and does not imply that the species identification was accurate. We verified a total of 14,162 occurrence records.

Locality references of literature records were converted to coordinates where possible. First, all variables that described the location of a record were merged into a single string that contained all descriptive information about a location. Unique values were extracted from these strings resulting in a total of 3,803 unique locations. Locations that referred to large areas such countries or states (n=221) were removed from these 3,803 locations. All of the remaining unique locations were then converted to coordinates using the Bing geocoding API (Microsoft 2015). From these unique locations 1,265 were geocoded with a tolerable precision (+/- 10 km) for a global distribution study. These geocoded locations were manually checked for inconsistencies. Of the remaining 2,538 locations the coordinates given in the database were assigned as the correct coordinates when available (n=1,349). This resulted in a total of 968 locations that lacked coordinates. These records contained problematic locality names and were geocoded manually by correcting the locality name and using the GEOLocate web application (Rios and Bart 2010). Of these 968 records 83 were incorrect and could not be geocoded, and 210 did not refer to a point location (country or state).

### Illustrations

Original specimen images taken by the authors were taken using the Auto-Montage software package (Syncroscopy) in combination with a JVC KY-F7U digital camera mounted on a Leica MZ16 dissecting scope, and the software package Helicon Focus in combination with a Leica DFC450 digital camera mounted on a Leica M205C dissecting scope. Vector artwork used to illustrate character states referred to in the taxonomic key and species diagnoses were made in Adobe Illustrator by tracing specimen photographs. All specimen images are available from Antweb.org and can be searched for using the specimen identifier. All vector illustrations are available from the ‘Introduced *Pheidole* taxonomic characters’ media gallery on Antkey.org (http://antkey.org/en/gallery?f[0]=im_field_smg_galleries%3A33508).

### List of abbreviations of museum collections

The abbreviations follow Evenhuis (2009) and are used in the text in place of the full museum collection name. Type material from these collections examined by the authors is noted in the species accounts.

ANIC Australian National Insect Collection (Canberra, Australia)

BMNH The Natural History Museum (London, United Kingdom)

CASC California Academy of Sciences (San Francisco, California, USA)

MCSN Museo Civico di Storia Naturale “Giacomo Doria” (Genoa, Italy)

MCZC Harvard Museum of Comparative Zoology (Cambridge, Massachusetts, USA)

MHNG Natural History Museum of Geneva (Geneva, Switzerland)

MNHN Muséum National d’Histoire Naturelle (Paris, France)

NHMB Naturhistorisches Museum (Basel, Switzerland)

NHMW Naturhistorisches Museum Wien (Vienna, Austria)

USNM United States National Museum of Natural History (Washington D.C., USA)

### Measurements

Measurements reported here include those taken and reported by various researchers. Original measurements taken by the authors were made with a stereo microscope at 40× magnification using a dual-axis stage micrometer wired to digital readouts. Morphometric measurements were recorded in thousandths of millimetres, but are reported here to the nearest hundredth as a range from minimum to maximum across all measured specimens. Specimens for measurements were chosen to reflect potential morphological variation across the full geographic range. The number of specimens from which measurements were taken for a given caste is referred to by *n*. Measurements for Neotropical *Pheidole* include data supplied by John Longino. Measurements for Old World *Pheidole* include values reported in previously published studies (Eguchi 2001a; 2004b; 2008; Eguchi et al. 2007; Fischer and Fisher 2013).

EL Eye Length (mm): Maximum diameter of eye measured in profile view.

HL Head Length (mm): Maximum distance from the midpoint of the anterior clypeal margin to the midpoint of the posterior margin of the head, measured in full-face view; in majors, measured from midpoint of tangent between anteriormost position of clypeus to midpoint of tangent between posteriormost projection of the vertex.

HW Head Width (mm): Measured at widest point of the head, in full-face view behind eye level.

SL Scape Length (mm): Maximum scape length, excluding basal condyle and neck.

CI Cephalic Index: HW / HL × 100.

SI Scape Index: SL / HW × 100.

### Identification keys to introduced species of *Pheidole*

Readers are warned that there are hundreds of native *Pheidole* species that are not treated in the following keys. The keys are most useful for diagnosing *Pheidole* specimens intercepted at quarantine facilities, collected from regions with depauperate native *Pheidole* diversity (such as small islands), and in highly disturbed habitats such as urban areas. Even in urban areas, however, there remains considerable likelihood that native *Pheidole* species occur that are not treated here, and readers are cautioned to use these keys judiciously.

### Lucid3 Key

An interactive and fully illustrated Lucid3 key that includes all *Pheidole* species treated in this study is available from the website Antkey.org (Sarnat and Suarez 2012) at the following URL: http://antkey.org/en/content/key. To use this key for introduced *Pheidole* identification, users are advised to first filter by the genus *Pheidole*, then proceed by using the ‘best’ and ‘next best’ functions. Users are referred to documentation and video tutorials on the webpage for additional instructions and best practices.

**Major workers only**

**Table:** 

1	Postpetiole swollen relative to petiole; either with a posterodorsal and anteroventral bulge (Fig. 1) or with a single dorsal bulge (Fig. 2)	**2**
–	Postpetiole not swollen relative to petiole (Fig. 3)	**3**
2	Postpetiole with a posterodorsal (Fig. 1a) and anteroventral (Fig. 1b) bulge. Promesonotum in profile forming a single dome (Fig. 4), lacking a distinct mound or prominence on the posterior slope. Head heart-shaped (Fig. 6); dorsal surface smooth, glossy and entirely lacking strong rugoreticulate sculpture	***megacephala***
–	Postpetiole forming a high dorsally bulging dome that is tallest at midpoint (Fig. 2a); ventral margin flat to very weakly convex (Fig. 2b). Promesonotum in profile with two convexities (Fig. 5), the large anterior dome in addition to a distinct prominence on the posterior slope. Head subquadrate (Fig. 7); dorsal surface covered in strong longitudinal rugae that form a reticulated network laterally and posteriorly (Fig. 8)	***noda***
3	Promesonotum in profile with two convexities (Fig. 5), the large anterior dome in addition to a distinct mound or prominence on the posterior slope. Relatively large species with long limbs (HW major > 1.10 mm, HW minor > 0.50 mm). Head with strong rugoreticulate sculpture at least on posterolateral lobes (Fig. 8)	**4**
–	Promesonotum in profile forming a single dome (Fig. 4), lacking a distinct mound or prominence on the posterior slope (sometimes with a weak protuberance or inconspicuous mound). Size and relative limb length variable. Posterolateral lobes variably sculptured including glossy (Fig. 9), rugose (Fig. 10) and punctate (Fig. 11); if strongly rugoreticulate on posterolateral lobes then small species (HW < 1.00 mm)	**6**
4	Head almost entirely covered by network of intersecting rugae (Fig. 12a), lacking long, well-organized and parallel longitudinal rugae on the frons (Fig. 12b). Frontal carinae indistinct, quickly becoming integrated into dense rugoreticulum that covers the entire face. Antennal scrobes entirely lacking. Antennal insertions surrounded by deeply excavated pits (Fig. 12c). Head sometimes a lighter reddish brown than the mesosoma	***obscurithorax***
–	Head rugoreticulate on posterolateral lobes and laterad of frontal carinae (Fig. 13a), but frons dominated by long, well-organized and parallel longitudinal rugae (Fig. 13b). Antennal scrobes indistinct to moderately impressed, but frontal carinae always forming a border capable of accepting the antennal scape (Fig. 13c). Antennal insertions not surrounded by deeply excavated pits. Head usually a similar shade as the mesosoma	**5**
5	Frontal carinae relatively longer, extend 4/5 distance of head before terminating (Fig. 14). Promesonotal prominence flatter, less pronounced (Fig. 63a). Propodeal spine weaker, narrower at base, weakly downcurved at apex (Fig. 63b)	***fervens***
–	Frontal carinae relatively shorter, extend 3/4 distance of head before terminating (Fig. 15). Promesonotal prominence rounder, more pronounced (Fig. 64a). Propodeal spine stouter, broader at base, relatively straight (Fig. 64b)	***indica***
6	Posterolateral lobes lacking sculpture (including foveolate ground sculpture, carinae and rugae) posterior to maximum extent of antennal scapes in repose (Fig. 9). Head glossy, lacking foveolate ground sculpture. Promesonotal dorsum glossy, lacking foveolate ground sculpture or striae (Fig. 23)	**7**
–	Posterolateral lobes with foveolate ground sculpture (Fig. 11), carinae (Fig. 12) or rugae (Fig. 12) distinctly present posterior to maximum extent of antennal scape (if absent then remainder of face is strongly foveolate). Promesonotal dorsum with foveolate ground sculpture, striae or both	**8**
7	Petiolar node strongly punctate (Fig. 16). Metapleuron with moderate rugulae and some weak punctation (Fig. 16). Hypostomal bridge with a small median tooth in addition to a pair of larger inner teeth (Fig. 18)	***proxima***
–	Petiolar node mostly glossy (Fig. 17), not covered by punctate sculpture. Metapleuron almost completely glossy with strongly reduced carinulae and lacking punctation (Fig. 17). Hypostomal bridge with two well-developed inner teeth but lacking a median tooth (Fig. 19)	***vigilans***
8	Promesonotal dorsum glossy with thin but distinct subparallel striae running oblique to the longitudinal midline (Fig. 20). Head with distinct parallel rugae extending from frontal lobes posterior to apices of frontal carinae. Shorter lengths of rugae present across entire posterior region of head and extending to posterior margin in full-face view (Fig. 24).	***rugosula***
–	Promesonotal dorsum with various sculpture patterns including transversely striate (Fig. 21), longitudinally striate to rugoreticulate (Fig. 22), and lacking striae (Fig. 23); but never with subparallel striae running oblique to the longitudinal midline. Head variously sculptured, but if sculpture reaches posterior head margin in full-face view it is either strongly rugoreticulate (Fig. 26) or foveolate (Fig. 11)	**9**
9	Posterolateral lobes, including posterior head margin, strongly rugoreticulate (Fig. 26). Promesonotum in dorsal view strongly transverse with strongly projecting shoulders (Fig. 28). Promesonotal dorsum rugoreticulate with distinct long longitudinal striae in addition to shorter sections of transverse and intersecting striae (Fig. 22)	***parva***
–	Posterolateral lobes variously sculptured, but posterior head margin always free of distinct rugae (Fig. 25) or rugoreticulum (Fig. 27). Promesonotum in dorsal view less transverse with weakly projecting shoulders in dorsal view (Fig. 29). Promesonotal dorsum variously sculptured (including transversely striate (Fig. 21), foveolate or both), but never rugoreticulate with distinct long longitudinal striae	**10**
10	Gaster with entire first tergite glossy (Fig. 32). Postpetiole relatively narrow (Fig. 30); distinctly less than 2× petiolar width in dorsal view. Promesonotal dorsum usually with distinct transverse striae (Fig. 21), but sometimes lacking distinct striae. Posterolateral lobes variably sculptured. (*Pheidole flavens*-complex)	**11**
–	Gaster with at least anterior 1/3 of first tergite matte (Fig. 33). Postpetiole relatively broad; distinctly more than 2× petiolar width in dorsal view (Fig. 31). Promesonotal dorsum usually foveolate and never with distinct transverse striae. Head often entirely foveolate (Fig. 11), but portions of posterolateral lobes can be glossy. Posterolateral lobes never with distinct rugae	**12**
11	Antennal scrobe distinct, narrow and shallow, but capable of receiving the entire antennal scape in repose (Fig. 71a); bordered by strong, unbroken frontal carina mesially (Fig. 71b); depression marked by a continuous smooth surface entirely (or nearly entirely) uninterrupted by rugulae. The rugulae of the frons extend to approximately an eye’s length distance from the posterior head margin. Promesonotal dorsum with distinct transverse striae (Fig. 21)	***navigans***
–	Antennal scrobe broad, ill-defined, incapable of receiving the entire antennal scape in repose (Fig. 72a); bordered by relatively weak and interrupted frontal carina mesially (Fig. 72b); depression opaque and strongly punctate. The rugulae of the frons of variable length but never reach posterior head margin. Promesonotal dorsum variable, but if transverse striae are present they rarely reach across entire surface	***flavens***
12	Head bicolored, the yellowish posterior two-thirds contrasting with the darker brown anterior third and rest of body (Fig. 34)	***punctatissima***
–	Head uniform in color (Fig. 35), from yellow to reddish brown; same color as associated minor workers	**13**
13	Color usually yellow. Head width sometimes wider (HW 0.74–1.16 mm). Prefers understory habitat. Typically nests arboreally in live plant cavities, under bark, and in dead sticks and branches on or above forest floor	***anastasii***
–	Color usually red brown. Head width sometimes narrower (HW 0.71–1.07 mm). Prefers open, disturbed habitat. Generalist nest microhabitats, including under stones and dead wood.	***bilimeki***

**Minor workers only**

**Table:** 

1	Head predominantly glossy (Fig. 36), lacking punctation and or rugae above eye level	**2**
–	Head conspicuously punctate (Fig. 37) and/or rugose (Fig. 38) above eye level	**8**
2	Postpetiole swollen relative to petiole; either with a posterodorsal and anteroventral bulge (Fig. 1) or with a single dorsal bulge (Fig. 2)	**3**
–	Postpetiole not swollen relative to petiole (Fig. 3)	**4**
3	Postpetiole with a posterodorsal (Fig. 1a) and anteroventral (Fig. 1b) bulge. Antennal scapes surpass posterior head margin by approximately same length as eye (Fig. 40). Promesonotum in profile forming a single dome, lacking a distinct mound or prominence on the posterior slope (Fig. 42)	***megacephala***
–	Postpetiole forming a high dorsally bulging dome that is tallest at midpoint; ventral margin flat to very weakly convex (Fig. 2). Antennal scapes surpass posterior head margin by approximately twice the eye length (Fig. 39). Promesonotum in profile with two convexities, the large anterior dome (Fig. 44a) in addition to a distinct prominence on the posterior slope (Fig. 44b)	***noda***
4	Promesonotum in profile with two convexities, the large anterior dome (Fig. 43a) in addition to a distinct prominence on the posterior slope (Fig. 43b). Antennal scapes relatively long, surpassing posterior head margin by a distance equal (Fig. 40) to or greater than (Fig. 39) eye length. Posterior head margin strongly convex (Fig. 44) to weakly convex (Fig. 45) in full-face view. Color variable	**5**
–	Promesonotum in profile forming a single dome (Fig. 42), lacking a distinct mound or prominence on the posterior slope. Antennal scapes relatively short (Fig. 41), either failing to surpass posterior head margin, or surpassing it by less than the distance of eye length. Posterior head margin weakly convex (Fig. 45) to weakly concave (Fig. 46) in full-face view. Color yellow to brown	**7**
5	Posterior margin strongly convex in full-face view such that the head outline forms a single unbroken curve from eye to eye (Fig. 44). Petiole and postpetiole strongly sculptured laterally (Fig. 47). Antennal scapes extremely long, surpassing posterior head margin by more than 2× eye length (Fig. 39)	***obscurithorax***
–	Posterior head margin weakly convex to flat in full-face view (Fig. 45). Petiole and postpetiole glossy to very weakly sculptured laterally (Fig. 48). Antennal scapes long, but not surpassing the posterior head margin by more than 2× eye length	**6**
6	Promesonotal prominence more flat (Fig. 49a). Metanotal depression deeper (Fig. 49b). Eye relatively small, eye length distinctly less than length of antennal segment 10 (Fig. 65)	***fervens***
–	Promesonotal prominence more convex (Fig. 50a). Metanotal depression shallower (Fig. 50b). Eye relatively large, eye length subequal to length of antennal segment 10 (Fig. 66)	***indica***
7	Antennal scapes surpass posterior head margin by approximate distance of eye length (Fig. 40). Mesopleuron entirely glossy (Fig. 51a). Propodeal spines weakly produced and dentiform (Fig. 51b). Petiole almost entirely glossy	***vigilans***
–	Antennal scapes reach but do not surpass posterior head margin (Fig. 41). Mesopleuron entirely punctate (Fig. 52a). Propodeal spines moderately produced and spiniform (Fig. 52b). Petiole distinctly sculptured except for apical portion of node	***proxima***
8	Head with well-defined, long segments of rugae running longitudinally from below the eyes to the posterior head margin (Fig. 38). Frontal carinae distinct and reaching towards the posterior head margin, although they may occasionally be interrupted (Fig. 38). Punctate ground sculpture present on lateral surfaces of head and just mesad of the frontal carinae, but median portion of head with a large glossy section (Fig. 38). (Native to Australia)	***rugosula***
–	Head, including the area mesad of the frontal carinae, entirely covered by reticulated network of punctures, giving it a dull appearance (Fig. 37); if rugae are present they are generally short segments and mostly restricted to posterior portion of head. Frontal carinae not distinct posterior to eye level	**9**
9	Gaster with at least anterior 1/3 of first tergite matte (Fig. 33). Hairs on mesosoma stout, stiff, of equal length and arranged in pairs (Fig. 53). Antennal scapes lack standing hairs (Fig. 55); scapes surpass posterior head margin by a distance equal to or greater than eye (Fig. 40)	**10**
–	Gaster with entire first tergite glossy (Fig. 32). Hairs on mesosoma fine, flexuous, of unequal length and not arranged in pairs (Fig. 54). Antennal scapes with erect to suberect hairs (Fig. 56); scapes reach posterior head margin but do not surpass it by a distance equal to or greater than eye length (Fig. 41)	**12**
10	Posterior head margin more broad (Fig. 57). Antennal scapes relatively short (SI 95–108). Color usually brown but occasionally yellow	***bilimeki***
–	Posterior head margin more narrow (Fig. 58). Antennal scapes relatively longer (SI 103–125). Color variable	**11**
11	Color usually clear yellow orange (gray brown in one population on Caribbean coast of Panama). Typically nesting in live plant cavities in wet forest understory	***anastasii***
–	Color red brown to nearly black. Typically nesting in open, disturbed habitats	***punctatissima***
12	Posterior portion of head with many short to medium length segments of striae distinctly interlaced among punctate ground sculpture (Fig. 59). Antennal scapes do not surpass posterior head margin (Fig. 41)	***parva***
–	Posterior portion of head lacking many short to medium length segments of striae distinctly interlaced among punctate ground sculpture (Fig. 60). Antennal scapes often, but not always, surpass posterior head margin; if they do it is usually by a distance less than eye length	***flavens*** complex

**Combined major and minor workers**

**Table:** 

1	**Major + minor** Postpetiole swollen relative to petiole (Fig. 1, Fig. 2)	**2**
–	**Major + minor** Postpetiole not swollen relative to petiole (Fig. 3)	**3**
2	**Major + minor** Postpetiole with a posterodorsal (Fig. 1a) and anteroventral (Fig. 1b) bulge. Promesonotum in profile forming a single dome (Fig. 4, major; Fig. 42 minor), lacking a distinct mound or prominence on the posterior slope. **Major** Head heart-shaped (Fig. 6); posterodorsal surface smooth, glossy and entirely lacking strong rugoreticulate sculpture (Fig. 9). **Minor** Antennal scapes surpass posterior head margin by approximately same length as eye (Fig. 40)	***megacephala***
–	**Major + minor** Postpetiole forming a high dorsally bulging dome that is tallest at midpoint (Fig. 2a); ventral margin flat to very weakly convex (Fig. 2b). Promesonotum in profile with two convexities, the large anterior dome in addition to a distinct mound or prominence on the posterior slope (Fig. 5, major; Fig. 43, minor). **Major** Head subquadrate (Fig. 7); dorsal surface covered in strong longitudinal rugae that form a reticulated network laterally and posteriorly (Fig. 8). **Minor** Antennal scapes surpass posterior head margin by approximately twice eye length (Fig. 39)	***noda***
3	**Major + minor** Promesonotum in profile with two convexities, the large anterior dome in addition to a distinct mound or prominence on the posterior slope (Fig. 5, major; Fig. 43, minor). Relatively large species with long limbs (HW major > 1.10 mm, HW minor > 0.50 mm). **Major** Head with strong rugoreticulate sculpture at least on posterolateral lobes (Fig. 8). **Minor** Head glossy (Fig. 36); sculpture restricted to at most a few arcuate carinae between eye and antennal insertion. Antennal scapes with erect hairs (Fig. 56); scapes surpass posterior head margin by at least a distance equal to or greater than eye length (Fig. 39)	**4**
–	**Major + minor** Promesonotum in profile forming a single dome (Fig. 4), lacking a distinct mound or prominence on the posterior slope (sometimes with a weak protuberance or inconspicuous mound). Size and relative limb length variable. **Major** Head with variable sculpture patterns including glossy (Fig. 36), punctate (Fig. 37) and rugose (Fig. 38); if strongly rugoreticulate on posterolateral lobes then small species (HW < 1.00 mm). **Minor** Head variable in sculpture. Antennal scapes with (Fig. 56) or without (Fig. 55) erect hairs; scapes never surpassing posterior head margin by a distance equal to or greater than eye length	**6**
4	**Major** Head almost entirely covered by network of intersecting rugae (Fig. 12a), lacking long, well-organized and parallel longitudinal rugae on the frons (Fig. 12b). Frontal carinae indistinct, quickly becoming integrated into dense rugoreticulum that covers the entire face. Antennal scrobes entirely lacking. Antennal insertions surrounded by deeply excavated pits (Fig. 12c). Head often a lighter reddish brown than the mesosoma. **Minor** Posterior head margin strongly convex in full-face view such that the head outline forms a single unbroken curve from eye to eye (Fig. 44). Petiole and postpetiole strongly sculptured laterally (Fig. 47). Antennal scapes extremely long, surpassing posterior head margin by more than 2× eye length (37)	***obscurithorax***
–	**Major** Head rugoreticulate on posterolateral lobes and laterad of frontal carinae (Fig. 13a), but frons dominated by long, well-organized and parallel longitudinal rugae (Fig. 13b). Antennal scrobes indistinct to moderately impressed, but frontal carinae always forming a border capable of accepting the antennal scape (Fig. 13c). Antennal insertions not surrounded by deeply excavated pits. Head usually a similar shade as the mesosoma. **Minor** Posterior head margin weakly convex to flat in full-face view (Fig. 45). Petiole and postpetiole glossy to very weakly sculptured laterally (Fig. 48). Antennal scapes long, but not surpassing the posterior head margin by more than 2× eye length	**5**
5	**Major** Frontal carinae relatively longer, extend 4/5 distance of head before terminating (Fig. 14). Promesonotal prominence flatter, less pronounced (Fig. 63a). Propodeal spine weaker, narrower at base, weakly downcurved at apex (Fig. 63b). **Minor** Promesonotal prominence more flat (Fig. 49a). Metanotal depression deeper (Fig. 49b). Eye relatively small, eye length distinctly less than length of antennal segment 10 (Fig. 65)	***fervens***
–	**Major** Frontal carinae relatively shorter, extend 3/4 distance of head before terminating (Fig. 15). Promesonotal prominence rounder, more pronounced (Fig. 64a). Propodeal spine stouter, broader at base, relatively straight (Fig. 64b). **Minor** Promesonotal prominence more convex (Fig. 50a). Metanotal depression shallower (Fig. 50b). Eye relatively large, eye length subequal to length of antennal segment 10 (Fig. 66)	***indica***
6	**Major** Posterolateral lobes lacking sculpture (including foveolate ground sculpture, carinae and rugae) posterior to maximum extent of antennal scapes in repose. Head glossy, lacking foveolate ground sculpture. Promesonotal dorsum glossy, lacking foveolate ground sculpture or striae (Fig. 23). **Minor** Head predominantly glossy, lacking punctation and or rugae above eye level. Promesonotal dorsum also glossy without punctate ground sculpture or striae	**7**
–	**Major** Posterolateral lobes with foveolate ground sculpture (Fig. 11), carinae or rugae (Fig. 12) distinctly present posterior to maximum extent of antennal scape (if absent then remainder of face is strongly foveolate). Promesonotal dorsum with foveolate ground sculpture, striae or both. **Minor** Head above eye level with punctate ground sculpture (Fig. 37), rugae (Fig. 38) or both. Promesonotal dorsum with foveolate ground sculpture, distinct striae or both but never glossy	**8**
7	**Major** Petiolar node strongly punctate (Fig. 16). Metapleuron with moderate rugulae and some weak punctation (Fig. 16). Hypostomal bridge with a small median tooth in addition to a pair of larger inner teeth (Fig. 18). Smaller (HW < 1.0 mm). **Minor** Antennal scapes reach but do not surpass posterior head margin (Fig. 41). Mesopleuron entirely punctate (Fig. 52a). Propodeal spines moderately produced and spiniform (Fig. 52b). Petiole distinctly sculptured except for apical portion of node. Smaller (HW < 0.48 mm)	***proxima***
–	**Major** Petiolar node mostly glossy (Fig. 17), not covered by punctate sculpture. Metapleuron almost completely glossy with strongly reduced carinulae and lacking punctation (Fig. 17). Hypostomal bridge with two well-developed inner teeth but lacking a median tooth (Fig. 19). Larger (HW > 1.2 mm). **Minor** Antennal scapes surpass posterior head margin by approximate distance of eye length (Fig. 40). Mesopleuron entirely glossy (Fig. 51a). Propodeal spines weakly produced and dentiform (Fig. 51b). Petiole almost entirely glossy. Larger (HW > 0.52 mm)	***vigilans***
8	**Major** Promesonotal dorsum glossy with thin but distinct subparallel striae running oblique to the longitudinal midline (Fig. 20). Head with distinct parallel rugae extending from frontal lobes posterior to apices of frontal carinae. Shorter lengths of rugae present across entire posterior region of head and extending to posterior margin in full-face view (Fig. 24). **Minor** Head with well-defined, long segments of rugae running longitudinally from below the eyes to the posterior head margin (Fig. 38). Frontal carinae distinct and reaching towards the posterior head margin, although they may occasionally be interrupted (Fig. 38). Punctate ground sculpture present on lateral surfaces of head and just mesad of the frontal carinae, but median portion of head with a large glossy section (Fig. 38)	***rugosula***
–	**Major** Promesonotal dorsum with various sculpture patterns including transversely striate (Fig. 21), longitudinally striate to rugoreticulate (Fig. 22), and lacking striae (Fig. 23); but never with subparallel striae running oblique to the longitudinal midline. Head variously sculptured, but if sculpture reaches posterior head margin in full-face view it is either strongly rugoreticulate (Fig. 26) or foveolate (Fig. 11). **Minor** Head, including the area mesad of the frontal carinae, entirely covered by reticulated network of punctures, giving it a dull appearance (Fig. 37); if rugae are present they are generally short segments and mostly restricted to posterior portion of head. Frontal carinae not distinct posterior to eye level	**9**
9	**Major** Posterolateral lobes, including posterior head margin, covered in rugoreticulum (Fig. 26). Promesonotum in dorsal view transverse with strongly projecting shoulders (Fig. 28). Promesonotal dorsum rugoreticulate with distinct long longitudinal striae in addition to shorter sections of transverse and intersecting striae (Fig. 22). **Minor** Posterior portion of head with many short to medium length segments of striae distinctly interlaced among punctate ground sculpture (Fig. 59). Antennal scapes with many erect hairs (Fig. 56); scapes do not surpass posterior head margin (Fig. 41)	***parva***
–	**Major** Posterolateral lobes variously sculptured, but posterior head margin always free of distinct rugae (Fig. 25) or rugoreticulum (Fig. 27). Promesonotum in dorsal less transverse with weakly projecting shoulders in dorsal view (Fig. 29). Promesonotal dorsum variously sculptured (including transversely striate (Fig. 21), foveolate or both), but never rugoreticulate with distinct long longitudinal striae. **Minor** Posterior portion of head lacking many short to medium length segments of striae distinctly interlaced among punctate ground sculpture (Fig. 60). Antennal scapes with (Fig. 56) or without (Fig. 55) many erect hairs. Scapes often, but not always, surpass posterior head margin; if they do it is usually by a distance less than eye length	**10**
10	**Major + minor** Gaster with entire first tergite glossy (Fig. 32). **Major** Postpetiole relatively narrow; distinctly less than 2× petiolar width in dorsal view (Fig. 30). Posterolateral lobes variably sculptured. **Minor** Hairs on mesosoma fine, flexuous, of unequal length and not arranged in pairs (Fig. 54). Antennal scapes with many erect to suberect hairs (Fig. 56), especially on the anterior margin. Postpetiole narrow in dorsal view, only slightly broader than petiole (Fig. 61). (*Pheidole flavens*-complex)	**11**
–	**Major + minor** Gaster with at least anterior 1/3 of first tergite matte (Fig. 33). **Major** Postpetiole relatively broad; distinctly more than 2× petiolar width in dorsal view (Fig. 31). Promesonotal dorsum usually foveolate and never with distinct transverse striae. Head often entirely foveolate (Fig. 11), but portions of posterolateral lobes can be glossy. Posterolateral lobes never with distinct rugae. **Minor** Hairs on mesosoma stout, stiff, of equal length and arranged in pairs (Fig. 53). Antennal scapes lack many erect to suberect hairs (Fig. 55). Postpetiole broad in dorsal view, distinctly broader than petiole (Fig. 62)	**12**
11	Antennal scrobe distinct and narrow, shallow but capable of receiving the entire antennal scape in repose (Fig. 71a); bordered by strong, unbroken frontal carina mesially (Fig. 71b); depression marked by a continuous smooth surface entirely (or nearly entirely) uninterrupted by rugulae. The rugulae of the frons extend to approximately an eye’s length distance from the posterior head margin. Promesonotal dorsum with distinct transverse striae (Fig. 21)	***navigans***
–	Antennal scrobe broad, ill-defined, incapable of receiving the entire antennal scape in repose (Fig. 72a); bordered by relatively weak and interrupted frontal carina mesially (Fig. 72b); depression opaque and strongly punctate. The rugulae of the frons of variable length but never reach posterior head margin. Promesonotal dorsum variable, but if transverse striae are present they rarely reach across entire surface	***flavens***
12	**Major** Head bicolored with the yellowish posterior two-thirds contrasting with the darker brown anterior third and rest of body (Fig. 33). **Minor** Posterior head margin relatively narrow (Fig. 58). Antennal scapes relatively long (SI 103–125). Color red brown to nearly black	***punctatissima***
–	**Major** Head uniform in color, from yellow to reddish brown; same color as associated minor workers (Fig. 35). **Minor** Posterior head margin relatively narrow or broad. Antennal scapes variable length. Color brown or yellow	**13**
13	**Major + minor** Prefers understory habitat. Typically nests arboreally in live plant cavities, under bark, and in dead sticks and branches on or above forest floor. **Major** Color usually yellow. Head width sometimes wider (HW 0.74–1.16 mm). **Minor** Posterior head margin more narrow (Fig. 58). Antennal scapes relatively longer (SI 103–125). Color brown or yellow	***anastasii***
–	**Major + minor** Prefers open, disturbed habitat. Generalist nest microhabitats, including under stones and dead wood. **Major** Color usually red brown. Head width sometimes narrower (HW 0.71–1.07 mm). **Minor** Posterior head margin more broad (Fig. 57). Antennal scapes relatively short (SI 95–108). Color usually brown but occasionally yellow	***bilimeki***

**Figures 1–19.**
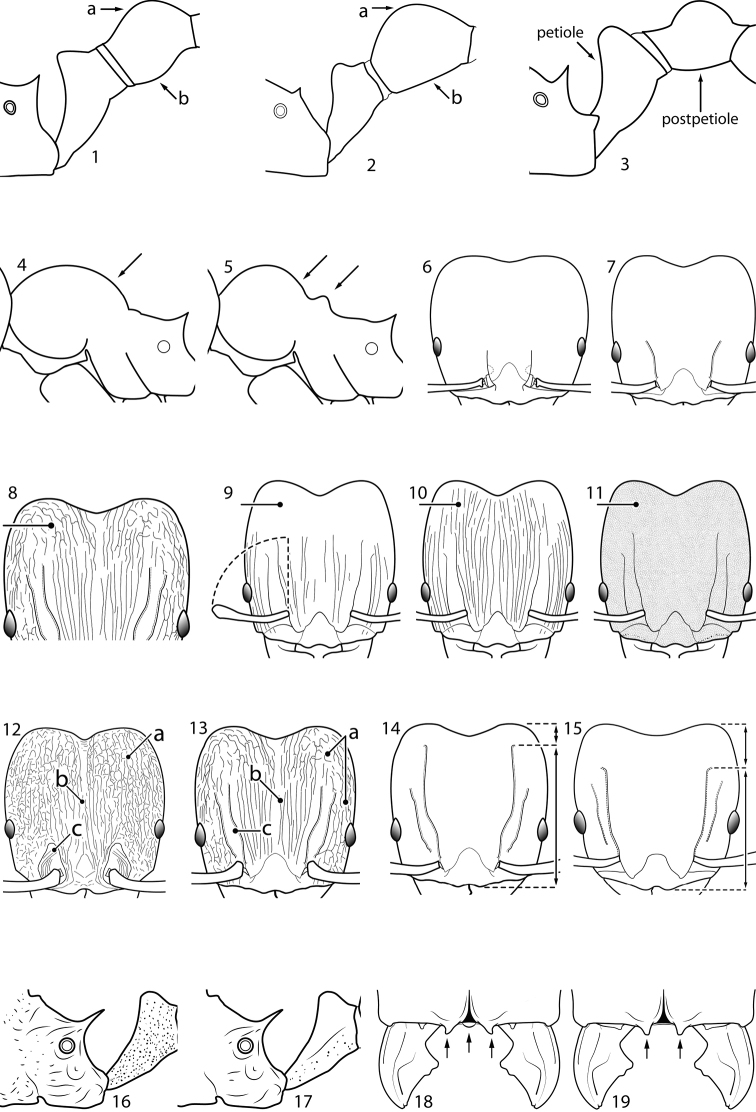


**Figures 20–38.**
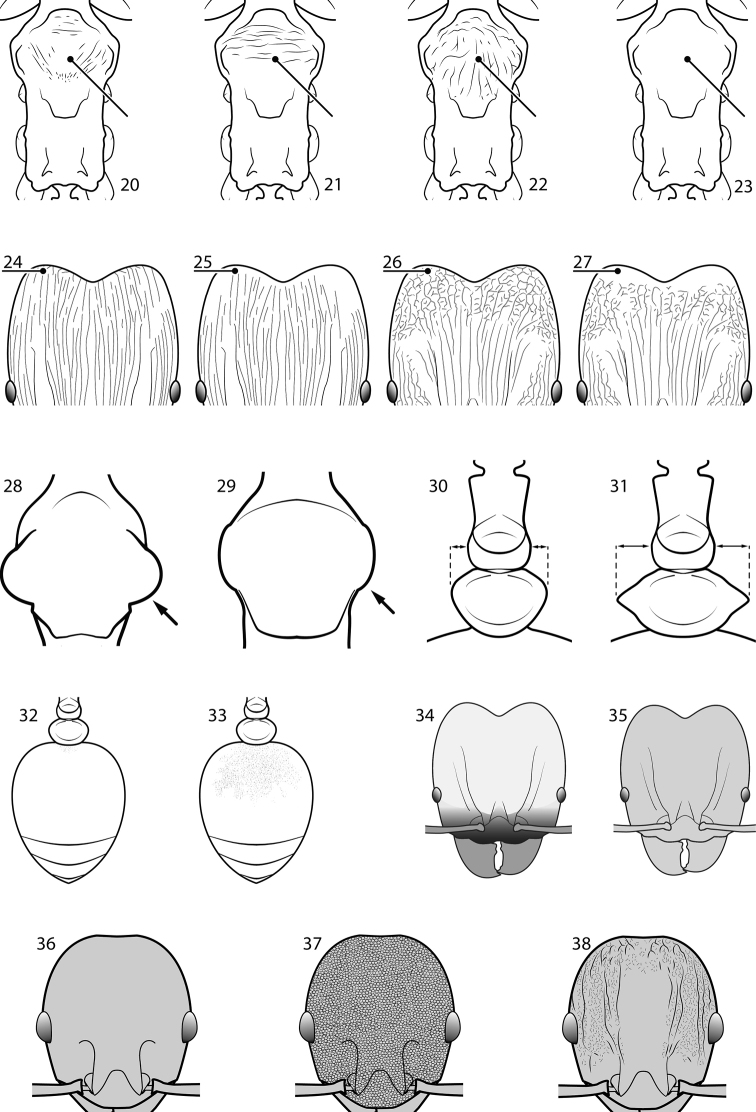


**Figures 39–56.**
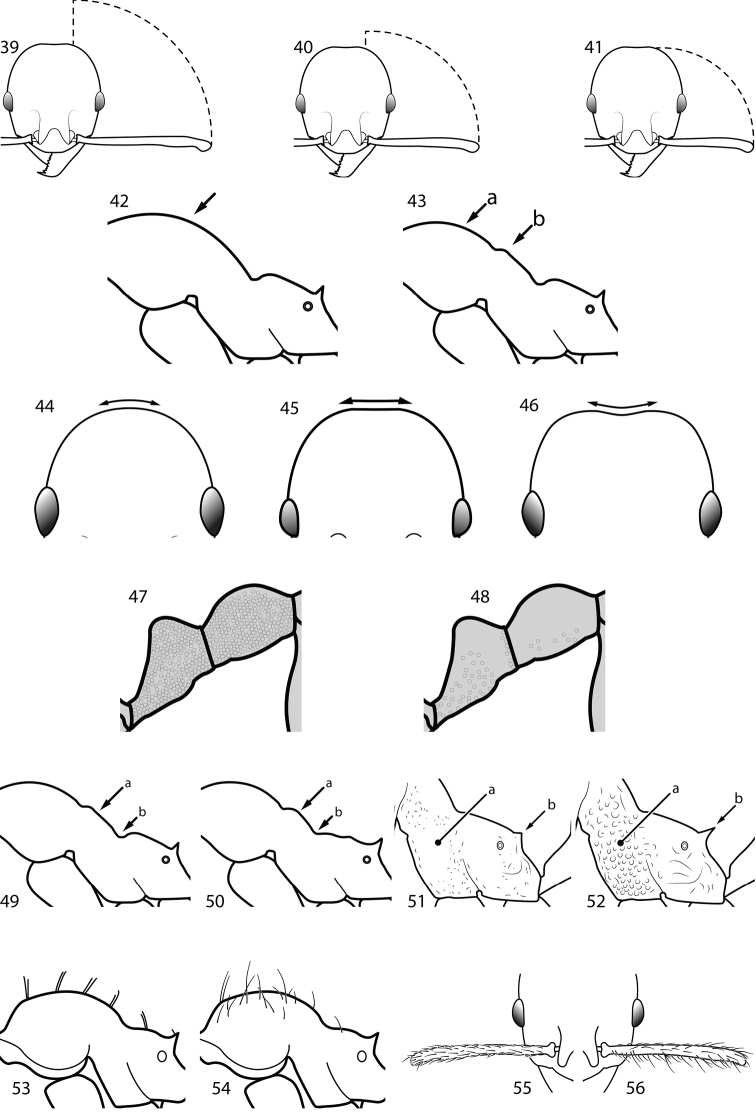


**Figures 57–72.**
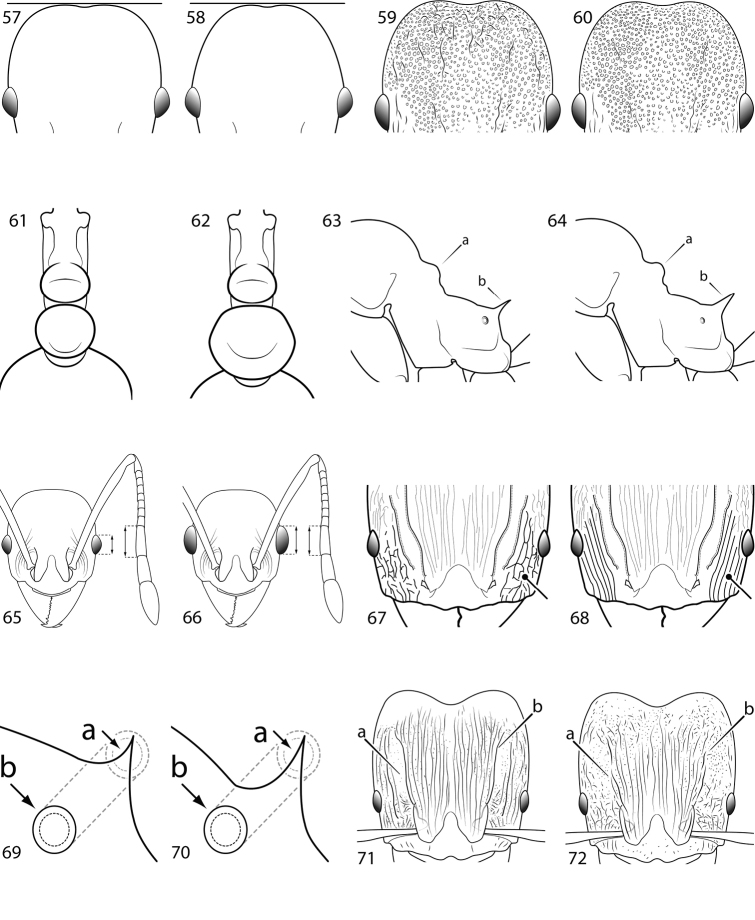


**Table 4.** Illustrated glossary of morphological characters used to diagnose introduced *Pheidole* species. Numbers refer to Figs 1–72. Larger versions of the illustrations are presented in preceding plates. Figures are referred to in the taxonomic keys and species diagnoses.

N	Ilustration	Description
1	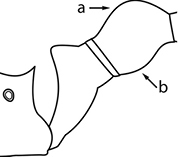	Postpetiole swollen relative to petiole. Postpetiole with a posterodorsal bulge (**a**) and anteroventral bulge (**b**) (major and minor worker). Diagnostic character of *Pheidole megacephala* among introduced *Pheidole*
2	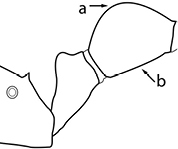	Postpetiole forming a high dorsally bulging dome that is tallest at midpoint (**a**); ventral margin flat to very weakly convex (**b**) (major and minor worker). Diagnostic character of *Pheidole noda* among introduced *Pheidole*
3	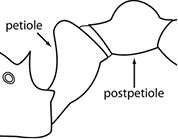	Postpetiole not swollen relative to petiole (major and minor worker). Separates all introduced *Pheidole* species from *Pheidole megacephala* and *Pheidole noda*
4	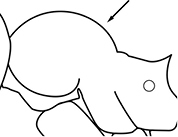	Promesonotum in profile forming a single dome, lacking a distinct mound or prominence on the posterior slope (major worker)
5	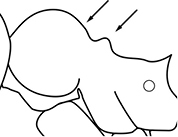	Promesonotum in profile with two convexities, the large anterior dome in addition to a distinct mound or prominence on the posterior slope (major worker)
6	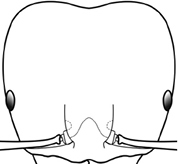	Head heart-shaped (major worker). Diagnostic character of *Pheidole megacephala* among introduced *Pheidole*
7	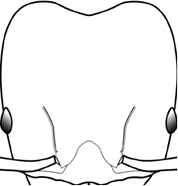	Head subquadrate (major worker)
8	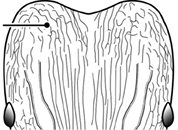	Cephalic dorsum with strong rugoreticulate sculpture, at least on posterolateral lobes (major worker)
9	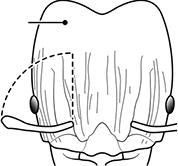	Posterolateral lobes lacking sculpture (including foveolate ground sculpture, carinae and rugae) posterior to maximum extent of antennal scapes in repose (major worker)
10	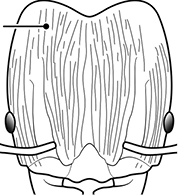	Posterolateral lobes rugose or rugulose (major worker)
11	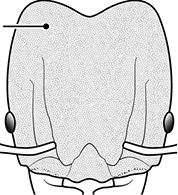	Posterolateral lobes punctate or foveolate (major worker)
12	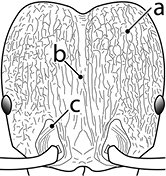	Head almost entirely covered by network of intersecting rugae (a), lacking long, well-organized and parallel longitudinal rugae on the frons (b). Frontal carinae indistinct, quickly becoming integrated into dense rugoreticulum that covers the entire face. Antennal scrobes entirely lacking. Antennal insertions surrounded by deeply excavated pits (c). Diagnostic characters of *Pheidole obscurithorax* major workers among introduced *Pheidole*
13	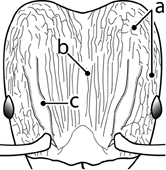	Head rugoreticulate on posterolateral lobes and laterad of frontal carinae (a), but frons dominated by long, well-organized and parallel longitudinal rugae (b). Antennal scrobes indistinct to moderately impressed, but frontal carinae always forming a border capable of accepting the antennal scape (c). Antennal insertions not surrounded by deeply excavated pits. Illustration applies to *Pheidole indica* and *Pheidole fervens*
14	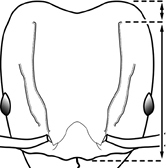	Frontal carinae relatively longer, extend 4/5 distance of head before terminating (major worker). Diagnostic character separating *Pheidole fervens* from *Pheidole indica*
15	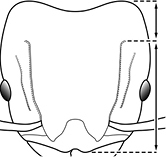	Frontal carinae relatively shorter, extend 3/4 distance of head before terminating (major worker). Diagnostic character separating *Pheidole indica* from *Pheidole fervens*
16	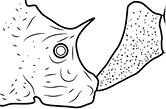	Petiolar node strongly punctate and metapleuron with moderate rugulae and some weak punctation (major worker). Diagnostic character separating *Pheidole proxima* from *Pheidole vigilans*
17	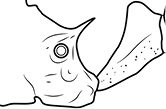	Petiolar node mostly glossy, metapleuron almost completely glossy with strongly reduced carinulae and lacking punctation (major worker). Diagnostic character separating *Pheidole vigilans* from *Pheidole proxima*
18	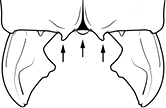	Hypostomal bridge with a small median tooth in addition to a pair of larger inner teeth (major worker). Diagnostic character separating *Pheidole proxima* from *Pheidole vigilans*
19	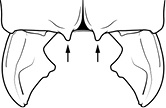	Hypostomal bridge with two well-developed inner teeth but lacking a median tooth (major worker) Diagnostic character separating *Pheidole vigilans* from *Pheidole proxima*
20	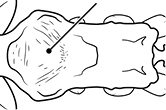	Promesonotal dorsum glossy with thin but distinct subparallel striae running oblique to the longitudinal midline (major worker). Diagnostic character separating *Pheidole rugosula* from other introduced *Pheidole*
21	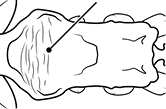	Promesonotal dorsum with distinct transverse striae (major worker). Character present among some species of the *Pheidole flavens* complex, including *Pheidole navigans*
22	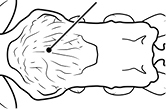	Promesonotal dorsum rugoreticulate with distinct long longitudinal striae in addition to shorter sections of transverse and intersecting striae (major worker). Illustration refers to *Pheidole parva*
23	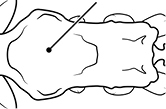	Promesonotal dorsum glossy, lacking foveolate ground sculpture or striae (major worker). Character useful for separating *Pheidole vigilans* and *Pheidole proxima* from *Pheidole rugosula*.
24	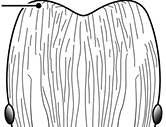	Head with distinct parallel rugae extend from frontal lobes posterior to apices of frontal carinae. Shorter lengths of rugae present across entire posterior region of head and extending to posterior margin in full-face view (major worker). Diagnostic character useful for separating *Pheidole rugosula* from other introduced *Pheidole*, especially those introduced in New Zealand
25	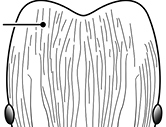	Posterolateral lobes variously sculptured, but posterior head margin always free of distinct rugae or rugoreticulum (major worker). Illustration refers to *Pheidole flavens*, *Pheidole navigans* and other members of the *Pheidole flavens* complex
26	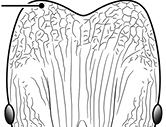	Posterolateral lobes, including posterior head margin, strongly rugoreticulate (major worker). Illustration refers to *Pheidole parva* and character is useful for separating that species from those of the *Pheidole flavens* complex and the *Pheidole punctatissima* clade
27	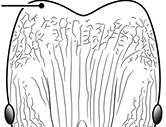	Posterolateral lobes variously sculptured, but posterior head margin always free of rugoreticulum (major worker)
28	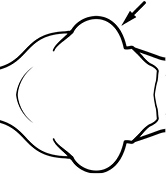	Promesonotum in dorsal view strongly transverse with strongly projecting shoulders (major worker). Illustration refers to *Pheidole parva* and character is useful for separating that species from those of the *Pheidole flavens* complex and the *Pheidole punctatissima* clade
29	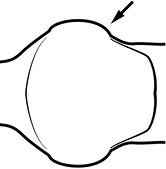	Promesonotum in dorsal view less transverse with weakly projecting shoulders in dorsal view (major worker)
30	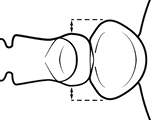	Postpetiole relatively narrow, distinctly less than 2× petiolar width in dorsal view (major worker). Character useful for separating members of the *Pheidole flavens* complex, including *Pheidole flavens* and *Pheidole navigans*, from those of the *Pheidole punctatissima* clade
31	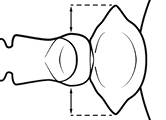	Postpetiole relatively broad, distinctly more than 2× petiolar width in dorsal view (major worker). Character useful for separating members of the *Pheidole punctatissima* clade from those of the *Pheidole flavens* complex, including *Pheidole flavens* and *Pheidole navigans*
32	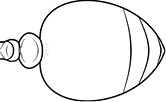	Gaster with entire first tergite glossy (major and minor worker). Character useful for separating members of the *Pheidole flavens* complex, including *Pheidole flavens* and *Pheidole navigans*, from those of the *Pheidole punctatissima* clade
33	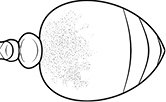	Gaster with at least anterior 1/3 of first tergite matte (major and minor worker). Character useful for separating members of the *Pheidole punctatissima* clade from those of the *Pheidole flavens* complex, including *Pheidole flavens* and *Pheidole navigans*
34	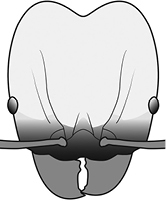	Head bicolored with the yellowish posterior two-thirds contrasting with the darker brown anterior third and rest of body (major worker). Diagnostic character for separating *Pheidole punctatissima* from all other introduced *Pheidole*
35	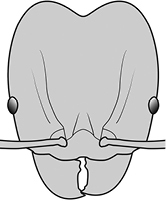	Head uniform in color (major worker). Character used to separate *Pheidole anastasii* and *Pheidole bilimeki* from *Pheidole punctatissima*
36	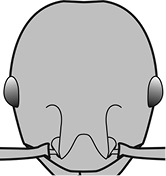	Head predominantly glossy, lacking punctation and or rugae above eye level (minor worker). Character used to separate *Pheidole indica*, *Pheidole fervens*, *Pheidole obscurithorax*, *Pheidole proxima* and *Pheidole vigilans* from all other introduced *Pheidole*.
37	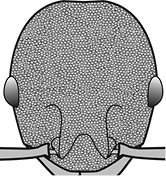	Head, including the area mesad of the frontal carinae, entirely covered by reticulated network of punctures, giving it a dull appearance; if rugae are present they are generally short segments and mostly restricted to posterior portion of head (minor worker). Character used to separate *Pheidole anastasii*, *Pheidole bilimeki*, *Pheidole flavens*, *Pheidole navigans* and *Pheidole parva* from all other introduced *Pheidole*
38	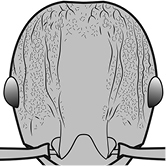	Head with well-defined, long segments of rugae running longitudinally from below the eyes to the posterior head margin. Frontal carinae distinct and reaching towards the posterior head margin, although they may occasionally be interrupted. Punctate ground sculpture present on lateral surfaces of head and just mesad of the frontal carinae, but median portion of head with a large glossy section. Diagnostic characters separating *Pheidole rugosula* from all other introduced *Pheidole*
39	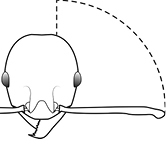	Antennal scapes extremely long, surpassing posterior head margin by more than 2× eye length (minor worker). Diagnostic character separating *Pheidole obscurithorax* from *Pheidole fervens* and *Pheidole indica*
40	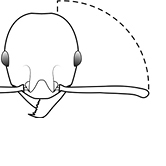	Antennal scapes surpass posterior head margin by approximately same length as eye (minor worker)
41	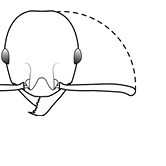	Antennal scapes relatively short, either failing to surpass posterior head margin, or surpassing it by less than the distance of eye length (minor worker)
42	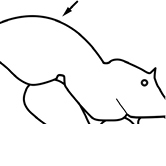	Promesonotum in profile forming a single dome, lacking a distinct mound or prominence on the posterior slope (minor worker)
43	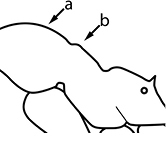	Promesonotum in profile with two convexities, the large anterior dome (**a**) in addition to a distinct prominence on the posterior slope (**b**) (minor worker)
44	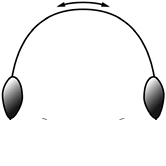	Posterior margin strongly convex in full-face view such that the head outline forms a single unbroken curve from eye to eye (minor worker). Diagnostic character for separating *Pheidole obscurithorax* from *Pheidole fervens* and *Pheidole indica*
45	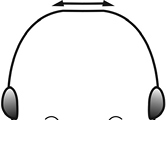	Posterior head margin weakly convex to flat in full-face view (minor worker). Diagnostic character for separating *Pheidole fervens* and *Pheidole indica* from *Pheidole obscurithorax*
46	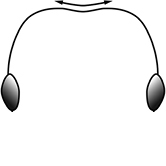	Posterior head margin weakly concave in full-face view (minor worker)
47	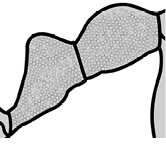	Petiole and postpetiole strongly sculptured laterally. Diagnostic character for separating *Pheidole obscurithorax* from *Pheidole fervens* and *Pheidole indica*
48	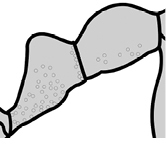	Petiole and postpetiole glossy to very weakly sculptured laterally (minor worker). Diagnostic character for separating *Pheidole fervens* and *Pheidole indica* from *Pheidole obscurithorax*
49	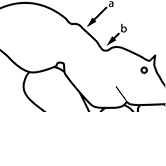	Promesonotal prominence relatively flat (**a**); metanotal depression relatively deep (**b**) (minor worker). Diagnostic character for separating *Pheidole fervens* from *Pheidole indica*
50	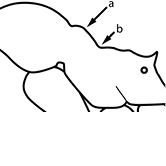	Promesonotal prominence relatively convex (**a**); metanotal depression relatively shallow (minor worker). Diagnostic character for separating *Pheidole indica* from *Pheidole fervens*
51	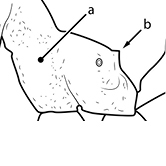	Mesopleuron entirely glossy (**a**); propodeal spines weakly produced and dentiform (**b**) (minor worker). Diagnostic character for separating *Pheidole vigilans* from *Pheidole proxima*
52	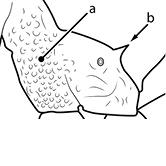	Mesopleuron entirely punctate (**a**); propodeal spines moderately produced and spiniform (**b**) (minor worker). Diagnostic character for separating *Pheidole proxima* from *Pheidole vigilans*
53	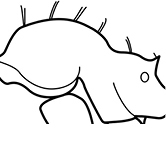	Hairs on mesosoma stout, stiff, of equal length and arranged in pairs (minor worker). Diagnostic character for separating *Pheidole anastasii*, *Pheidole bilimeki* and *Pheidole punctatissima* from *Pheidole flavens*, *Pheidole navigans* and *Pheidole parva*
54	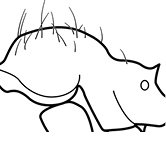	Hairs on mesosoma fine, flexuous, of unequal length and not arranged in pairs (minor worker). Diagnostic character for separating *Pheidole flavens*, *Pheidole navigans* and *Pheidole parva* from *Pheidole anastasii*, *Pheidole bilimeki* and *Pheidole punctatissima*
55	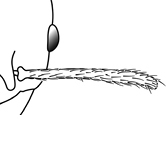	Antennal scapes lack standing hairs (minor worker). Diagnostic character for separating *Pheidole anastasii*, *Pheidole bilimeki* and *Pheidole punctatissima* from *Pheidole flavens*, *Pheidole navigans* and *Pheidole parva*
56	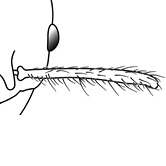	Antennal scapes with erect to suberect hairs (minor worker). Diagnostic character for separating *Pheidole flavens*, *Pheidole navigans* and *Pheidole parva* from *Pheidole anastasii*, *Pheidole bilimeki* and *Pheidole punctatissima*
57	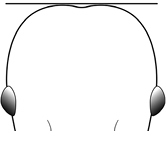	Posterior head margin relatively broad (minor worker). Diagnostic character for separating *Pheidole bilimeki* from *Pheidole anastasii* and *Pheidole punctatissima*
58	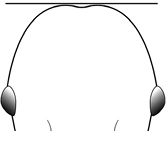	Posterior head margin more narrow (minor worker). Diagnostic character for separating *Pheidole anastasii* and *Pheidole punctatissima* from *Pheidole bilimeki*
59	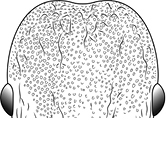	Posterior portion of head with many short to medium length segments of striae distinctly interlaced among punctate ground sculpture (minor worker). Diagnostic character for separating *Pheidole parva* from *Pheidole flavens* and *Pheidole navigans*
60	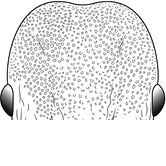	Posterior portion of head lacking many short to medium length segments of striae distinctly interlaced among punctate ground sculpture (minor worker). Diagnostic character for separating *Pheidole flavens* and *Pheidole navigans* from *Pheidole parva*
61	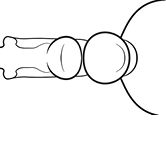	Postpetiole narrow in dorsal view, only slightly broader than petiole (minor worker). Diagnostic character for separating *Pheidole flavens* and *Pheidole navigans* and from *Pheidole anastasii*, *Pheidole bilimeki* and *Pheidole punctatissima*
62	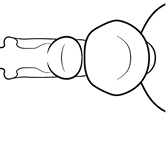	Postpetiole broad in dorsal view, distinctly broader than petiole (minor worker). Diagnostic character for separating *Pheidole anastasii*, *Pheidole bilimeki* and *Pheidole punctatissima* from *Pheidole flavens* and *Pheidole navigans*
63	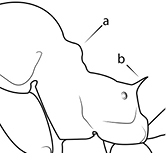	Promesonotal prominence relatively flat (**a**); propodeal spine relatively weak, narrow at base, weakly downcurved at apex (**b**) (major worker). Diagnostic character for separating *Pheidole fervens* from *Pheidole indica*
64	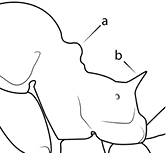	Promesonotal prominence convex and pronounced (**a**); propodeal spine relatively stout, broad at base, straight (**b**) (major worker). Diagnostic character for separating *Pheidole indica* from *Pheidole fervens*
65	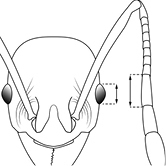	Eye relatively small, eye length distinctly less than length of antennal segment 10 (minor worker). Diagnostic character for separating *Pheidole fervens* from *Pheidole indica*
66	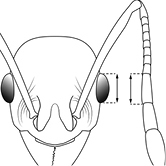	Eye relatively large, eye length subequal to length of antennal segment 10 (minor worker). Diagnostic character for separating *Pheidole indica* from *Pheidole fervens*
67	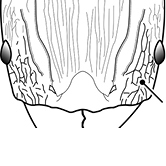	The carinae between eye and mandible are branching and reticulated (major worker). Diagnostic character for separating *Pheidole fervens* from *Pheidole oceanica*
68	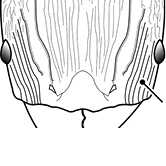	The carinae between eye and mandible are parallel and not reticulated (major worker). Diagnostic character for separating *Pheidole oceanica* from *Pheidole fervens*
69	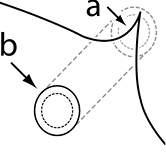	The length of propodeal spine equal to or less than the diameter of propodeal spiracle (minor worker). Diagnostic character for separating *Pheidole fervens* from *Pheidole oceanica*
70	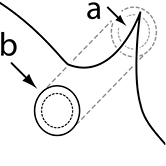	The length of propodeal spine greater than the diameter of propodeal spiracle (minor worker). Diagnostic character for separating *Pheidole oceanica* from *Pheidole fervens*
71	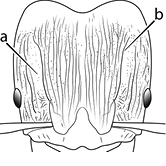	Antennal scrobe distinct and narrow, shallow but capable of receiving the entire antennal scape in repose (**a**); bordered by strong, unbroken frontal carina mesially (**b**); depression marked by a continuous smooth surface entirely (or nearly entirely) uninterrupted by rugulae (major worker). Diagnostic character for separating *Pheidole navigans* from *Pheidole flavens*
72	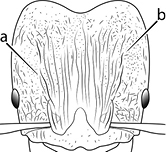	Antennal scrobe broad, ill-defined, incapable of receiving the entire antennal scape in repose (**a**); bordered by relatively weak and interrupted frontal carina mesially (**b**); depression opaque and strongly punctate. Diagnostic character for separating *Pheidole flavens* from *Pheidole navigans*

## Species accounts

### Pheidole anastasii

Taxon classificationAnimaliaHymenopteraFormicidae

Emery

Figs 74 , 88a

Pheidole ***anastasii***. *Pheidole anastasii*Emery 1896: 76 (s.w.) COSTA RICA, Jiménez, [MCSN]. Queen described Forel 1901: 78. Junior synonym of *bilimeki* Mayr: Wilson 2003: 378. Revived status: Longino and Cox 2009: 40. *Nec* M.R. Smith 1933, Naves 1985, Boer and Vierbergen 2008.

#### Diagnosis among introduced *Pheidole*.

Color usually dull yellow to dull brownish yellow. **Major** HW 0.83–1.05, HL 0.90–1.11, SL 0.49–0.62, CI 88–98, SI 50–61 (n=43, Longino pers. comm.). Head uniform in color (Fig. 35); subquadrate (Fig. 7); often entirely punctate (Fig. 11), but portions of posterolateral lobes can be glossy. Posterolateral lobes never with distinct rugae. Promesonotum in profile forming a single dome (Fig. 4). Postpetiole not swollen relative to petiole (Fig. 3). Postpetiole relatively broad; distinctly more than 2× petiolar width in dorsal view (Fig. 31). First gastral tergite with anterior third to entire surface matte. **Minor** HW 0.38–0.50, HL 0.44–0.59, SL 0.44–0.58, CI 82–90, SI 106–120 (n=49, Longino pers. comm.). Head dull, entirely covered by reticulated network of punctures (Fig. 37). Posterior head margin relatively narrow and rounded (Fig. 58). Antennal scapes lack standing hairs (Fig. 55); scapes surpass posterior head margin by a distance equal to or greater than eye (Fig. 40). Promesonotum in profile forming a single dome (Fig. 42), lacking a distinct mound or prominence on the posterior slope. Hairs on mesosoma stout, stiff, of equal length and arranged in pairs (Fig. 53). Postpetiole narrow in dorsal view, only slightly broader than petiole. Gaster with at least anterior 1/3 of first tergite matte (Fig. 33).

#### Identification, taxonomy and systematics.

*Pheidole anastasii*, *Pheidole bilimeki* and *Pheidole punctatissima* all belong to the *Pheidole punctatissima* clade (Economo et al. 2015). These ants are all relatively small species characterized by densely punctate ground sculpture that gives them a dull, matte appearance. Among species treated here, the *Pheidole punctatissima* clade species are most easily confused with those of the closely related *Pheidole flavens* complex. Major and minor workers are most reliably diagnosed from those of the *Pheidole flavens* complex by the relatively broad postpetiole (Fig. 31, major; Fig. 62, minor) and the matte anterior portion of the gaster (Fig. 33) in addition to other characters listed in the key. The minor workers can also be confused with those of Asian native *Pheidole parva*, but can be distinguished by the more uniform and stout mesosomal hairs (Fig. 53), and by the antennal scapes which lack erect hairs (Fig. 55) and tend to surpass the posterior head margin by a distance equal to or greater than eye (Fig. 40). In the Neotropics, there are many native species that closely resemble *Pheidole anastasii* (Wilson 2003), and identification of the minor worker subcaste is especially challenging.

Among introduced members of the clade, the major workers of *Pheidole punctatissima* are immediately distinguished from those of both *Pheidole anastasii* and *Pheidole bilimeki* by the bicolored head (Fig. 33). The minor workers of *Pheidole punctatissima* tend to have narrower posterior head margins and longer antennal scapes than those of *Pheidole anastasii* and *Pheidole bilimeki*. Separating *Pheidole anastasii* from *Pheidole bilimeki* is particularly difficult. They are most reliably distinguished by ecological characteristics, with the former preferring to nest arboreally and the latter preferring to nest under stones or in dead wood. The morphological characters separating these two species are highly variable, but the major workers of *Pheidole anastasii* tend more often towards yellow (*versus* tending towards brown in *Pheidole bilimeki*) and can have relatively wider heads (HW 0.74–1.16 mm *vs.* 0.71–1.07 mm). The minor workers of *Pheidole anastasii* tend to have more narrow heads posteriorly then *Pheidole bilimeki* (Fig. 58 *vs.* Fig. 57) and relatively longer scapes (SI 103–125 *vs.* 95–108). See Longino and Cox (2009) for additional details.

Adding to the already confusing taxonomy separating *Pheidole anastasii* and *Pheidole bilimeki* is the widespread application of the name *Pheidole floridana* Emery to populations across the southern United States. The first record of *Pheidole floridana* from Florida was the type series described by Emery from Coconut Grove (Miami area) in 1895. Smith (1930) recorded *Pheidole floridana* in his original list of Florida ants, and added *Pheidole anastasii* three years later (1933), stating only “This species [*Pheidole anastasii*], which was originally described from Costa Rica, is recorded here for Florida on the basis of information secured from Dr. Wheeler…I have seen the same species in greenhouses in the District of Columbia, New Jersey, and Illinois.” The previous year (1932) Wheeler, who had received type material of *Pheidole floridana* from Emery (Wheeler 1908c), included *Pheidole floridana* and *Pheidole anastasii* in his own list of Florida ants.

Naves (1985) in his study of Florida *Pheidole*, also recognized both species and distinguished *Pheidole anastasii* from *Pheidole floridana* by the matte base of the gaster in the former and the glossy gaster in the latter. Indeed, the type specimens of *Pheidole floridana* from Coconut Grove are consistent with this characterization (CASENT0904424, CASENT0904425). Naves wrote that the Miami area was the only place where he was able to locate *Pheidole floridana*. *Pheidole anastasii*, in contrast, was reported by Naves as widely distributed across the state.

Deyrup et al. (1988), lamenting the taxonomic confusion surrounding *Pheidole floridana*, *Pheidole flavens* and *Pheidole anastasii* in Florida, stated, “Traditionally (Creighton 1950; Smith 1979) the name *Pheidole floridana* has been applied to a widespread upland species that has a distinctive matte area on the base of the first gastral tergite and very evenly rugose head…This is the species we report from the Keys [Florida].” Subsequent reviews of Florida ants have thus excluded *Pheidole anastasii* from their lists (Deyrup 2003; Deyrup et al. 2000; Moreau et al. 2014). Wilson (2003) followed Deyrup in treating all outdoor populations from the United States as *Pheidole floridana*, but conceded that his concept of *Pheidole floridana* could represent a northern geographic variant of *Pheidole bilimeki* or an endemic species modified by intergradation with a *Pheidole bilimeki* immigrant population.

With respect to all outdoor North American records, we follow Wheeler (1932), Smith (1933), and Naves (1985) in treating the localized glossy-gaster *Pheidole floridana* as distinct from the widespread matte-gaster species referred to as *Pheidole anastasii* by the aforementioned authors. However, the relatively short scapes and posteriorly broad heads of the minor workers, together with the habitat and nesting preferences of the matte-gaster species suggests the name *Pheidole bilimeki* Mayr more accurately applies to this widespread taxon than does *Pheidole anastasii* Emery. The issue is discussed in further detail under the *Pheidole bilimeki* section.

#### Biology.

*Pheidole anastasii*, named by Emery on behalf of Sig. Anastasio Alfaro, is a Neotropical species that is occasionally found indoors beyond its native range. Although at least some arboreal colonies appear to be polydomous, *Pheidole anastasii* is a low-impact adventive that has thus far shown little capacity for becoming a significant invader. The biology of *Pheidole anastasii*, especially across its native range in Costa Rica and in comparison to *Pheidole bilimeki* was reviewed by Longino and Cox (2009). The species was noted as being among the most abundant ants in the low arboreal forest understory of La Selva Biological Station (Costa Rica). Although tolerant of disturbance, *Pheidole anastasii* requires some vegetation cover and does not occur in open areas. All collections reviewed by Longino and Cox were from wet forest habitats. Most were from below 500 m elevation, but several ranged to a maximum of 1200 m. The propensity for the species to be inadvertently transported to greenhouses across the world is predicted by its arboreal foraging and nesting habits. Longino and Cox (2009) observed the species nests in almost any kind of cavity or sheltered space, including live stems, and that workers often build galleries and tunnels with carton or earthen construction. The species was reported to occur in lowland second growth, evergreen forest, coffee plantation, limestone, ravine, mixed hardwood-pine forest, wet forest, on karst, and cloud forest. It was also reported to nest in dead sticks and branches on or above the forest floor, under bark flaps on tree trunks, beneath epiphytes and under stones.

#### Distribution.

*Pheidole anastasii* is a Neotropical native that ranges from Mexico to southern Central America or northern South America. We consider many of the outdoor records of *Pheidole anastasii* from the southern United States to refer instead to *Pheidole bilimeki* (see discussion above). There are, however confirmed records of the species from heated indoor locations – especially greenhouses. In North America there are records from hothouses in Washington D.C. and New York (Longino and Cox 2009), and also from Massachusetts. In Europe, the Netherlands occurrences reported as *Pheidole anastasii* by Boer and Vierbergen (2008) refer to *Pheidole bilimeki* (Boer, pers. comm.). The records from Denmark and Norway might also refer to *Pheidole bilimeki*, but until specimens can be examined we follow the authors’ use of *Pheidole anastasii* (Birkemoe and Aak 2008; Lomholdt 1986).

#### Risk statement.

*Pheidole anastasii* is a synanthropic species with a high tolerance for habitat disturbance. It is occasionally found in human habitations and in greenhouses. There is little indication that is causes significant impact to agricultural systems or native ecosystems. The species is a quarantine risk, and is thought to be transported with fresh plant material.

### Pheidole bilimeki

Taxon classificationAnimaliaHymenopteraFormicidae

Mayr

Figs 75 , 88b

Pheidole ***bilimeki***. *Pheidole bilimeki*Mayr 1870b: 985 (s.) MEXICO (Bilimek) [NHMW]. Lectotype (s.) designated: Wilson 2003: 378. *Nec*Donisthorpe 1946, Wittenborn and Jeschke 2011.
Pheidole *deplanata*. Pheidole floridana var. deplanataPergande 1896: 883 (s.w.) MEXICO, Tepic (Eisen and Vaslit) [USNM]. Junior synonym of *bilimeki*Wilson 2003: 378.
Pheidole *antoniensis*. Pheidole floridana var. antoniensisForel 1901b: 364 (s.w.) COLOMBIA, San Antonio, Sierra Nevada de Santa Marta (Forel) [MHNG]. Junior synonym of *bilimeki*: Wilson 2003: 378.
Pheidole *annectens*. Pheidole punctatissima subsp. annectens Wheeler, W.M. 1905: 93 (s.) BAHAMAS, Mangrove Key, Andros Island (Wheeler) [MCZC]. Junior synonym of *bilimeki*: Wilson 2003: 378.
Pheidole *insulana*. Pheidole punctatissima subsp. insulana Wheeler, W.M. 1905: 93 (s.w.) BAHAMAS Southern Bight, Andros Islands; BAHAMAS, Blue Hills, New Providence Island (Wheeler) [MCZC]. Junior synonym of *bilimeki*: Wilson 2003: 378.
Pheidole *venezuelana*. Pheidole anastasii var. venezuelanaForel 1905b: 159 (s.m.) VENEZUELA, Caracas (Meinert) [MHNG]. Junior synonym of *bilimeki*: Wilson 2003: 378.
Pheidole *johnsoni*. Pheidole anastasii var. johnsoni Wheeler, W.M. 1907: 272 (s.w.m.) HONDURAS, Manatee (Johnson) [MCZC]. Junior synonym of *bilimeki*: Wilson 2003: 378.
Pheidole *ares*. Pheidole floridana subsp. aresForel 1908: 57 (s.w.m.) COSTA RICA, Cote du Tablazo, 1500 m; COSTA RICA, San Juan de Tobozi, 1400 m (Biolley) [MHNG]. Junior synonym of *bilimeki*: Wilson 2003: 378.
Pheidole *lauta*. *Pheidole lauta* Wheeler, W.M. 1908c: 470 (s.w.q.m.) U.S.A. Subspecies of *floridana*: Creighton 1950: 179. Junior synonym of *floridana*: Gregg 1959: 21. See also Wilson 2003: 424. **n. syn.**
Pheidole *cellarum*. Pheidole anastasii var. cellarumForel 1908: 55 (s.w.) greenhouses in Zurich (SWITZERLAND), Kew (GREAT BRITAIN), Dresden (GERMANY) [MHNG]. Description of queen (as *Pheidole anastasii*, based on material from Guatemala intercepted at Hamburg; material labeled incorrectly as *cellarum* types in Forel collection): Forel 1901a: 78. Description of queen in key: Forel 1915: 34. Junior synonym of *bilimeki*: Wilson 2003: 378.
Pheidole *rectiluma*. *Pheidole rectiluma*Wilson 2003: 493 (s.w.) NICARAGUA, Hotel Selva Negra, 139 km north of Matagalpa, 1200 m (Kugler & Hahn). Junior synonym of *bilimeki*: Longino 2009: 16.

#### Diagnosis among introduced *Pheidole*.

Color usually red brown, rarely yellow brown. **Major** HW 0.75–1.04, HL 0.79–1.13, SL 0.44–0.57, CI 87–97, SI 50–65 (n=39, Longino pers. comm.). Head uniform in color (Fig. 35); subquadrate (Fig. 7); often entirely punctate (Fig. 11), but portions of posterolateral lobes can be glossy. Posterolateral lobes never with distinct rugulae. Promesonotum in profile forming a single dome (Fig. 4). Postpetiole not swollen relative to petiole (Fig. 3). Postpetiole relatively broad; distinctly more than 2× petiolar width in dorsal view (Fig. 31). First gastral tergite with anterior third to entire surface matte. **Minor** HW 0.42–0.52, HL 0.47–0.59, SL 0.40–0.54, CI 83–93, SI 88–108 (n=38, Longino pers. comm.). Head, including the area mesad of the frontal carinae, entirely covered by reticulated network of punctures, giving it a dull appearance (Fig. 37). Posterior head margin relatively broad and flat (Fig. 57). Antennal scapes lack standing hairs (Fig. 55); surpass posterior head margin by a distance equal to or greater than eye (Fig. 40). Promesonotum in profile forming a single dome (Fig. 42), lacking a distinct mound or prominence on the posterior slope. Hairs on mesosoma stout, stiff, of equal length and arranged in pairs (Fig. 53). Postpetiole narrow in dorsal view, only slightly broader than petiole. Gaster with at least anterior 1/3 of first tergite matte (Fig. 33).

#### Identification, taxonomy and systematics.

*Pheidole bilimeki* is a member of the Neotropical *Pheidole punctatissima* clade, together with *Pheidole anastasii* and *Pheidole punctatissima* (Economo et al. 2015). Among species treated here, it is easily confused with the aforementioned and members of the *Pheidole flavens* complex. Minor workers can also be confused with those of *Pheidole parva*. See section under *Pheidole anastasii* for identification notes. In the southeastern United States, *Pheidole bilimeki* is often confused with *Pheidole floridana* Emery, which is discussed in more detail below. In the Neotropics, there are many native species that closely resemble *Pheidole bilimeki* (Wilson 2003).

We propose the synonymy of *Pheidole lauta* Wheeler to be transferred from *Pheidole floridana* to *Pheidole bilimeki*. In his original description Wheeler (1908c) wrote, “…the worker has the base of the gaster opaque whereas this is shining in the specimen of *floridana* given me by Prof. Emery.” The description and the photographs we have examined of the type specimens all agree with the concept of *Pheidole bilimeki* used here and in Longino and Cox (2009).

Should *Pheidole floridana* therefore be synonymized under *Pheidole bilimeki*? Wilson (2003) offered that the former might represent the northernmost population of the latter, and recent phylogenetic analyses (Economo et al. 2015; Moreau 2008) show these two as sibling taxa. Based on the results of her analysis, Moreau (2008) found that her samples of *Pheidole bilimeki* (Costa Rica, RA0162) and putative *Pheidole floridana* (Florida, RA0331) were each other’s closest relatives, and that this pair was sister to *Pheidole anastasii* (Costa Rica). The result is also supported by Economo et al. (2015), which found a shallow divergence separating *Pheidole bilimeki* from putative *Pheidole floridana*, especially compared to the deep divergence separating these sister taxa from *Pheidole anastasii*. Moreau (2008) concluded that in order for *Pheidole anastasii* to be a valid member of *Pheidole bilimeki*, as proposed by Wilson (2003), *Pheidole floridana* would also have to be accepted as a synonym of *Pheidole bilimeki*.

We suggest that this conundrum stems from the common misapplication of the name *Pheidole floridana* (a shiny gaster species) to collections of what are in fact the North American population of *Pheidole bilimeki* (a matte gaster species). Naves (1985) came to a similar conclusion in his revision of the *Pheidole* of Florida, *“P. floridana* seems to be confined to southeast Florida in the Miami area. This is the only place where I was able to locate this species. Due to its close relationship to *Pheidole anastasii* the latter has been misidentified as *Pheidole floridana* many times, thus, mistakenly extending the supposed range of *P. floridana. P. anastasii* is actually the species widely distributed in Florida, while *floridana* is absent or at least must be rare in most of the state.”

One explanation for the confusing phylogenetic results is that RA0331 actually refers to *Pheidole bilimeki* Mayr, and that true members of *Pheidole floridana* Emery from the Miami area were not included in the aforementioned phylogenetic analyses. The samples of RA0331 were collected in central Florida from Polk County, well outside the Miami area from which the *Pheidole floridana* Emery is known (Naves 1985). Deyrup, who collected and identified the specimens of RA0331, has previously (2003; 1988; 1989) applied the name *Pheidole floridana* to matte gaster specimens that earlier authors (Naves 1985; Smith 1933; Wheeler 1932) would have considered *Pheidole anastasii* Emery, and that we consider *Pheidole bilimeki* Mayr.

To properly ascertain the taxonomic status of *Pheidole floridana* Mayr we suggest a future phylogenetic analysis that includes specimens matching the type material of *Pheidole floridana*, preferably from the Miami area. If there is evidence supporting the conspecificity of samples matching our concept of *Pheidole bilimeki*, then the validity of *Pheidole floridana* Emery must be revaluated. If, rather, the *Pheidole floridana* samples are heterospecific with respect to *Pheidole bilimeki*, then there are at least two hypotheses that could explain this result. One is that *Pheidole floridana* is endemic to Florida. The second, perhaps more compelling albeit ironic explanation, would propose the Miami population of *Pheidole floridana* is conspecific with a Neotropical species inadvertently introduced to Florida. Miami is a major shipping port and was the gateway for many introduced ants over the past two centuries (Deyrup et al. 2000).

#### Biology.

The taxonomic confusion surrounding whether published accounts refer to our proposed concept of *Pheidole bilimeki*, or instead to either *Pheidole floridana* or *Pheidole anastasii*, makes it difficult to ascertain the natural history of the species. The following account given by Longino and Cox (2009), however, refers definitively to *Pheidole bilimeki*. They report that *Pheidole bilimeki* is a common species in open, recently or frequently disturbed habitats. In Costa Rica it occurs in lowland dry forest, lowland wet forest, and montane habitats to about 1500 m elevation. It is a common ant of roadsides, nesting under stones or in dead fence posts. It is a frequent pest ant in houses and is a common ant at baits in second growth dry forest vegetation in seasonally dry Guanacaste Province. It can also be abundant and dominant in large disturbances deep within primary forest reserves. We tentatively treat the account given by Wilson (2003) for *Pheidole floridana* as referring to the North American population of *Pheidole bilimeki*. That account stated that winged reproductives have been found in nests during September and October, and that the species occurs in a variety of woodland habitats, nests in soil, litter, and rotten wood, and in both xeric and mesic situations. It also noted the observation of Stefan Cover that colonies are monogynous, may contain 1000 or more ants, and are sometimes polydomous. Cover observed that the species is omnivorous, but does not appear to harvest seeds (but see Naves 1985). Naves (1985) discussed the biology of *Pheidole bilimeki* (as *Pheidole anastasii*) in Florida. He found the species most often nesting under the bark at the base of pines or along the roots, but occasionally found it nesting in the soil. The colonies he observed supported over 600 workers with a 5:1 ratio of minors to majors. Mature colonies were monogynous, although in laboratory conditions colonies that lost their original queen would accept other conspecific queens. Several colonies were discovered with two or three founding females, but laboratory experiments found that one would kill the others before the rearing of the first brood. Naves also recorded that the species feeds on seeds, fruits, and scavenges on small dead arthropods and is predaceous on small live arthropods.

#### Distribution.

*Pheidole bilimeki* is a Neotropical native that ranges from northern South America to southern North America and across the Caribbean. The records included here from the southern United States have previously been treated as *Pheidole anastasii* and *Pheidole floridana* (see discussion). *Pheidole bilimeki* was not reported from Florida until 1932 (Wheeler). While it is possible that the penetration of *Pheidole bilimeki* into the southern United States represents a recent dispersal event, even one that has been anthropogenically facilitated, there are several reasons for considering *Pheidole bilimeki* as native to the region. Firstly, the range of North American populations appear contiguous with those of Mexico and the Caribbean, and gene flow among them is probable. Secondly, populations from Florida are known to host two parasites, a mermithid that parasitizes workers, and a hymenopteran parasite species of the genus *Orasema* (Naves 1985). *Pheidole bilimeki* has been recorded from greenhouses in Illinois and Ohio in North America. The species has also been found indoors and greenhouses across Europe, including the Netherlands (Boer and Vierbergen 2008), Germany (Forel 1908), Great Britain (Forel 1908), Ireland (Stelfox 1927), and Switzerland (Forel 1908). The only occurrence of *Pheidole bilimeki* in Jamaica is reported by Wilson (2003). Although the species might occur there, it is also possible that Wilson was referring to *Pheidole jamaicensis* Wheeler. The single Mauritius occurrence is of a single minor worker examined by Donisthorpe (1946), but this specimen more likely refers to the superficially similar *Pheidole parva* which is widespread across the island and its neighbors in the Indian Ocean.

#### Risk statement.

*Pheidole bilimeki* is a synanthropic species with a high tolerance for habitat disturbance. It is occasionally found indoors, especially in greenhouses. There is little indication that is causes significant impact to agricultural systems or native ecosystems.

### Pheidole fervens

Taxon classificationAnimaliaHymenopteraFormicidae

F. Smith

Figs 76 , 88c

Pheidole ***fervens***. *Pheidole fervens* Smith, F. 1858: 176 (s.) SINGAPORE (BMNH). Lectotype (s.) (CASENT0901520) designated: Fischer and Fisher 2013: 322.
Pheidole *pungens*. *Solenopsis pungens*Smith 1861: 48. INDONESIA, Menado, Sulawesi (A.R. Wallace). Combination in *Pheidologeton*: Donisthorpe 1932: 469; in *Pheidole*: Bolton 1995: 328. Junior synonym of *Pheidole fervens*; lectotype (s.) designated: Eguchi 2004b: 198.
Pheidole *javana*. *Pheidole javana* Mayr, 1867: 66 (s.w.) INDONESIA, Batavia [Jakarta], Java. Junior synonym of *Pheidole fervens*: Wilson and Taylor 1967: 45. Lectotype (s.) designated: Eguchi 2004b.
Pheidole *cavannae*. *Pheidole cavannae* Emery 1887: 464 (footnote) (s.) NEW CALEDONIA. Subspecies of *Pheidole oceanica*: Emery 1914: 401. Junior synonym of *Pheidole fervens*: Wilson and Taylor 1967: 45.
Pheidole *dharmsalana*. Pheidole javana var. dharmsalanaForel 1902c: 184, 198 (s.) INDIA, Dharmsala (Sage). [Also described as new by Forel 1902: 546]. Subspecies of *Pheidole fervens*: Bolton 1995: 320. Junior synonym of *Pheidole fervens*; lectotype (s.) designated: Eguchi 2004b: 198.
Pheidole *amia*. *Pheidole amia*Forel 1912: 60 (s.w.) TAIWAN, Takao [Kaohsiung]. Junior synonym of *Pheidole fervens*; lectotype designated: Eguchi 2004b: 197.
Pheidole *dolenda*. Pheidole javana var. dolendaForel 1912: 60 (s.w.) TAIWAN, Akau. Subspecies of *Pheidole fervens*: Bolton 1995: 320. Junior synonym of *Pheidole fervens*; lectotype designated: Eguchi 2004b: 198.
Pheidole *nigriscapa*. Pheidole oceanica subsp. nigriscapa Santschi, 1928: 48 (s.w.) SAMOA, Apia, Upolu (H. Swale). Junior synonym of *Pheidole fervens*: Wilson and Taylor 1967: 45.
Pheidole *tahitiana*. Pheidole oceanica subsp. nigriscapa var. tahitiana Santschi [in Cheesman and Crawley 1928]: 516. FRENCH POLYNESIA, Tahiti. Unavailable name; material referred to *Pheidole fervens* by Wilson and Taylor 1967: 45.
Pheidole *desucta*. Pheidole javana var. desucta Wheeler, W.M. 1929: 2 (s.w.q.) CHINA, Back Liang. Subspecies of *Pheidole fervens*: Bolton 1995: 320. Junior synonym of *Pheidole fervens*: Eguchi 2001a: 53. Lectotype designated: Eguchi 2004b.
Pheidole *soror*. Pheidole javana var. sororSantschi 1937: 369 (s.w.) TAIWAN, Hokuto. Subspecies of *Pheidole fervens*: Bolton 1995: 330. Junior synonym of *Pheidole fervens*; lectotype designated: Eguchi 2004b: 198.
Pheidole *azumai*. Pheidole nodus st. azumaiSantschi 1941: 274 (s.w.) JAPAN, Tennooji, Osaka. Junior synonym of *Pheidole fervens*; lectotype designated: Eguchi 2004b: 198.

#### Diagnosis among introduced *Pheidole*.

Color yellowish brown to dark brown. **Major** HW 1.13–1.44, HL 1.13–1.56, SL 0.80–0.95, CI 92–100, SI 61–71 (n=15, Eguchi 2001a; 2008; Fischer and Fisher 2013). Head square to subquadrate (Fig. 7); rugoreticulate on posterolateral lobes and laterad of frontal carinae (Fig. 13a), but frons dominated by long, well-organized and parallel longitudinal rugae (Fig. 13b). Antennal scrobes indistinct to moderately impressed, but frontal carinae always forming a border capable of accepting the antennal scape (Fig. 13c). Frontal carinae relatively longer, extend 4/5 distance of head before terminating (Fig. 14). Promesonotum in profile with two convexities (Fig. 5), the large anterior dome in addition to a distinct mound or prominence on the posterior slope. Postpetiole not swollen relative to petiole (Fig. 3). **Minor** HW 0.52–0.63, HL 0.66–0.73, SL 0.77–0.87, CI 79–88, SI 133–154 (n=16, Eguchi 2001a; 2008; Fischer and Fisher 2013). Head predominantly glossy (Fig. 36), lacking punctation or rugulae above eye level. Posterior head margin weakly convex to flat in full-face view (Fig. 45). Antennal scapes long (e.g. Fig. 39), but not surpassing the posterior head margin by more than 2× eye length. Promesonotum in profile with two convexities, the large anterior dome (Fig. 43a) in addition to a distinct prominence on the posterior slope (Fig. 43b). Promesonotal prominence relatively flat (Fig. 49a). Metanotal depression relatively deep (Fig. 49b). Petiole and postpetiole glossy to very weakly sculptured laterally (Fig. 48). Postpetiole not swollen relative to petiole (Fig. 3).

#### Identification, taxonomy and systematics.

*Pheidole fervens* is a medium to large sized species with long limbs. It belongs to the *Pheidole fervens* clade along with its Australasian congeners *Pheidole cariniceps*, *Pheidole hospes*, *Pheidole impressiceps*, and *Pheidole oceanica* (Economo et al. 2015). The major workers have strong cephalic rugulae that become reticulated towards the posterior of the head and the minor workers have completely glossy heads with very long antennal scapes. Majors and minors of the species can be separated from those of *Pheidole megacephala* and *Pheidole noda* by the postpetiole which is not swollen compared to the petiole (Fig. 3), and the promesonotum which has the large anterior dome in addition to a distinct prominence on the posterior slope (Fig. 5, major; Fig. 43, minor). The minors of *Pheidole fervens* can also be separated from those of *Pheidole megacephala* by their larger size and longer antennal scapes (Fig. 39). The majors are easily distinguished from *Pheidole megacephala* by the very sculptured head (Fig. 13).

Among species treated here, *Pheidole fervens* is most easily confused with its close relative, *Pheidole indica*, and the characters used to separate these two are subtle. For both subcastes, the promesonotal prominence is flatter in *Pheidole fervens* (Fig. 49a, minor; Fig. 63a, major) compared to that of *Pheidole indica* (Fig. 50a, minor; Fig. 64a, major). The eyes of *Pheidole fervens* minors (Fig. 65) are relatively smaller than those of *Pheidole indica* minors (Fig. 66), especially in comparison to antennal segment 10. The propodeal spines of *Pheidole fervens* are weaker, narrower, and more downcurved in majors of *Pheidole fervens* (Fig. 63b) compared to those of *Pheidole indica* (Fig. 64b). Readers are referred to Eguchi (2004b; 2008) for characters used to separate *Pheidole fervens* and *Pheidole indica* from their Asian congeners.

In the Pacific Island region *Pheidole fervens* is often confused with the nearly identical *Pheidole oceanica*, which is native to that region. The carinae between eye and mandible are branching and reticulated in the majors of *Pheidole fervens* (Fig. 67), versus parallel and not reticulated in those of *Pheidole oceanica* (Fig. 68). This character was erroneously reversed in the key provided in Sarnat and Economo (2012). The minors are more difficult to separate, but in *Pheidole fervens* the length of propodeal spine is equal to or less than the diameter of propodeal spiracle (Fig. 69), whereas in *Pheidole oceanica* it is greater (Fig. 70).

#### Biology.

For such a ubiquitous species across its native and introduced range, very little is known about the biology of *Pheidole fervens*. It is a synanthropic species with a high tolerance for disturbance (Eguchi 2004b; Fischer and Fisher 2013; Martínez 1996), but can also thrive under some degree of canopy cover (Morrison 1996; Sarnat and Economo 2012). In Fiji, where it is likely a recent colonizer, it was collected most frequently in human dominated landscapes between 0–800 m, although several collections were also made from primary forest at low elevations. In Hawaii, where it is definitely an introduced species, it is more abundant locally in wet regions than *Pheidole megacephala* (Gruner et al. 2003) and occurs in the hot lowlands only below 900 m (Reimer 1994). In the Philippines, *Pheidole fervens* is found in irrigated lowlands (rice fields) where it is characterized as dominant species capable of displacing *Solenopsis geminata* in the dry season (Way et al. 1998). In Japan it occurs in open land grading to forest edge (Harada et al. 2009; Ogata 1981). *Pheidole fervens* recruits in large numbers to bait and forages both on the ground and on vegetation (Sarnat and Economo 2012). Baiting experiments on Pacific Islands found that *Pheidole fervens* can act as a numerically and behaviorally dominant species capable of excluding other invasive ant species (including *Anoplolepis gracilipes*, *Nylanderia bourbonica*, and *Tetramorium bicarinatum*) from baits (Morrison 1996). Although foragers can be slow to discover food resources, once found they can recruit in large numbers and displace competing species (Morrison 1996). Experiments in China suggest that *Pheidole fervens* can provide some degree of biotic resistance to the Red Imported Fire Ant (*Solenopsis invicta*) by acting in groups to dismember the limbs of individual fire ants (Chen et al. 2011). Martínez (1996) suggested the California population of *Pheidole fervens* was polydomous, and Passera (1994) suggested the Hawaii population is unicolonial and polygynous, but detailed colony-level studies of the species are required to verify these claims. Wittenborn and Jeschke (2011) attributed their assertion that *Pheidole fervens* practices dependent colony founding to Harris et al. (2005a), but we were unable to find any reference to colony foundation in that report and cannot substantiate their evidence.

#### Distribution.

We consider *Pheidole fervens* as native to a broad expanse of the Indo-Malay region spanning from India east to the Philippines and south to the islands west of New Guinea. This is a broad and admittedly arbitrary boundary, but a more precise circumscription of the native range requires a population-level analysis outside the scope of the present study. In particular, it is difficult to ascertain the extent of its range into the Pacific Island region prior to the Anthropocene. The only known occurrence of *Pheidole fervens* from New Guinea was a single record from the westernmost part of the island (Emery 1887b). East of New Guinea, however, the species is established on nearly all islands of the Pacific, including those which were uninhabited by any ant prior to human arrival. Although it is quite possible that *Pheidole fervens* reached some of these islands without human assistance – especially those between Taiwan and mainland Japan – we treat these as introduced populations. And although established on Mauritius, the species is rarely encountered there and is currently known from only two localities (Fischer and Fisher 2013). The only record of introduction in North America is a California population that established nests in cracks of roads and along the sides of buildings in a two-block area of downtown Los Angeles (Martínez 1996). *Pheidole fervens* has been collected from greenhouses in the Netherlands (Boer and Vierbergen 2008), and is frequently intercepted by quarantine inspections (Ward et al. 2006).

#### Risk statement.

*Pheidole fervens* can be a dominant species where it is locally abundant. Although few studies have measured the effect of *Pheidole fervens* on native ecosystems, we predict that it could negatively impact native arthropods. We were unable to find documentation on the effect of *Pheidole fervens* on agricultural systems, but it can be among the most abundant ant species in irrigated lowland crop systems such as rice fields. *Pheidole fervens* can also be an indoor nuisance species (Wilson and Taylor 1967), but is not a risk for structural damage. According to New Zealand records, the species is among the most commonly intercepted ants in that country (Ward et al. 2006). Sixty-nine percent of the interceptions were in freight from Fiji (> 92% from the Pacific Islands). Interceptions were mostly in fresh produce (69%) and cut flowers (8%). *Pheidole fervens* was also intercepted multiple times in air passengers’ luggage and shipping containers. The species could become more globally widespread in the future.

### Pheidole flavens

Taxon classificationAnimaliaHymenopteraFormicidae

Roger

Figs 77 , Fig. 88d

Pheidole ***flavens***. *Pheidole flavens*Roger 1863a: 198 (s.w.q.) CUBA. Wheeler, W.M. 1905: 92 (m.). Neotype designated: Barrajagua, Las Villas, CUBA (E.O. Wilson): Wilson 2003: 419.
Pheidole *tuberculata*. Pheidole exigua var. tuberculataMayr 1887: 585 (s.) St. Catharina, BRAZIL. Subspecies of *flavens*: Emery 1894: 157. Junior synonym of *flavens*: Wilson 2003: 419.
Pheidole *vincentensis*. Pheidole flavens var. vincentensisForel 1893a: 411 (s.w.q.m.) SAINT VINCENT. Junior synonym of *flavens*: Wilson 2003: 419.
Pheidole *gracilior*. Pheidole flavens r. graciliorForel 1901a: 78 (s.w.q.) GERMANY (intercepted in quarantine, from West Indies). Junior synonym of *flavens*: Wilson 2003: 419.
Pheidole *haytiana*. Pheidole flavens var. haytiana Forel 1907: 6 (w.) HAITI, Port-au-Prince (Keitel). Wheeler, W.M. & Mann, 1914: 24 (s.q.m.). Junior synonym of *flavens*: Wilson 2003: 419.
Pheidole *spei*. Pheidole flavens st. speiSantschi 1930: 77 (s.w.) CUBA, Pinar del Rio, Punta Esperanza, 4.i.2030, 7 s., 10 w. (Bierig). Junior synonym of *flavens*: Wilson 2003: 419.
Pheidole *aechmeae*. Pheidole floridana subsp. aechmeae Wheeler, W.M. 1934: 166 (s.w.) MEXICO, Camaron near Mirador, Vera Cruz, in *Aechmea bracteata*, No. 472 (Skwarra). Junior synonym of *flavens*: Wilson 2003: 419.
Pheidole *greggi*. *Pheidole greggi* Naves, 1985: 62, figs. 21, 45, 57 (s.w.) U.S.A., Miami, Florida, 19.xii.1945 (W.F. Buren). Junior synonym of *flavens*: Wilson 2003: 419.

#### Diagnosis among introduced *Pheidole*.

See notes under *Pheidole flavens*-complex. Neotype major: HW 0.72, HL 0.74, SL 0.42, CI 103, SI 58. Paraneotype minor: HW 0.34, HL 0.42, SL 0.34, CI 124. SI 100. Non-type measurements, major: HW 0.68–0.83, HL 0.74–0.88, SL 0.39–0.42, CI 87–97, SI 52–59. Non-type measurements, minor: HW 0.34–0.45, HL 0.39–0.49, SL 0.34–0.42, CI 81–93, SI 89–104.

#### Identification, taxonomy and systematics.

*Pheidole flavens* belongs to the *Pheidole flavens*-complex along with a putatively large number of other nominal taxa. However, the *Pheidole flavens* group as conceived by Wilson (2003) is now known to be polyphyletic (Economo et al. 2015; Moreau 2008). Readers are referred to the *Pheidole flavens*-complex for additional discussion of identification, taxonomy and systematics. The taxonomy of *Pheidole flavens* and its close relatives remains in a state of confusion. It is beyond the scope of the present study to resolve this issue, but we contribute the following discussion as a step towards that goal.

*Pheidole flavens* was originally described by Roger from Cuba, but the type material is considered to be lost. Wilson (2003) designated a neotype from Cuba and synonymized a total of eight nominal taxa with *Pheidole flavens*. Of these, *Pheidole greggi* Naves (Florida) and perhaps Pheidole flavens st. spei Santschi (Mexico) are most similar to the Cuban neotype. They, together with the types of Pheidole moerens subsp. creola, are the only specimens examined thus far that have clearly reticulated rugulae posterior to the scrobes of major workers. Naves’ (1985: fig. 55) concept of *Pheidole flavens* Roger, at least as evidenced by his figures and descriptions, more closely matches our concept *Pheidole navigans*, a species that is spreading across the southeastern United States. The syntype major of Pheidole flavens var. vincentensis Forel differs substantially from the neotype in that the head is completely glossy between the rugulae, which are themselves entirely longitudinal and do not extend far beyond the maximum extent of the antennal scapes in repose. These characters make it at least superficially more similar to *Pheidole moerens* and *Pheidole navigans*. Pheidole flavens r. gracilior and *Pheidole navigans* were both described by Forel from workers intercepted at a Hamburg quarantine facility, which is testament to the dispersive ability of this complex. The syntype major of the latter species and that of Pheidole floridana subsp. aechmeae Wheeler, also described from Mexico, are quite similar. Pheidole exigua var. tuberculata Mayr has the strongly convex head and promesonotal dome of *Pheidole exigua* Mayr, and also exhibits tuberculate angles on the mesonotal declivity. Type specimens of Pheidole flavens var. haytiana Forel were not examined for this study.

The only material from outside Central America and the Caribbean that we were able to confirm as matching the Wilson’s neotype was from Florida. The Florida populations referred to here as *Pheidole flavens* and *Pheidole navigans* are almost certainly heterospecific. We suspect that Nearctic records of *Pheidole flavens* outside of Florida such as those reported from Louisiana (Colby and Prowell 2006; Dash and Hooper-Bùi 2008) refer to either *Pheidole bilimeki* or the species we are treating as *Pheidole navigans* in the southeastern USA.

#### Biology.

The biology of *Pheidole flavens*, as currently conceived, was reviewed by Wilson (2003) with contributing observations by Jack Longino. The species prefers rotting wood, but also nest beneath the bark of trees, in dead knots on tree trunks, in sod on rocks, in the soil beneath stones, and in epiphyte masses. In the Caribbean it is recorded from forests and thickets from sea level to 900 m, and in Costa Rica it occurs in both wet and dry forests below 1000 m. The nest galleries are diffuse and irregular. Mature colonies are large containing up to thousands of workers. Workers collect small arthropods and will recruit to sugar baits.

#### Distribution.

*Pheidole flavens* is among the most widespread and abundant species of its genus in the New World, although this range might be representative of multiple cryptic species. As currently conceived, however, we consider *Pheidole flavens* native from southern Mexico east through the Caribbean and south to Uruguay and northern Argentina. It is difficult to know whether the disjunction separating the western and eastern regions of South America is accurate or a sampling artifact. The Florida population is believed to have derived from an accidental introduction by commerce (Deyrup et al. 2000; Wilson 2003).

#### Risk statement.

*Pheidole flavens* (or at least it’s very close relatives) are easily transported long distances, and are known to hitchhike with fresh plant material (Wilson 2003). However, the species is not known to cause significant impact to agricultural systems or native ecosystems, and is not considered a house pest (Hedges 1998; Klotz et al. 1995).

### *Pheidole flavens*-complex

Fig. 88e

The *Pheidole flavens*-complex is defined here to include *Pheidole flavens* Roger, *Pheidole moerens* Wheeler, *Pheidole navigans* Forel, and their respective junior synonyms. A clear understanding of the phylogenetic relationship among the aforementioned taxa that are invading regions beyond the Neotropics remains a challenge for future studies (Sarnat et al. 2014).

**Diagnosis among introduced *Pheidole*.** Color variable. **Major** Head subquadrate (Fig. 7). Longitudinal carinae extend from anterior frons margin a variable distance beyond frontal carinae, but never reach posterior head margin (Fig. 25). Rugae of posterolateral lobes variable from mostly absent, to predominantly longitudinal, to distinctly reticulated. Posterior head margin always free of distinct rugae (Fig. 25) or rugoreticulum (Fig. 27). Microsculpture of posterolateral lobes variable from glossy to moderately punctate. Hypostoma with stout median and submedian teeth (Fig. 19). Promesonotal dorsum usually with distinct transverse striae (Fig. 21), but sometimes lacking distinct striae. Promesonotum in profile forming a single dome (Fig. 4), lacking a distinct mound or prominence on the posterior slope. Promesonotum not strongly transverse with strongly projecting sides in dorsal view (Fig. 29). Postpetiole not swollen relative to petiole (Fig. 3). Postpetiole relatively narrow in dorsal view; distinctly less than 2× petiolar width. Gaster with entire first tergite glossy (Fig. 32). **Minor** Head covered in punctate microsculpture, giving it a dull appearance. Posterior portion of head lacking many short to medium length segments of striae distinctly interlaced among punctate ground sculpture (Fig. 60). Antennal scapes often, but not always, surpass posterior head margin; if they do it is usually by a distance less than eye length. Antennal scapes with standing hairs present (Fig. 56). Promesonotum in profile forming a single dome (Fig. 42), lacking a distinct mound or prominence on the posterior slope. Hairs on mesosoma fine and flexuous, not arranged in pairs (Fig. 54). Pronotal humeri not angular. Postpetiole not swollen relative to petiole (Fig. 3). Postpetiole relatively narrow (Fig. 30); distinctly less than 2× petiolar width in dorsal view. Gaster with entire first tergite glossy (Fig. 32).

**Identification, taxonomy and systematics.** Members of the *Pheidole flavens*-complex are small species ranging from yellowish to dark reddish brown. The head and mesosoma of the minor workers are covered by densely punctate ground sculpture. The head of the major worker tends to be shinier with the posterior margin always free of sculpture. Among the species treated here, those of the *flavens* complex are most easily confused with those of the closely related and often sympatric *Pheidole punctatissima* clade (*Pheidole anastasii*, *Pheidole bilimeki*, *Pheidole punctatissima*). The postpetiole is narrower in the *Pheidole flavens* complex (Fig. 30, major; Fig. 61, minor) relative to those of the *Pheidole punctatissima* clade (Fig. 31, major; Fig. 62, minor). The gaster is completely glossy in both subcastes of the *Pheidole flavens* complex (Fig. 32), while at least the basal portion of the first gastral tergite is matte in those of the *Pheidole punctatissima* clade. In the *Pheidole flavens* complex the minors have finer hairs of variable lengths on the mesosoma (Fig. 54) and the antennal scapes have many erect to suberect hairs (Fig. 56). In contrast the *Pheidole punctatissima* clade have thicker mesosoma hairs of equal length (Fig. 53) and lack erect antennal scape hairs (Fig. 55). The minors of the *Pheidole flavens* complex are very difficult to distinguish from those of *Pheidole parva*. They can be separated by the lack of interlacing striae on the posterior head margin (Fig. 60, *Pheidole flavens* complex *vs.* Fig. 59, *Pheidole parva*). See key for additional characters.

The *Pheidole flavens* group as defined by Wilson (2003) is now known to be polyphyletic (Moreau 2008), and unpublished data analyzed by the authors suggests that the *Pheidole flavens* complex as defined by Wilson also lacks monophyly. *Pheidole exigua* is morphologically quite similar to the aforementioned taxa, and future attempts to define the *flavens* complex clade should include it in analyses, along with *Pheidole glomericeps* and possibly other species not initially considered by Wilson.

The most recent phylogeny of *Pheidole* includes eight taxa that form a well-supported *Pheidole flavens* clade (Economo et al. 2015). The clade consists of taxa that have been determined by various ant taxonomists as *Pheidole moerens*, *Pheidole flavens*, *Pheidole glomericeps*; several morphospecies including *Pheidole* sp. JTL-177 and a *Pheidole flavens*-complex taxon recently established on Vanuatu (Sarnat et al. 2014); and also the species we refer to as *Pheidole navigans* Forel. Although these taxa represent only a fragment of the diversity attributed to the *Pheidole flavens* complex, the analysis demonstrates the taxonomic confusion of the clade. For example, the *Pheidole moerens* sample from the Dominican Republic is most closely related to the taxa recently discovered on Vanuatu in the South Pacific. There is strong support for these taxa being more closely related to *Pheidole* JTL-177 (Venezuela) and two taxa from Central America determined as *Pheidole flavens* (collection codes PSW16014 and JTL4928) then to *Pheidole navigans* from Alabama (collection code PSW15833) and Venezuela (collection code PSW16167). The Alabama and Venezuela *Pheidole navigans* are actually most closely related to a taxon determined as *Pheidole glomericeps* (collection code Wa-D-01-2-16).

### Pheidole indica

Taxon classificationAnimaliaHymenopteraFormicidae

Mayr

Figs 73 , 78 , 88f

Pheidole *indica*. *Pheidole indica*Mayr 1879: 679 (s.w.q.) INDIA, Calcutta [NHMW, paralectotype s.w., examined]. Forel 1902b: 199 (m.); Imai et al. 1984: 6 (k.). Lectotype designated Eguchi 2004b: 199 (s.).
Pheidole Note. Material of the unavailable name Pheidole javana r. jubilans var. formosaeForel 1912: 60 referred to *Pheidole indica*: Eguchi 2004b: 199.
Pheidole *striativentris*. *Pheidole striativentris*Mayr 1879: 678 (s.) INDIA: Calcutta. Forel 1902b: 195 (w.q.). Junior synonym of *indica*: Eguchi 2004b: 199.
Pheidole *teneriffana*. *Pheidole teneriffana*Forel 1893b: 465 (s.w.) SPAIN, Canary Is. (s.) Laguna, Tenerife (M. Medina); (q.) Las Palmas, Canarías (Cabrera y Díaz). [Also described as new by Forel 1894a: 160.] Queen described: Santschi 1908: 521. Male described: Gómez and Espadaler 2006: 229. **n. syn.**
Pheidole *voeltzkowii*. *Pheidole voeltzkowii*Forel 1894b: 227 (s.w.m.) MADAGASCAR. Queen described: Forel 1897: 207. Junior synonym of *teneriffana*: Fischer and Fisher 2013: 340. **n. syn.**
Pheidole *himalayana*. Pheidole indica r. himalayanaForel 1902b: 185 (s.), 199 (w.) INDIA. [Also described as new by Forel 1902a: 546.] Raised to species: Bingham 1903: 265. Subspecies of *indica*: Emery 1921: 91; Menozzi 1939: 298; Pisarski 1967: 385. Junior synonym of *indica*: Eguchi 2004b: 198.
Pheidole *rotschana*. Pheidole indica r. rotschanaForel 1902b: 185 (s.), 199 (w.m.) INDIA: Poona, Orissa, Trevandrum and Thana. Lectotype designated Eguchi 2004b: 199 (s.) INDIA: Poona. Imai et al. 1984: 6 (k.). [Also described as new by Forel 1902a: 546.] Raised to species: Bingham 1903: 264. Subspecies of *indica*: Forel 1909b: 394; Forel 1911a: 222. Junior synonym of *indica*: Eguchi 2004b: 199.
Pheidole *taina*. Pheidole teneriffana subsp. tainaAguayo 1932: 219 (s.) CUBA, Holguín, viii.1930 (C.G. Aguayo). Junior synonym of *teneriffana*: Wilson 2003: 640. See also: Baroni Urbani 1968: 438; Snelling, R.R. 1992: 121. **n. syn.**

#### Diagnosis among introduced *Pheidole*.

Light to dark reddish brown. **Major** HW 1.32–1.74, HL 1.31–1.76, SL 0.73–0.91, CI 94–117, SI 47–62 (n=22). Head subquadrate (Fig. 7); rugoreticulate on posterolateral lobes and laterad of frontal carinae (Fig. 13a), but frons dominated by long, well-organized and parallel longitudinal rugae (Fig. 13b). Frontal carinae extend 3/4 distance of head before terminating (Fig. 15). Antennal scrobes indistinct to moderately impressed, but frontal carinae always forming a border capable of accepting the antennal scape (Fig. 13c). Hypostoma with weakly produced median tooth and submedian teeth. Promesonotum in profile with two convexities (Fig. 5), the large anterior dome in addition to a distinct mound or prominence on the posterior slope. Postpetiole not swollen relative to petiole (Fig. 3). **Minor** HW 0.50–0.65, HL 0.60–0.74, SL 0.64–0.81, CI 72–90, SI 120–149 (n=20). Head predominantly glossy (Fig. 36), lacking punctation and or rugae above eye level. Posterior head margin weakly convex to flat in full-face view (Fig. 45). Antennal scapes long (e.g. Fig. 39), but not surpassing the posterior head margin by more than 2× eye length. Promesonotum in profile with two convexities, the large anterior dome (Fig. 43a) in addition to a distinct prominence on the posterior slope (Fig. 43b). Promesonotal prominence relatively convex (Fig. 50a). Metanotal depression relatively shallow (Fig. 50b). Petiole and postpetiole glossy to very weakly sculptured laterally (Fig. 48). Postpetiole not swollen relative to petiole (Fig. 3).

**Figure 73.**
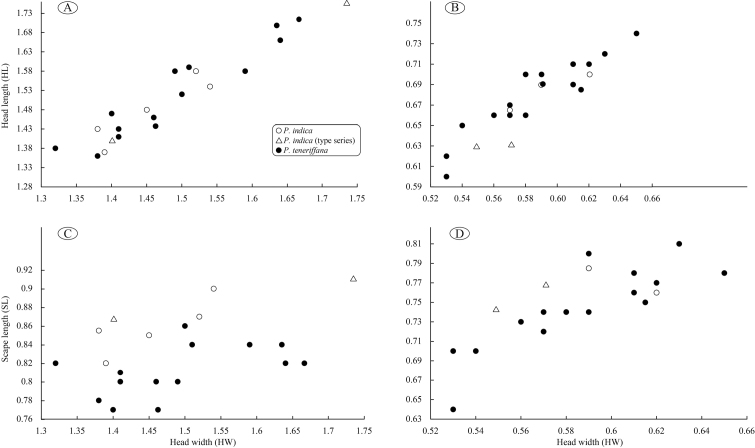
Relative morphometric measurements of *Pheidole indica* paralectotypes, specimens previously determined as *Pheidole indica*, and specimens previously determined as *Pheidole teneriffana*. All values are in mm. **A** Head length *vs.* head width, major workers. **B** Head length *vs.* head width, minor workers. **C** Scape length *vs.* head width, major workers **D** Scape length *vs.* head width, minor workers.

#### Identification, taxonomy and systematics.

*Pheidole indica* is a medium to large reddish brown species with relatively long limbs. It belongs to the *Pheidole fervens* clade along with its Australasian congeners *Pheidole cariniceps*, *Pheidole fervens*, *Pheidole hospes*, *Pheidole impressiceps*, and *Pheidole oceanica* (Economo et al. 2015, unpublished data). The major and minor workers are distinguished from those of *Pheidole megacephala* by the lack of a swollen postpetiole (Fig. 3). The majors are also easily separated from those of *Pheidole megacephala* by the strongly sculptured head (Fig. 13). The minors can be confused with those of *Pheidole megacephala* because both have glossy heads. However, the minors of *Pheidole fervens* can be separated from those of *Pheidole megacephala* by the relatively longer antennal scapes (Fig. 39 *vs.* Fig. 40) and the presence of a promesonotal prominence (Fig. 43 *vs.* Fig. 42). *Pheidole indica* is broadly sympatric with *Pheidole noda* and *Pheidole fervens*. It is easily separated from the former by the lack of a swollen postpetiole (Fig. 3 *vs.* Fig. 2). Separation from *Pheidole fervens* is quite difficult, and readers are referred to corresponding section under that species for distinguishing characters. Readers are referred to Eguchi (2004b; 2008) for characters used to separate *Pheidole indica* and *Pheidole fervens* from their Asian congeners.

*Pheidole indica* was originally described from India. Eguchi (2004b) synonymized several other Asian congeners under *Pheidole indica* and discussed taxonomic differences used to distinguish it from *Pheidole fervens* and other morphologically similar species. We synonymize *Pheidole teneriffana* under *Pheidole indica* based on morphological analysis of the type specimens and genetic analysis of previously determined specimens (unpublished data). Forel, in his original description of *Pheidole teneriffana*, noted the similarity between it and *Pheidole striativentris* [= *indica*].

The biogeographical origin of *Pheidole teneriffana* has been a minor mystery of the past century, as revealed by the recent review of the species by Wetterer (2011). There appeared to be general consensus that *Pheidole teneriffana* was native to at least some portion of North Africa, Arabia, the Middle East or the Mediterranean. Santschi (1918), suggested the upper Nile area (South Sudan). Wilson (2003) suggested North Africa and potentially the Canary Islands. Collingwood et al. (2004) suggested it was native throughout northern Africa and observed it to be, “spreading over a wide front in the Middle East, Arabia and the Mediterranean countries.” Wetterer (2011) found the distribution of *Pheidole teneriffana* enigmatic, “Curiously, most Old World records of *Pheidole teneriffana* are subtropical, but all New World records are tropical, except one from California…If *Pheidole teneriffana* is truly native across North Africa, it is remarkable how few records I found from any North African country other than Egypt.”

#### Biology.

In Asia *Pheidole indica* is known to nest in soil or under stones in open and dry habitats (Eguchi 2004b). It is among the most widespread *Pheidole* species in Asia. In the Caribbean Wetterer (2011) found *Pheidole indica* [as *Pheidole teneriffana*] almost exclusively on beaches and at highly disturbed urban sites, particularly in waterfront areas. In northern Africa, Santschi (1908) noted the tramp-like distribution of what he treated as *Pheidole teneriffana*, “This species, described by Forel on samples from the Canary Islands, was sent to me from Cairo. I discovered it most recently in Sousse [Tunisia], in the park, near the port. As it does not exist in the interior, I think it is one species cosmopolitan tendencies. It nests in the ground and under stones.” Santschi (1934) later reported the species from Alexandria, Egypt, and noted that *Pheidole teneriffana* was rarely reported far from seaports. Collingwood et al. (1997) reported that in the United Arab Emirates, *Pheidole indica* [as *Pheidole teneriffana*] was populous in irrigated gardens and along the coast where it appeared to be spreading rapidly, possibly to the detriment of local species. The species has also been reported from urban areas of the Balearic Islands where it is common in the gardens and trees and on sidewalks near the harbor (Gómez and Espadaler 2006). Fischer and Fisher (2013) reported *Pheidole indica* [as *Pheidole teneriffana*] from the Malagasy region. It was collected on the Comoros, Mauritius, the Seychelles, and from coastal towns in Madagascar, usually from under stones, ground nests, or foraging on the ground or lower vegetation in urban or garden habitats at elevations below 300 m. It was also found on Mayotte in native littoral and secondary forest below 10 m.

Perhaps the most detailed study of *Pheidole indica* in the New World comes from the account of Martínez (1992) who reported a vigorous population, represented by a putatively single polydomous colony spanning several hectares, that was discovered in Long Beach, California in 1989. Martínez (1992) reportedly observed 23 inseminated queens from a single colony that was changing nest sites (although no details are given for how he knew the queens were inseminated). He described the colony nests as low mounds on the soil, along curbs or sidewalks, at the edges of lawns, in cracks in pavement, and at the bases of trees. New colonies were started by budding. Workers foraged night and day unless temperature exceeded 26 °C, taking seeds and scavenging dead or dying insects. They were observed feeding on sweet or greasy foods, but were not seen tending aphids. Martínez (1992) observed the species attacking native ants, including *Pogonomyrmex californicus* (Buckley). More remarkably, he reportedly observed *Pheidole indica* destroying colonies and taking over nest sites of *Linepithema humile*. Despite the purported success of these battles, *Pheidole indica* must have lost the larger war against *Linepithema humile*, as the eventual extirpation of the Californian population was attributed to the Argentine ant (Gulmahamad 1999).

#### Distribution.

We treat all occurrence records from the regions of Indomalaya west of the Korean Peninsula as native. The Korean and Japanese populations are considered introduced (Choi and Bang 1993; Choi et al. 1993a; Choi et al. 1993b; Terayama 1992), and additional portions of the range in Asia might also have resulted from anthropogenic transport. *Pheidole indica* has been introduced to scattered localities across the globe, although the vast majority of these records were attributed to its junior synonym, *Pheidole teneriffana*. Introduced populations have been reported from the Mediterranean, northern Africa, the Malagasy region, Western Australia, Peru, the Caribbean, and southern California.

#### Risk statement.

*Pheidole indica* is not considered to be a major pest to either agriculture or native ecosystems. Although the species is tolerant of disturbed and urban areas, we found no reports of it infesting structures. Few studies have measured the effect of *Pheidole indica* on ecosystem health, but we predict that it could negatively impact native arthropods. The species is continuing to spread across the globe and further studies are required to test its ecological and agricultural impact outside its native range.

### Pheidole megacephala

Taxon classificationAnimaliaHymenopteraFormicidae

(Fabricius)

Figs 79 , 88g

Pheidole *edax*. *Formica edax*Forskål 1775: 84 (w.) EGYPT. Junior synonym of *megacephala*: Emery 1892: 160; Dalla Torre 1892: 90. [If synonymy correct then *edax* is the senior name; however, under Art. 23.9 of ICZN (1999) *edax* is a *nomen oblitum*.]
Pheidole ***megacephala***. *Formica megacephala* Fabricius, 1793: 361 (s.) MAURITIUS ‘Ile de France’ [presumed lost]. Neotype (s.) designated: MAURITIUS, Camizard Mt., Bambous, 20.3328 S, 57.723 E, 375 m, rainforest, ex rotten log, collection code BLF12051, 27.v.2005 (*B.L. Fisher et al.*) (CASC: CASENT0104990): (Fischer and Fisher 2013): 332. Latreille 1802: 232 (q.); Mayr 1861: 70 (s.w.q.m.); Wheeler, G.C. & Wheeler, J. 1953: 75 (l.). Combination in *Pheidole*: Roger 1863b: 30. [*Pheidole megalocephala*Schulz 1906: 155; unjustified emendation.] Current subspecies: nominal plus *costauriensis*, *duplex*, *ilgi*, *impressifrons*, *melancholica*, *nkomoana*, *rotundata*, *speculifrons*, *talpa*.
Pheidole Note: *Pheidole megacephala* Smith, F. 1860: 112 is a junior synonym of *Carebara diversus* (Jerdon): Emery 1893: 206.
Pheidole *trinodis*. *Myrmica trinodis*Losana 1834: 327, pl. 36, fig. 6 (w.) ITALY, Piedmont. Junior synonym of *megacephala*: Roger 1863b: 30.
Pheidole *pusilla*. *Oecophthora pusilla*Heer 1852: 15, pl. 1, figs. 1-4 (s.w.q.m.) PORTUGAL, Madeira I. Combination in *Pheidole*: Smith, F. 1858: 173. Subspecies of *megacephala*: Emery 1915b: 235. Senior synonym of *janus*: Mayr 1886: 360; of *laevigata* Smith: Roger 1859: 259; Emery 1915b: 235; of *laevigata* Mayr: Mayr 1870b: 981 (footnote). Junior synonym of *megacephala*: Wheeler, W.M. 1922b: 812.
Pheidole *laevigata*. *Myrmica* (?) *laevigata*Smith 1855: 130, pl. 9, figs. 7, 8 (w.) GREAT BRITAIN, Battersea. Junior synonym of *Pheidole pusilla*: Roger 1859: 259; of *Pheidole pallidula*: Smith 1858: 282; of *Pheidole megacephala*: Roger 1863: 30; of *Pheidole pusilla*: Emery 1915: 235.
Pheidole *agilis*. *Myrmica agilis* Smith, F. 1857: 71 (w.) MALAYSIA, Malacca. Combination in *Pheidole*: Donisthorpe 1932: 449. Junior synonym of *megacephala*: Eguchi 2008: 56.
Pheidole *janus*. *Pheidole janus* Smith, F. 1858: 175, pl. 9, figs. 13-17 (s.w.) SRI LANKA. Junior synonym of *pusilla*: Mayr 1886: 360.
Pheidole *testacea*. *Atta testacea* Smith, F. 1858: 168 (s.w.) BRAZIL. Combination in *Pheidole*: Mayr 1886: 360. Junior synonym of *megacephala*: Brown 1981: 530.
Pheidole *perniciosa*. *Oecophthora perniciosa*Gerstäcker 1859: 263 (w.) MOZAMBIQUE. [Also described as new by Gerstäcker 1862: 516.] Combination in *Pheidole*: Roger 1863b: 31. Junior synonym of *megacephala*: Emery 1915b: 235.
Pheidole *suspiciosa*. *Myrmica suspiciosa* Smith, F. 1859: 148 (w.) INDONESIA, Aru I. (A.R. Wallace). Junior synonym of *megacephala*: Donisthorpe 1932: 455.
Pheidole *laevigata*. *Pheidole laevigata*Mayr 1862: 747 (s.) BRAZIL. Unresolved junior secondary homonym of *Pheidole laevigata* Smith, F. Junior synonym of *Pheidole pusilla*: Mayr 1870: 981 (footnote).
Pheidole *scabrior*. Pheidole megacephala var. scabriorForel 1891: 178 (s.w.) MADAGASCAR. Junior synonym of *megacephala*: Fischer and Fisher 2013: 333.
Pheidole *picata*. Pheidole megacephala var. picataForel 1891: 178 (s.w.) MADAGASCAR. Subspecies of *megacephala*: Forel 1895: 49; of *punctulata*: Forel 1897: 186; Forel 1905: 163; Santschi 1910: 370. Raised to species: Emery 1915b: 245; Wheeler, W.M. 1922a: 1019. Junior synonym of *megacephala*: Fischer and Fisher 2013: 333.
Pheidole *gietleni*. Pheidole punctulata r. gietleniForel 1905b: 164 (s.w.) MADAGASCAR. Subspecies of *picata*: Emery 1915b: 245. Junior synonym of *megacephala*: Fischer and Fisher 2013: 333.
Pheidole *bernhardae*. Pheidole picata var. bernhardaeEmery 1915b: 245 (s.w.) MADAGASCAR. [First available use of Pheidole punctulata r. spinosa var. bernhardae Forel, 1905: 164; unavailable name.] Junior synonym of *megacephala*: Fischer and Fisher 2013: 333.

#### Diagnosis among introduced *Pheidole*.

Light brown to dark brown. **Major** HW 1.10–1.54, HL 1.04–1.59, SL 0.59–0.76, CI 97–106, SI 47–58 (n=19, Fischer and Fisher 2013). Head heart-shaped (Fig. 6); posterior 1/3 of dorsal surface smooth, glossy and entirely lacking rugoreticulate sculpture. Hypostoma lacking distinct median and submedian teeth. Promesonotum in profile forming a single dome (Fig. 4), lacking a distinct mound or prominence on the posterior slope. Postpetiole with a posterodorsal (Fig. 1a) and anteroventral (Fig. 1b) bulge. **Minor** HW 0.50–0.61, HL 0.57–0.68, SL 0.61–0.72, CI 86–92, SI 114–122 (n=20, Fischer and Fisher 2013). Head predominantly glossy (Fig. 36), lacking punctation and or rugae above eye level. Antennal scapes surpass posterior head margin by approximately same length as eye (Fig. 40). Promesonotum in profile forming a single dome (Fig. 42), lacking a distinct mound or prominence on the posterior slope. Postpetiole with a posterodorsal (Fig. 1a) and anteroventral (Fig. 1b) bulge.

#### Identification, taxonomy and systematics.

*Pheidole megacephala* is a medium sized species of variable color that is most easily recognized outside of its native range by the heart-shaped head and bulging postpetiole. It belongs to a diverse and taxonomically confusing clade of morphologically similar taxa centered in the Afrotropical and Malagasy regions. Both major and minor workers are distinguished from all other introduced *Pheidole* by the swollen shape of the postpetiole (Fig. 1). *Pheidole noda* also has a swollen postpetiole, but whereas the postpetiole of *Pheidole megacephala* is characterized by a posterodorsal and anteroventral bulge, that of *Pheidole noda* is formed as a high dorsally bulging dome that is tallest at its midpoint.

*Pheidole megacephala* has often been confused for *Pheidole pallidula* Nylander in Europe, especially in the Mediterranean region. The introduced populations of *Pheidole megacephala* can be distinguished from *Pheidole pallidula* by the following characters. For both major and minor workers the postpetiole of *Pheidole megacephala* has a posterodorsal (Fig. 1a) and anteroventral (Fig. 1b) bulge, while that of *Pallidula* is not swollen relative to petiole (Fig. 3). The propodeal spines of both subcastes are distinct in *Pheidole megacephala* but are strongly reduced in *Pheidole pallidula*. Additionally, the major worker of *Pheidole megacephala* has a heart shaped head that broadens significantly posterior to eye-level (Fig. 6) while the head of *Pallidula* is more rectangular (more approximate to Fig. 7).

Accurate identification within the Afrotropics is more problematic. While for Madagascar previously described subspecies have been synonymized with *Pheidole megacephala* (Fischer and Fisher 2013), the taxonomy of the *megacephala* group in Africa remains rather chaotic with a number of unrevised subspecies, most of which remain insufficiently characterized. In a taxonomic overview of the group, Emery (1915) studied type and non-type material of *Pheidole megacephala*-related species, yet for several subspecies he was not able to define clear species limits from the multitude of different, yet highly similar, phenotypes. We suspect that some of those names are probably due to intraspecific variation within *Pheidole megacephala* and *Pheidole punctulata* Mayr. Other, morphologically unique taxa like *Pheidole megacephala nkomoana* Forel are clearly valid biological species. However, without a comprehensive taxonomic treatment supported by a robust phylogeny, the following species characterizations may be subject to future taxonomic changes.

Within the *megacephala* group, minor workers are difficult to separate morphologically and thus have only limited use for species identification, but the majors tend to be more distinct in their morphologies and can be separated by differences in head and body shape and sculpture, and in size and pilosity, although the limits are often unclear and characters are sometimes distributed along a continuum rather than being separated into distinct, clear-cut states.

Major workers of *Pheidole megacephala melancholica* Santschi are characterized by presence of weak punctures on the majority of the head, including the sides in lateral view, promesonotum with punctures and irregular transverse rugulae, and moderately abundant short and stout standing hairs on head and body, whereas major workers of *Pheidole megacephala* entirely lack punctures on the posterior 1/3 of the head, have a mostly smooth and glossy promesonotum, and often possess longer, more flexuous standing hairs, which often branch at the tips. *Pheidole megacephala nkomoana* majors are characterized by a weakly defined antennal scrobe and relatively long frontal carinae that reach about ¾ towards the posterior head margin, two well-defined submedian hypostomal teeth, a weak prominence on the promesonotal dome, and very long, flexuous standing hairs on the dorsal promesonotum. Also the spines tend to be shorter than in *Pheidole megacephala*, in length almost equal to the diameter of the propodeal spiracle. Both subspecies have been described from and collected in western African forests. Another closely related species to *Pheidole megacephala* is *Pheidole punctulata*. It is very widespread in sub-Saharan Africa and usually found in dry forests and grassland habitats. Morphologically close to *Pheidole megacephala*, its major workers can be distinguished by their often enlarged and strongly heart-shaped heads, the presence of a softly or superficially punctuated sculpture on parts of the head dorsum, promesonotum, postpetiole and gaster, and relatively uniform, short and stout, erect hairs covering the body. Minor workers tend to be slightly larger and more robust than in *megacephala*, often with a few oblique carinae present between the eyes and the mandibles and reaching the posterior eye level, the hairs similar as in major workers and usually more abundant than in *Pheidole megacephala*.

Morphologically very similar to *Pheidole punctulata* are *Pheidole megacephala ilgi* Forel, *megacephala impressifrons* Wasmann, and *megacephala rotundata* Forel. Like *Pheidole punctulata*, they are usually found in drier forest and grassland habitats and their workers seem to be highly polymorphic, which means that in addition to normal major workers, colonies are capable of producing so-called supermajors. These supermajors possess a very strongly heart-shaped head, which can be disproportionately big compared to the size of the mandibles and the rest of their bodies. As Emery (1915) stated for *Pheidole megacephala rotundata*, on first glace they look quite distinct from *Pheidole punctulata*, but at closer examination of series with different major worker sizes it seems impossible to define species limits. From our own observations it seems likely that these subspecies are a result of sampling bias and phenotypic variation within *Pheidole punctulata*, rather than historic speciation events (Fischer et al., in preparation). Incomplete sampling can also be a problem when only smaller major and minor workers are collected, which are often very similar to those of *Pheidole megacephala*, with very similar head sculpture and general morphology.

In the Malagasy region, *Pheidole megacephala* can be confused with three other species: *Pheidole punctulata spinosa* Forel, which, on average, has longer spines, a slightly higher propodeum and a more extensively smooth and glossy posterior portion of the head in the larger major workers. *Pheidole megatron*, which was described from the Comoros and is possibly present in the Northwest of Madagascar as well, is characterized by major workers with a less heart-shaped, and slightly more rectangular head shape, and sometimes sculpture and rugulae present on the posterior head portion (see Fischer and Fisher 2013). Finally, *Pheidole decepticon*, described from Mayotte and distributed over several of the smaller Southwest Indian ocean islands, is characterized by possessing a denser, more prominent and longer pilosity as well as slightly smaller, less rounded ventral bulges on the postpetiole in both minor and major workers (see Fischer and Fisher 2013). It is however possible that *Pheidole decepticon* is a geographic variation of and conspecific with *Pheidole punctulata spinosa*.

#### Biology.

*Pheidole megacephala* is listed among the top five invasive ants (Lowe et al. 2000). Although this species prefers humid and disturbed habitats where it is usually found in very high abundances (Burwell et al. 2012; Hoffmann et al. 1999; Wilson 2003), it can generally be found in a large variety of landscapes, from coastal habitats to human settlements and plantations in lower elevations, degraded dry forest, to mid-elevation rainforest or even montane forest – in Papua New Guinea up to 2150 meters altitude (Fischer and Fisher 2013). The distribution range and activity of *Pheidole megacephala* appears to be somewhat limited by susceptibility to desiccation and higher temperatures. Thus, colonies are often found in more humid microhabitats, and workers tend to forage inside the leaf-litter and at night, or even build covered trails (Greenslade 1972, personal observations). However, some studies reported that on smaller islands or after successful introduction in a new area, *Pheidole megacephala* expanded its range and invaded into the forest interiors where it attacked and displaced other introduced and natively occurring ant species (Burwell et al. 2012; Hoffmann 1998). In a citrus orchard in Tanzania for example, *Pheidole megacephala* was able to partly displace highly territorial and competitive *Oecophylla* weaver ants (Seguni et al. 2011). *Pheidole megacephala* is an especially common and abundant nuisance and pest on islands, which are generally more strongly impacted by invasions of alien species.

Part of the success of *Pheidole megacephala* as a pantropic pest species is its generalist behavior. Like many other *Pheidole* species its diet is broadly omnivorous with a large proportion of its food probably acquired by scavenging on the ground. *Pheidole megacephala* is also a good predator with an efficient nest mate recruitment that enables the species to dominate baits and to retrieve prey too large for single workers to carry (Dejean et al. 2008; Dejean et al. 2007). Devastating effects on the abundance and diversity of native invertebrates, in northern Australia for example, are well documented (Hoffmann 1998; Hoffmann et al. 1999; Hoffmann and Parr 2008). *Pheidole megacephala* has also been documented to negatively impact agricultural systems. Workers tend plant and crop-damaging scale insects for honeydew (Campbell 1994; Gaigher et al. 2011; González Hernández et al. 1999; Greenslade 1972; Petty and Tustin 1993; Reimer et al. 1993), protect plants with extrafloral nectaries from phytophagous insects and possibly collect seeds (Hoffmann 1998). A recent study experimentally evaluating the performance in interference competition found that *Pheidole megacephala* ranked lowest among seven of the world’s worst most destructive invasive ant species (Bertelsmeier et al. 2015). The authors, citing Dejean et al. (2008) suggested that *Pheidole megacephala* does not dominate invaded ant communities through direct physical interactions (interference competition) but by raiding their colonies.

Nesting sites are variable and can occur in any crack and crevice that is large enough for them to enter, including soil, inside rotting logs, under rocks, in houses or in tree bark. As in several other invasive ant species, colonies are polygynous, and dependently founded via budding, with nests in large areas often forming supercolonies (Hoffmann 1998) that aggressively fight other ants or outcompete them by depleting their prey and other resources (Dejean et al. 2008; Fournier et al. 2009; Hoffmann et al. 1999; Vanderwoude et al. 2000).

#### Distribution.

*Pheidole megacephala* is a cosmopolitan species that has established across the globe as a household and agricultural pest throughout the tropics. Wetterer (2012) provided a detailed review of the worldwide spread of *Pheidole megacephala*, and cites Wheeler’s statement (Wheeler 1922a) that it is most likely of Afrotropical or Malagasy origin, the only two regions with a diversity of related species (“subspecies and varieties”). Theoretically it is possible that a common ancestor of *Pheidole megacephala* and the Malagasy endemics *Pheidole punctulata spinosa*, *Pheidole megatron* and *Pheidole decepticon* arrived on the islands in prehistoric times, diversified there, and that *Pheidole megacephala* was later transported to all other regions including Africa only after the arrival of humans. But the distribution of *Pheidole megacephala* on Madagascar strongly resembles the distributions of other invasive species on the island – e.g. those of *Monomorium floricola*, *Monomorium pharaonis*, *Tapinoma melanocephalum*, *Technomyrmex albipes*, *Trichomyrmex destructor*. While *Pheidole punctulata spinosa* has established a broad distribution range across the island’s variable habitats and elevations, *Pheidole megacephala*, like the other invasives, is found mostly along the coast, in low elevation and disturbed habitats or near human settlements.

Similar to Wheeler’s observation, our argument for the “out-of-Africa” hypothesis is an overall much higher complexity in different morphotypes and species-level diversity in African *megacephala* group taxa and the presence of both, very closely, but also more distantly related taxa (e.g *Pheidole aurivillii* Mayr). For these reasons and for the purposes of this study, we consider all records from Africa to represent the native range of *Pheidole megacephala*. However, a further resolution will require a comprehensive phylogeographic study of the species and its allied taxa, especially from the poorly studied and sampled African region.

Populations of *Pheidole megacephala* recorded from the southwestern extent of the Arabian Peninsula are treated as native as this region is commonly considered as belonging to the Afrotropics. However, recent studies on generic distributions of global ant diversity that find little support for including any portion of the Arabian Peninsula in the Afrotropics (unpublished data). Until robust phylogeographic data is available for *Pheidole megacephala*, this decision must be considered tentative and open to future revision.

We do agree with Wetterer’s (2012) conclusions that records of *Pheidole megacephala* from Mediterranean Europe northward are either temporary indoor records or misidentifications of *Pheidole pallidula*. Outside of Africa, the Malagasy region and the range of *Pheidole pallidula* (western Palearctic), *Pheidole megacephala* is easily recognized as it does not co-occur with species of similar morphology. We therefore consider all records reviewed from outside the aforementioned regions as confirmed unless otherwise stated.

#### Dubious records.

The following records are considered dubious mostly because there is reason to believe they represent misidentifications of *Pheidole pallidula*. However, it is possible that some of the following literature records were based on accurate identifications, but that *Pheidole megacephala* was since extirpated from the referenced localities. This latter possibility is plausible especially for the Mediterranean region where *Linepithema humile* has established a stronghold. For example, (Heer 1852) described *Oecophthora pusilla* (=*Pheidole megacephala*) as ubiquitous on the island of Madeira, “In the town of Funchal there is probably not a single house that does not harbor millions of the tiny creatures…” Less than a century later Wheeler (Wheeler 1927b) reported, “Now it is an interesting fact that the Argentine ant, soon after its arrival in Madeira, completely replaced the *Pheidole* as a house ant.” Similar instances of well-established populations of introduced ant species becoming locally extirpated have been documented (Moreau et al. 2014; Wetterer 2006).

Algeria: The material referred to by André (1883) *Pheidole megacephala* is distinguished by that author from *Pheidole pallidula* only by the difference in size of the propodeal spine, and was otherwise observed to be identical. Considering the other characters separating these two species discussed earlier, we tentatively consider this record to be a misidentification of *Pheidole pallidula*. Croatia: The material listed from this country (Petrov and Collingwood 1992; Petrov and Legakis 1996) is considered to refer to *Pheidole pallidula* according to Bračko (2006). Egypt: Egypt is the type locality of the *nomen oblitum* Formica edax Forskål. Emery (1892) wrote that *edax* is undoubtedly a small *Pheidole*, and possibly refers to *Pheidole megacephala*. Dalla Torre (1892) was also uncertain as to which species (or even genus) the name *edax* referred to. Given the uncertainty of these two authors, the occurrence of *Pheidole pallidula* in Egypt and the unconfirmed single literature record of Bakr et al. (2007), it is difficult to know when *Pheidole megacephala* was first reported from Egypt. France: Bignell (1901) reported the ant species listed in his study of Corsica were identified by Saunders, who is known to have confused *Pheidole pallidula* for *Pheidole megacephala*. As *Pheidole pallidula* was not listed in the publication, we consider the record to either be a misidentification of that species or from an extirpated population. Greece: The only primary references to an outdoor occurrence we could confirm are Collingwood (1993) and Borowiec and Salata (2012). The former authors reported *Pheidole megacephala* was found only once during their study of five Greek islands on the threshold of a small hotel in Pigadhia on Karpathos. The second study reported finding the species on a road in Crete. The record from Macedonia in (Karaman 2011) is from material identified by Petrov. We tentatively follow (Bračko 2006) as treating this as a misidentification of *Pheidole pallidula*. Italy: Piedmont is the type locality for *Myrmica trinodis* Losana which was synonymized with *megacephala* by Roger in 1863. Losana also lists a *Messor megacephala* Latrielle in the same publication. Latrielle never described any species by the name *megacephala*, however. Losana might have instead been referring to *Messor megacephala* Leech (= *Messor barbarous* Mayr). Regardless, the original description of *Myrmica trinodis* states that the species was collected from outdoor gardens. There is some reason to suspect this name might refer instead to *Pheidole pallidula*, as the only verifiable occurrences of *megacephala* in Italy since are for specimens collected from plant nurseries, greenhouses and cargo hangars used for holding imported plants, fruits and vegetables (Jucker et al. 2008; Limonta and Colombo 2003). Morocco: Saunders (1888) appears to be the only primary reference for *Pheidole megacephala* occurring in Morocco, but it is likely that the author was referring to misidentified material of *Pheidole pallidula* (Wetterer 2012). This view is further evidenced by Cagniant and Espadaler (1993) who were unable to find the species in their survey. Spain: We consider the following records from the Balearic Islands and Gibraltar to refer to *Pheidole pallidula* (Saunders 1888; Saunders 1904; Walker 1889). USA: The specimens reported in Fischer and Fisher (2013) from Arizona were from a quarantine collection intercepted from Florida, and there is no reason to believe the species has ever established in Arizona. Wetterer (2012) cited a specimen record of *Pheidole megacephala* from Catalina Island (California). If the identification proves accurate, it is the only known record from that island and the population has since been extirpated (perhaps by *Linepithema humile*). However, a population (CASENT0248690) has been discovered recently in southern California (Orange Co.). Although *Pheidole megacephala* is listed in the Missouri Ants web page (2015), we cannot verify the entry with any specimen or literature record.

#### Risk statement.

*Pheidole megacephala* is known as a major agricultural and ecological pest species (Williams 1994) and its widespread pantropic distribution and often very close association with humans make it a high-risk invasive species with a serious potential for ecological, agricultural and economic damage. In Ward et al. (2006) it has been the most intercepted exotic ant species (890 out of 4355 interception records between 1955 and 2005) arriving with trade products in New Zealand. Many aspects of its biology indicate that it is highly adaptable and thus able to survive outside of its preferred habitat, by finding suitable microhabitats for nesting and by killing or outcompeting native species. Although mutualistic relationships with scale insects and other crop pests are dominant in agricultural systems with introduced *Pheidole megacephala*, positive side-effects on plant fitness have been observed as well (Bach 1991).

### Pheidole navigans

Taxon classificationAnimaliaHymenopteraFormicidae

Forel stat. rev., stat. n.

Figs 80 , 88h

Pheidole *navigans*. Pheidole flavens r. navigansForel 1901a: 79 (s.w.) GERMANY (intercepted in quarantine from Veracruz, Mexico) [MHNG, examined photographs of CASENT0908269 (s.), CASENT0908270 (w). Junior synonym of *flavens*: Wilson 2003: 419. **stat. rev., stat. n.**
Pheidole *Pheidole moerens* (*nec* Forel): M.R. Smith 1967, Wojcik 1975, Glancey 1976, Naves 1985, Deyrup 1988, Deyrup 2000, Dash 2008, MacGown 2010, Guénard 2012. [We propose the preceding authors misapplied the name *Pheidole moerens* Forel to material considered here as referring to *Pheidole navigans* Forel. *Pheidole moerens* remains a valid name].

#### Diagnosis among introduced *Pheidole*.

Color reddish brown. **Major** HW 0.84–0.88, HL 0.88–0.91, SL 0.46–0.48, CI 95–99, SI 53–56 (n=4). Head subquadrate (Fig. 7). Longitudinal carinae of the frons extend to approximately an eye’s length distance from the posterior head margin (Fig. 25). Rugae of posterolateral lobes predominantly longitudinal. Posterior head margin always free of distinct rugae (Fig. 25) or rugoreticulum (Fig. 27). Microsculpture of posterolateral lobes glossy to weakly punctate. Antennal scrobe distinct and narrow, shallow but capable of receiving the entire antennal scape in repose (Fig. 71a); bordered by strong, unbroken frontal carina mesially (Fig. 71b); depression marked by a continuous smooth surface entirely (or nearly entirely) uninterrupted by rugulae. Hypostoma with stout median and submedian teeth. Promesonotal dorsum with distinct transverse striae (Fig. 21). Promesonotum in profile forming a single dome (Fig. 4), lacking a distinct mound or prominence on the posterior slope. Promesonotum not strongly transverse with strongly projecting sides in dorsal view (Fig. 29). Postpetiole not swollen relative to petiole (Fig. 3). Postpetiole relatively narrow in dorsal view; distinctly less than 2× petiolar width (Fig. 30). Gaster with entire first tergite glossy (Fig. 32). **Minor** HW 0.40–0.45, HL 0.45–0.50, SL 0.40–0.44, CI 86–92, SI 96–102 (n=8). Head covered in punctate microsculpture, giving it a dull appearance (Fig. 37). Antennal scapes reach or weakly surpass posterior head margin; if they do it is usually by a distance less than eye length. Antennal scapes with standing hairs present (Fig. 56). Promesonotum in profile forming a single dome (Fig. 42), lacking a distinct mound or prominence on the posterior slope. Hairs on mesosoma fine and flexuous, not arranged in pairs. Pronotal humeri not angular. Postpetiole not swollen relative to petiole (Fig. 3). Postpetiole relatively narrow (Fig. 30); distinctly less than 2× petiolar width in dorsal view. Gaster with entire first tergite glossy (Fig. 32).

#### Identification, taxonomy and systematics.

*Pheidole navigans* is a small, short-limbed, reddish brown species that belongs to the *Pheidole flavens* complex. See discussion under corresponding section of *Pheidole flavens* complex for how to distinguish this species from introduced *Pheidole* outside the complex. Within the complex, minor workers are impossible to distinguish based on known characters. Major workers can be separated from those of *Pheidole flavens* by the combination of predominantly longitudinal rugae on the posterolateral lobes, the more distinct and narrow antennal scrobe bordered mesially by strong, unbroken frontal carina, and the more continuously glossy scrobe depression.

Although the type locality of *Pheidole navigans* is Germany, the species was originally described by Forel from specimens intercepted during quarantine inspection of orchids originating from Veracruz, Mexico. We revive this name from synonymy and elevate it to species rank so that it can be applied to a putative species that has recently established in the southeastern United States and Hawaii. This ant has most often been referred to as *Pheidole moerens* since it was first reported from Alabama nearly fifty years ago by M.R. Smith (1967). However, the examination of type specimen photographs (MCZ-ENT00009137) suggests that these introduced populations are heterospecific with *Pheidole moerens* Wheeler.

Whether the introduced populations are actually conspecific with *Pheidole navigans* Forel will require a thorough revision of this taxonomically vexing species complex. Of all the type material we have examined, however, that of *Pheidole navigans* bears the closest resemblance in gross morphology. Thus we propose *Pheidole navigans* Forel be used in place of *Pheidole moerens* for referring to the aforementioned introduced populations. Future systematic study of this species should also examine Pheidole floridana subsp. aechmeae (currently synonymized under *Pheidole flavens*, but also recorded from Veracruz, Mexico) and Pheidole flavens var. mediorubra Santschi (described from Loreto, Argentina and currently treated as a synonym of *Pheidole alacris* Santschi).

The major workers of *Pheidole navigans* differ from those of *Pheidole moerens* in the following respects. They exhibit a distinct and narrow antennal scrobe capable of receiving the entire antennal scape in repose. The scrobe is bordered by a strong, unbroken frontal carina mesially, and the depression is marked by a continuous smooth surface entirely (or nearly entirely) uninterrupted by rugulae. The rugulae of the frons extend to approximately an eye’s length distance from the posterior head margin. The anterior portion of the promesonotum is crossed by long and distinct transverse striae.

The examined major workers of *Pheidole navigans* from Alabama (CASENT0106664) and Venezuela (CASENT0248831), along with those from Florida and Hawaii, and a specimen imaged from Paraguay (CASENT0178020), share a notably consistent morphology for being spread across such as wide range. The characteristics shared among these majors include the following. Frontal carinae strongly produced, forming the mesad border of a shallow but well-demarcated antennal scrobe capable of accommodating the entire scape in repose. Antennal scrobe weakly foveolate. Cephalic carinulae mostly longitudinal with very little reticulation posterior to the eye. Cephalic carinulae extending up to, but not beyond the medial excision (‘V’) of the posterior head margin. Promesonotal dome with a relatively low profile, mesonotal declivity short and relatively gradual. In dorsal view, promesonotum weakly punctate, anterior portion with distinct transverse carinulae. Although we tentatively treat the specimen from California (CASENT0005742) as *Pheidole navigans*, it differs morphologically from the aforementioned specimens and bears closer resemblance to Pheidole exigua var. tuberculata Mayr (currently synonymized under *Pheidole flavens*).

The similarity of these northern hemisphere specimens to the one from Paraguay raises the possibility that these putatively conspecific populations originated in South America. Indeed, the Paraguay specimen was collected in the Reserva Natural del Bosque Mbaracayú near the Río Paraná – a region infamous for serving as a cradle of ant invasion (Suarez and Tsutsui 2008).

#### Biology.

In Florida, Naves (1985) reported *Pheidole navigans* (as *Pheidole moerens*) nesting under boards, at base of oak trees and fence posts, along roots, under palm leaves, inside wall crevices, and rarely in the ground. The chambers are built with small soil or debris particles and have small openings. Most nuptial flights occur in July. The species was found to practice dependent nest founding, but became monogynous before the first brood was reared. Mature colonies can support over 100 majors and over 500 workers. They feed on seeds and scavenge and prey on small dead or live arthropods, and forage very close to the nesting sites. Deyrup (2000) also provided observations of this species (as *Pheidole moerens*) from Florida, adding that it occurs in both disturbed areas and mesic or moist woods, also nests in hollow twigs, nuts and in leaf litter, and is occasionally arboreal.

#### Distribution.

The precise native range of *Pheidole navigans* is unknown, but it is certainly of Neotropical origin. The record of the species from the Paraná region of South America suggests it could be South America. We tentatively treat both known South American records (Paraguay and Venezuela) as native, and the Mexican record as introduced, but other scenarios are equally possible. *Pheidole navigans* was first reported as introduced in the United States by M.R. Smith (1967) under the name *Pheidole moerens*. The name *Pheidole moerens* has since been applied to North American records from Alabama (Glancey et al. 1976; Smith 1967), California (Garrison 1996; Martínez 1997), Florida (Deyrup et al. 1988; Deyrup et al. 2000; Wojcik et al. 1975), Louisiana (Dash and Hooper-Bùi 2008), Mississippi (MacGown and Hill 2010), North Carolina (Guénard et al. 2012) and Texas (Wilson 2003). We tentatively treat all of these records as *Pheidole navigans*, but the California and Texas records could also belong to another species in the *flavens* complex. In the Pacific, *Pheidole navigans* is established in Hawaii (Gruner et al. 2003). We cannot confirm whether the *Pheidole moerens* records from Cocos Island (Solomon and Mikheyev 2005) or the indoor records from a butterfly house in the northwestern United States (collection code KRW26Feb99) refers to *Pheidole navigans* or another member of the *flavens* complex.

#### Risk statement.

The species most often referred to as *Pheidole moerens* in the southeastern United States, and treated here as *Pheidole navigans*, has been expanding its range since it was first reported in Alabama in 1967. However, this species is not considered a major pest and is only occasionally reported to enter houses (Deyrup et al. 2000). In Louisiana *Pheidole navigans* is considered a pest (Dash and Hooper-Bùi 2008). *Pheidole navigans* could become more regionally and possibly globally widespread in the future.

### Pheidole noda

Taxon classificationAnimaliaHymenopteraFormicidae

F. Smith

Figs 81 , 88i

Pheidole ***noda***. *Pheidole nodus* Smith, F. 1874: 407 (s.) JAPAN, Hyogo. Forel 1900: 268 (w.); Wheeler, W.M. 1906: 309 (q.); Ogata 1982: 196 (m.); Wheeler, G.C. & Wheeler, J. 1953: 75 (l.).
Pheidole *rhombinoda*. *Pheidole rhombinoda*Mayr 1879: 678 (s.) INDIA, Calcutta [NHMW]. Bingham 1903: 251 (q.). Subspecies of *noda*: Wheeler, W.M. 1929: 3; Santschi, 1937: 371. Junior synonym of *noda*: Yasumatsu 1962: 96. [Misspelled as *rhomboida* by Santschi 1925: 83.]
Pheidole *micantiventris*. Pheidole rhombinoda var. micantiventrisMayr 1897: 427 (s.) SRI LANKA. Junior synonym of *noda*: Yasumatsu 1962: 96.
Pheidole *taprobanae*. Pheidole rhombinoda var. taprobanaeForel 1902c: 178 (s.), 195 (w.) SRI LANKA (Yerbury) [MHNG]. [Unresolved junior primary homonym of *taprobanae* Smith, F. 1858: 175.] [Also described as new by Forel 1902b: 544.] Subspecies of *rhombinoda*: Forel 1913b: 662; of *noda*: Santschi 1937: 371. Junior synonym of *noda*, lectotype designated: Eguchi 2008: 59.
Pheidole *treubi*. *Pheidole treubi*Forel 1905a: 19 (s.q.) INDONESIA, Bogor [Buitenzorg], Java [MHNG]. Junior synonym of *noda*, lectotype (s.) designated: Eguchi 2001b: 18.
Pheidole *stella*. Pheidole rhombinoda subsp. stellaForel 1911c: 380 (s.) INDIA, Sikkim, Himalaya, 1200 m [MHNG]. Subspecies of *noda*: Wheeler, W.M. 1929f: 3. Junior synonym of *noda*, lectotype (s.) designated: Eguchi 2008: 59.
Pheidole *formosensis*. Pheidole rhombinoda var. formosensisForel 1913a: 193 (s.w.q.m.) TAIWAN, Kankau, [MHNG] (H. Sauter). Subspecies of *noda*: Santschi 1937: 370. Junior synonym of *noda*: Eguchi 2008: 59.
Pheidole *praevexata*. Pheidole nodus var. praevexata Wheeler W.M. 1929: 3 (s.w.q.) JAPAN, Okayama (H. Sauter). Junior synonym of *noda*: Yasumatsu 1962: 96.
Pheidole Pheidole nodus st. rhombinoda var. gratiosaSantschi 1937: 371, unavailable name. Material referable to this form: Eguchi 2008: 59.
Pheidole *flebilis*. Pheidole nodus var. flebilisSantschi 1937: 370 (s.w.) TAIWAN, Hori [NHMB]. Junior synonym of *noda*: Eguchi 2008: 59.

#### Diagnosis among introduced *Pheidole*.

Medium to dark reddish brown. **Major** HW 1.58–1.82, HL 1.69–1.91, SL 1.00–1.12, CI 93–98, SI 56–65 (n=5, Eguchi 2008). Head subquadrate (Fig. 7). Head rugoreticulate on posterolateral lobes and laterad of frontal carinae (Fig. 13a), but frons dominated by long, well-organized and parallel longitudinal rugae (Fig. 13b). Antennal scrobes indistinct to moderately impressed, but frontal carinae always forming a border capable of accepting the antennal scape (Fig. 13c). Promesonotum in profile with two convexities (Fig. 5), the large anterior dome in addition to a distinct mound or prominence on the posterior slope. Postpetiole forming a high dorsally bulging dome that is tallest at midpoint (Fig. 2a); ventral margin flat to very weakly convex (Fig. 2b). **Minor** HW 0.57–0.66, HL 0.71–0.82, SL 0.91–1.07, CI 80–82, SI 157–162 (n=5, Eguchi 2008). Head predominantly glossy (Fig. 36), lacking punctation and or rugae above eye level. Posterior head margin strongly convex (Fig. 44). Antennal scapes long (e.g. Fig. 39), but not surpassing the posterior head margin by more than 2× eye length. Promesonotum in profile with two convexities, the large anterior dome (Fig. 43a) in addition to a distinct prominence on the posterior slope (Fig. 43b). Petiole and postpetiole glossy to very weakly sculptured laterally (Fig. 48). Postpetiole forming a high dorsally bulging dome that is tallest at midpoint; ventral margin flat to very weakly convex (Fig. 2).

#### Identification, taxonomy and systematics.

*Pheidole noda* is a large, long-limbed, dark colored species most easily recognized by its distinctly enlarged dome-like postpetiole. The species belongs to a clade of large-bodied species that has diversified across Indomalaya (Economo et al. 2015). Although both *Pheidole noda* and *Pheidole megacephala* are considered to have an enlarged postpetiole, they are very different in shape. That of the former is dome-like (Fig. 2) and that of the latter has an anteroventral bulge in addition to the posterodorsal bulge (Fig. 1). The majors of *Pheidole noda* are easily separated from those of *Pheidole megacephala* by the strongly sculptured face (Fig. 8 *vs.* Fig. 9). The minors both have glossy faces, but those of *Pheidole noda* are larger with relatively longer antennal scapes (Fig. 39 *vs.* Fig. 40). *Pheidole noda* is occasionally confused with other Asian tramp *Pheidole*, including *Pheidole fervens* and *Pheidole indica*, but both major and minor workers are easily separated from these by the enlarged postpetiole. Readers are referred to Eguchi (2008) for characters used to separate *Pheidole noda* from its other Asian congeners.

#### Biology.

Despite being a relatively common species across its native range, little is known about the biology of *Pheidole noda*. The species is apparently easy to keep in laboratory settings, and Yamamoto et al. (2009) reported that they kept a colony with five dealated queens, suggesting dependent colony foundation or polygyny. The authors also noted that in Japan it nests in the ground but also forages in vegetation. *Pheidole noda* was the most frequent visitor to extrafloral nectaries of *Mallotus japonicus* in an experiment conducted in Japan (Yamawo et al. 2012). Eguchi (2008) observed that *Pheidole noda* occurs from open lands to relatively developed forests, and nests in the soil, under shelters on the ground, and in rotting logs. Eguchi (2004a) noted that the species takes seeds of sesame and amaranth put on the ground, and majors serve as repletes. During a recent survey in Yunnan, China, the species was found to occur in rubber tree plantations and rainforest between 550 and 1219 m (Liu et al. 2015).

#### Distribution.

*Pheidole noda* is considered native across mainland Asia, occurring from western India east to Japan. Forel (1903) reported the species from the Andaman Islands but it was not recovered during a more recent survey of the islands (Mohanraj et al. 2010). There is geographic disjunction between the mainland Asia population and the populations from the southern islands of Indonesia. The majors of the Indonesian taxon, originally described as *Pheidole treubi* Forel, were considered a distinct population by (Eguchi 2001b), but conspecific with *Pheidole noda*. Although not included on the map, if verified, the records from the Russian Far East (Kupianskaia 1990) would be the most northern extent of the native range. The dispersive capacity of *Pheidole noda* is demonstrated by its colonization of Volcano Island (Nishino-shima Island), which is 22 ha in size and located 1,000 km south of mainland Japan. The island erupted in 1973, virtually eradicating all life. *Pheidole noda* was the only ant species discovered during the 1983 survey, and was one of only two discovered during the 2004 survey (the other being *Tetramorium bicarinatum*).

The only confirmed record of *Pheidole noda* occurring outside of its putative native range is from a glasshouse in Italy (Limonta and Colombo 2003), where it was found together with *Pheidole megacephala* and *Tetramorium bicarinatum* on nursery plants imported from Asia. The species was also found on plant material imported from Asia and intercepted at quarantine facilities in Washington and Hawaii.

#### Risk statement.

*Pheidole noda* is not considered an agricultural, ecological or structural pest species, although it is often associated with disturbed habitats. The species is also not known to have established outdoors beyond its native range. However, perhaps because it can be easily maintained in artificial nests, colonies with laying queens listed as *Pheidole noda* and Pheidole cf. noda are available for sale from businesses advertising on the internet. The shipment of this species outside its native range to hobbyists increases its chances of accidental release into non-native habitats.

### Pheidole obscurithorax

Taxon classificationAnimaliaHymenopteraFormicidae

Naves

Figs 82 , 88j

Pheidole ***obscurithorax***. Pheidole fallax subsp. obscurithoraxNaves 1985: 61 (s.w.) ARGENTINA, Alta Gracia, Córdoba (Bruch). [First available use of Pheidole fallax st. arenicola var. obscurithoraxSantschi 1923: 58; unavailable name.] Raised to species; lectotype (s.) (CASENT0913311, NHMB) designated: Wilson 2003: 331.

#### Diagnosis among introduced *Pheidole*.

Medium reddish brown to dark brown. **Major** HW 1.47–1.70, HL 1.49–1.84, SL 0.98–103, CI 92–99, SI 58–70 (n=3). Head subquadrate (Fig. 7); almost entirely covered by a network of intersecting rugae (Fig. 12a), lacking long, well-organized and parallel longitudinal rugae on the frons (Fig. 12b). Frontal carinae indistinct, quickly becoming integrated into dense rugoreticulum that covers the entire face. Antennal scrobes entirely lacking. Antennal insertions surrounded by deeply excavated pits (Fig. 12c). Head often a lighter reddish brown than the mesosoma. Promesonotum in profile with two convexities (Fig. 5), the large anterior dome in addition to a distinct mound or prominence on the posterior slope. Postpetiole not swollen relative to petiole (Fig. 3). **Minor** HW 0.60–0.67, HL 0.78–0.85, SL 0.94–1.08, CI 76-82, SI 152–173 (n=5). Head predominantly glossy (Fig. 36), lacking punctation and or rugae above eye level. Posterior margin strongly convex in full-face view such that the head outline forms a single unbroken curve from eye to eye (Fig. 44). Antennal scapes extremely long, surpassing posterior head margin by more than 2× eye length (Fig. 39). Promesonotum in profile with two convexities, the large anterior dome (Fig. 43a) in addition to a distinct prominence on the posterior slope (Fig. 43b). Mesopleuron mostly sculptured. Postpetiole not swollen relative to petiole (Fig. 3). Petiole and postpetiole strongly sculptured laterally (Fig. 47).

#### Identification, taxonomy and systematics.

*Pheidole obscurithorax* is a member of the New World (and polyphyletic, see Moreau 2008) *Pheidole fallax* species group defined by Wilson (2003). It is a large dark species over 6 mm in body length. The species is easily distinguished from *Pheidole megacephala* by the much larger body size and relatively reduced postpetiole, in addition to the strongly sculptured head of the major worker (Fig. 12), and the much longer antennal scapes of the minor. It is separated from other New World species treated here, including those of the *Pheidole punctatissima* clade and *Pheidole flavens* complex, by the much larger size, prominence on the posterior slope of the promesonotum (Fig. 5, major; Fig. 50, minor), densely rugoreticulate face of the major (Fig. 12), and smooth head and long antennal scapes of the minor. The Old World species *Pheidole fervens*, *Pheidole indica*, and *Pheidole noda* all have majors with strongly sculptured head and minors with smooth heads, and the reader is referred to the key for characters used to separate these from *Pheidole obscurithorax*.

#### Biology.

In its introduced range of the southeastern United States, *Pheidole obscurithorax* is characterized by its large size, large nest mounds, very active foraging and fast recruitment to bait such as cookie crumbs (King and Tschinkel 2007). It nests in soil in open areas, where it produces conspicuous nests, each generally with a single large opening often covered by a leaf or other collected material (Storz and Tschinkel 2004). The species is an omnivorous scavenger of dead arthropods (possibly including dead fire ants), and less frequently of plant material such as flower petals (Storz and Tschinkel 2004). Studies in its introduced range found evidence that *Pheidole obscurithorax* is monogynous and is spreading by natural dispersal of winged females in addition to human-mediated long-distance dispersal (King and Tschinkel 2007). The species was most often found associated with disturbed habitats such as lawns and roadsides, but there are also records of it occurring in natural areas such as hardwood forests (Wilson 2003). However, its steady expansion across the southeastern United States and co-occurrence with *Solenopsis invicta* suggest it is an important species to monitor.

#### Distribution.

*Pheidole obscurithorax* is presumed native to the South American region of Argentina, Paraguay and southern Brazil that includes the Paraguay, La Plata and Parana Rivers. This flood-prone area is the cradle of many other well-known invasive ants including fire ants (*Solenopsis invicta* Buren and *Solenopsis richteri* Forel), the Argentine ant (*Linepithema humile*), and many lesser-known species that were anthropogenically introduced (King and Tschinkel 2007; Storz and Tschinkel 2004; Suarez and Tsutsui 2008; Wilson 2003). Most of these species, including *Pheidole obscurithorax*, were first introduced to North America via the Mobile, Alabama shipping port pathway. *Pheidole obscurithorax* was introduced to Mobile, Alabama around 1950 (Naves 1985) and subsequently expanded its range to include Florida, Georgia, Mississippi and Texas (Storz and Tschinkel 2004; Wilson 2003). Additional occurence records, including the first record for Bolivia, were published (Wetterer et al. 2015) just as this manuscript was going to press, and were not included in the present study.

#### Risk statement.

*Pheidole obscurithorax* is not currently considered a pest in its introduced range, as it does not sting and is not known to infest dwellings or structures (King and Tschinkel 2007). However, the species is an aggressive predator (Deyrup et al. 2000) and may have the potential to become a pest or to negatively impact native species if its populations continue to grow and spread. *Pheidole obscurithorax* is thought to spread across the southeastern United States by mated queens (not colony fragments) that are being transported in substrates such as potted plants. It is possible that *Pheidole obscurithorax* could become more widespread regionally and globally in the future.

### Pheidole parva

Taxon classificationAnimaliaHymenopteraFormicidae

Mayr

Fig. 83 , Fig. 88K

Pheidole ***parva***. *Pheidole parva*Mayr 1865: 98, pl. 4, fig. 28 (s.w.) SRI LANKA [NHMW]. Bingham 1903: 245 (q.).
Pheidole *decanica*. Pheidole parva var. decanicaForel 1902c: 175 (s.), 192 (w.q.m.) INDIA, Cochin (Rothney) [MHNG]. [Also described as new by Forel 1902b: 542.] Junior synonym of *parva*; lectotype designated: Eguchi, Yamane & Zhou 2007: 261.
Pheidole *sauteri*. *Pheidole sauteri* Wheeler, W.M. 1909: 334 (s.w.) TAIWAN, Kaoshung (H. Sauter) [MCZC cotype 20671] Junior synonym of *parva*: Eguchi, Yamane & Zhou 2007: 262.
Pheidole *mala*. Pheidole rinae var. malaForel 1911b: 205 (s.w.) INDONESIA, Semarang, Java (Jacobson) [MHNG]. Lectotype (s.) designated: Eguchi 2001a: 39. Junior synonym of *parva*: Eguchi, Yamane & Zhou 2007: 262.
Pheidole *tipuna*. Pheidole rinae r. tipunaForel 1912: 68 (s.w.) TAIWAN, Takao (H. Sauter) [MHNG]. Junior synonym of *parva*; lectotype (s.) designated: Eguchi, Yamane & Zhou 2007: 262.
Pheidole *bugi*. *Pheidole bugi* Wheeler, W.M. 1919: 66 (s.w.) MALAYSIA, Sarawak, Borneo (R. Thaxter) [MCZC cotype-8947]. Lectotype (s.) designated: Eguchi 2001a: 37. Junior synonym of *parva*: Eguchi, Yamane & Zhou 2007: 262.
Pheidole *farquharensis*. Pheidole flavens var. farquharensis Forel 1907: 91 (w.) SEYCHELLES, Farquhar Atoll, v–xii.1905 (J.S. Gardiner) [BMNH]. Junior synonym of *parva*: Fischer and Fisher 2013: 340.
Pheidole *tarda*. Pheidole (Pheidole) tardusDonisthorpe 1947: 285 (q.) MAURITIUS, Rose Hill, 07.v.1946 (R. Mamet) [BMNH]. Junior synonym of *parva*: Fischer and Fisher 2013: 341.

#### Diagnosis among introduced *Pheidole*.

Yellowish brown to dark brown. **Major** HW 0.85–0.92, HL 0.96–1.07, SL 0.41–0.45, CI 85–92, SI 45–51 (n=11, Eguchi et al. 2007). Head subquadrate (Fig. 7). Posterolateral lobes, including posterior head margin, covered in rugoreticulum (Fig. 26). Antennal scrobes indistinct to moderately impressed, but frontal carinae always forming a border capable of accepting the antennal scape (Fig. 13c). Promesonotum in profile forming a single dome (Fig. 4), lacking a distinct mound or prominence on the posterior slope. Promesonotum in dorsal view transverse with strongly projecting shoulders (Fig. 28). Promesonotal dorsum rugoreticulate with distinct long longitudinal striae in addition to shorter sections of transverse and intersecting striae (Fig. 22). Postpetiole not swollen relative to petiole (Fig. 3). **Minor** HW 0.39–0.50, HL 0.43–0.54, SL 0.38–0.46, CI 88–94, SI 84–102 (n=17, Eguchi et al. 2007). Posterior portion of head with many short to medium length segments of striae distinctly interlaced among punctate ground sculpture (Fig. 59). Antennal scapes with erect to suberect hairs (Fig. 56); scapes do not surpass posterior head margin (Fig. 41). Promesonotum in profile forming a single dome (Fig. 42), lacking a distinct mound or prominence on the posterior slope. Pronotal humeri angular (Fig. 28). Hairs on mesosoma fine, flexuous, of unequal length and not arranged in pairs (Fig. 54). Postpetiole not swollen relative to petiole (Fig. 3); postpetiole narrow in dorsal view, only slightly broader than petiole (Fig. 61).

#### Identification, taxonomy and systematics.

*Pheidole parva* is a very small and inconspicuous species that is thus far reported only from Asia, a few localities in Arabia, and the islands of the Indian Ocean and the Pacific Ocean. It belongs to an Old World clade scattered across Indomalaya and into Oceania, and was treated as part of the *Pheidole rinae* complex by Eguchi et al. (2007). The minor workers are completely covered in punctate sculpture and are difficult to differentiate from those of the Neotropical *Pheidole flavens* complex. The similarity is so close that an introduced population of *Pheidole parva* from the Seychelles was described by Forel, on the basis of the minor worker, as Pheidole flavens var. farquharensis. The similarity is entirely convergent, as these lineages are distantly related. *Pheidole parva* minors can be separated from those of the *Pheidole flavens* complex most reliably by the interrupted striae that are interlaced among the punctate ground sculpture of the posterior head (Fig. 59 *vs.* Fig. 60). This character can also be viewed in the dorsal view. *Pheidole parva* minors can be separated from those of the *Pheidole punctatissima* clade treated here by the glossy gaster (Fig. 32 *vs.* Fig. 33) and finer mesosomal hairs of unequal length (Fig. 54 *vs.* Fig. 53). The major workers are characterized by a defined and moderately depressed antennal scrobe and a thick network of reticulated rugulae on the posterior lobes. This pattern is most similar to that of the broadly sympatric *Pheidole fervens* and *Pheidole indica*, but *Pheidole parva* is much smaller than those species (HW < 0.95 mm *vs* HW > 1.10 mm) and lacks the distinct prominence on the posterior slope of the promesonotal dorsum (Fig. 4 *vs.* Fig. 5). The majors of *Pheidole parva* can be separated from those of the *Pheidole flavens* and *Pheidole punctatissima* group species treated here by the much stronger and more reticulated carinae which reach the posterior margin (Fig. 26 *vs.* Fig. 25 and Fig. 27) in addition to other characters given in the key. Readers are referred to Eguchi (2008; 2007) for characters separating *Pheidole parva* from its Asian congeners.

#### Biology.

Little is known about the biology of *Pheidole parva*, but it does appear to be expanding its range and is worth monitoring in the future as it exhibits a high tolerance for disturbance. Eguchi (2008) observed that the species seems to inhabit open lands and forest edges, and has probably expanded its range in some part as the result of human commerce. *Pheidole parva* was one of the most commonly collected ants in a myrmecological study of agricultural fields in Vietnam and Okinawa (Anh et al. 2010; Suwabe et al. 2009). A recent study of 18 structure invading pest ants of healthcare facilities in Singapore found *Pheidole parva* the most frequently encountered species (Man and Lee 2012). *Pheidole parva* and *Pheidole megacephala* were the two most common ant species encountered and together accounted for over 50% of the total collection (25.9% and 25.2%, respectively). In Mauritius and the Seychelles *Pheidole parva* can be locally abundant and can be found in soil and leaf litter, under stones or root mats, in rotten logs, foraging on or nesting in the ground, as well as in lower vegetation and even under the bark of live trees (Fischer and Fisher 2013). It was collected there in parks, gardens, mangrove, coastal scrub, degraded dry forest, littoral and mixed forest, and rainforest, in elevations between 1–445 m. It was collected inland on the Arabian Peninsula from date tree orchards, banana plantations and under potted plants between 675–735 m elevation (Fischer and Fisher 2013).

#### Distribution.

*Pheidole parva* is considered here as native to the Indo-Malay region. The species is recorded from the Asian mainland from India east to China. We consider the records from Indonesia, Borneo, the Philippines and Taiwan to be native, but much of this distribution could represent a more recent anthropogenic expansion. We consider the records from the Okinawa and Kagoshima prefectures of Japan to be introduced along with the records from Palau to represent introduced populations, but it is difficult to know whether the species arrived in these islands before, with or after the arrival of humans. The species is introduced in the Seychelles, Mauritius, Saudi Arabia and the United Arab Emirates (Fischer and Fisher 2013). *Pheidole parva* was also collected from hothouses in Austria and Germany.

#### Risk statement.

*Pheidole parva* is not currently considered to be a significant pest species, and no impacts on agricultural systems or native ecosystems have been documented as of yet. The species is known to invade structures, however, and its prevalence in Singapore health care facilities (Man and Lee 2012) suggests it could become a more widespread nuisance pest in the future. Live colonies have been reported from various ships (Fischer and Fisher 2013) and should be screened for during quarantine inspections.

### Pheidole proxima

Taxon classificationAnimaliaHymenopteraFormicidae

Mayr

Fig. 84 , 88L

Pheidole ***proxima***. *Pheidole proxima*Mayr 1876: 104 (s.w.) AUSTRALIA, Peak Downs, Queensland [NHMW, examined]. Current subspecies: nominal plus *bombalensis*, *transversa*.

#### Diagnosis among introduced *Pheidole*.

Reddish brown. **Major** HW 0.95–1.05, HL 1.04–1.21, SL 0.44–0.50, CI 87–92, SI 42–52 (n=4). Head subquadrate (Fig. 7). Posterolateral lobes lacking sculpture (including foveolate ground sculpture, carinae and rugae) posterior to maximum extent of antennal scapes in repose (Fig. 9). Head glossy, lacking foveolate ground sculpture. Hypostomal bridge with a small median tooth in addition to a pair of larger inner teeth (Fig. 18). Promesonotum in profile forming a single dome (Fig. 4), lacking a distinct mound or prominence on the posterior slope. Promesonotal dorsum glossy, lacking foveolate ground sculpture or striae. Pronotal striae in dorsal view mostly absent (Fig. 23). Metapleuron with moderate rugulae and some weak punctation (Fig. 16). Petiolar node strongly punctate (Fig. 16). Postpetiole not swollen relative to petiole (Fig. 3). **Minor** HW 0.46, HL 0.52, SL 0.40, CI 90, SI 86 (n=1). Head predominantly glossy (Fig. 36), lacking punctation and or rugae above eye level. Posterior head margin weakly convex (Fig. 45) to weakly concave (Fig. 46) in full-face view. Antennal scapes reach but do not surpass posterior head margin (Fig. 41). Mesopleuron entirely punctate (Fig. 52a). Promesonotum in profile forming a single dome (Fig. 42), lacking a distinct mound or prominence on the posterior slope. Propodeal spines moderately produced and spiniform (Fig. 52b). Petiole distinctly sculptured except for apical portion of node. Postpetiole not swollen relative to petiole (Fig. 3).

#### Identification, taxonomy and systematics.

*Pheidole proxima* is a relatively small, brownish yellow, short-limbed species with a strongly shining integument. The phylogenetic placement of *Pheidole proxima* is unknown, but it almost certainly clusters within an Old World clade that has radiated across Australia and New Guinea. The species is slightly smaller than *Pheidole megacephala*, but both have workers with almost entirely glossy faces. The postpetiole of *Pheidole proxima* is not swollen relative to the petiole (Fig. 3), as it is in *Pheidole megacephala* (Fig. 1). The head of the major is subquadrate (Fig. 7), while that of *Pheidole megacephala* is more heart-shaped (Fig. 6). The antennal scapes of the minor do not surpass the posterior head margin (Fig. 41), as they do in *Pheidole megacephala* (Fig. 40). The other two *Pheidole* species established in New Zealand are *Pheidole rugosula* and *Pheidole vigilans*. The glossy face of *Pheidole proxima* easily separates both worker castes of from those of *Pheidole rugosula*. In addition to being significantly smaller (major HW < 1.0 mm, minor HW < 0.48 mm) than *Pheidole vigilans* (major HW > 1.2 mm, minor HW > 0.52 mm), the major of *Pheidole proxima* is more sculptured (Fig. 16 *vs.* Fig. 17), and the hypostomal bridge has a distinct median tooth (Fig. 18 *vs.* Fig. 19). The minors of *Pheidole proxima* are separated from those of *Pheidole vigilans* by the shorter scapes (Fig. 41 *vs.* Fig. 40), more sculptured mesopleuron (Fig. 52a *vs.* Fig. 51a), and more robust propodeal spines (Fig. 52b *vs.* Fig. 51b). Additional taxonomy of these species is discussed in (Berry et al. 1997).

Comparison of the *Pheidole proxima* Mayr type series and images of the two subspecies suggests that all three taxa are heterospecific. There is some reason to believe, however, that the name *Pheidole proxima* Mayr does not apply perfectly to the species recently introduced to New Zealand. The specimens examined from New Zealand conflict with Mayr’s original description and type specimens on several points. The pronotal dorsum of the type major worker is transversely rugose whereas that of the New Zealand specimens are completely glossy. Although we were unable to examine minors from the type series, Mayr described the head of the minor worker as coriaceous and striate-rugose with scapes that barely exceed the posterior margin. In contrast the minor workers from New Zealand have heads that are completely glossy and scapes that do not exceed the posterior head margin. Forel, in his description of Pheidole proxima subsp. bombalenis, describes the minor worker as identical to *Pheidole proxima* Mayr with the exception of having longer propodeal spines. The specimen images of the *Pheidole bombalensis* syntype minor show a strongly sculptured face, similar to the pattern described by Mayr. The major workers from the type series are larger than the New Zealand specimen we measured (HW 1.03–1.05 mm *vs.* HW 0.95 mm), have relatively narrower heads (CI 87–89 *vs.* CI 92), and relatively shorter antennal scapes (SI 42–46 *vs.* SI 52). While a more exhaustive survey of Australia’s *Pheidole* may reveal the New Zealand population to be more closely related to another species from that fauna, we follow Berry et al. (1997) in using *Pheidole proxima* Mayr.

#### Biology.

The only natural history published for *Pheidole proxima* was recorded by Green and Gunawardana (2006) from their work with the New Zealand incursion. They reported that *Pheidole proxima* produced large nests recognizable by tiny conical mounds of sandy or grainy material above the ground near the entrance. The size of the mounds varies with soil type, with mounds as small as 5 mm high by 200–300 mm in diameter. They are tolerant to disturbance and capable of invading structures. The minor and major workers are both active foragers and were observed recruiting to both sweet and savory baits in high numbers.

#### Distribution.

*Pheidole proxima* Mayr is native to Queensland, Australia. The sparse records of the species are scattered from Cape York at the northernmost tip of the continent down to the Gold Coast. The species is introduced to New Zealand and was first detected during a 2004 survey of the Port of Napier following an incursion of *Solenopsis invicta* (Green and Gunawardana 2006). The species is now widespread across the North Island from the Napier-Hastings area to Auckland.

#### Risk statement.

*Pheidole proxima* is at most considered a nuisance species in New Zealand on account of its ability to infest structures. However, very little is known about the species, including its impact on agricultural systems and native ecosystems. There is little reason to believe that it will become globally or regionally widespread.

### Pheidole punctatissima

Taxon classificationAnimaliaHymenopteraFormicidae

Mayr

Figs 85 , 88M

Pheidole ***punctatissima***. *Pheidole punctatissima*Mayr 1870a: 400 (s.w.) MEXICO (E. Norton) [NHMW]. Description of queen: Forel 1908: 52. Lectotype (major worker, CASENT0601256) designated: Longino and Cox 2009: 41. See also: Wilson 2003: 618.
Pheidole *napaea*. Pheidole punctatissima subsp. napaea Wheeler, W.M. 1934: 165 (s.w.) MEXICO, Mirador, Veracruz (E. Skwarra). Junior synonym of *punctatissima*: Brown 1981: 525.

#### Diagnosis among introduced *Pheidole*.

Body reddish brown to nearly black. **Major** HW 0.86–1.06, HL 0.94–1.13, SL 0.56–0.63, CI 92–97, SI 57–68 (n=9, Longino pers. comm.). Head bicolored with the yellowish posterior two-thirds contrasting with the darker brown anterior third and rest of body (Fig. 34). Head subquadrate (Fig. 7); often entirely foveolate (Fig. 11), but portions of posterolateral lobes can be glossy. Posterolateral lobes never with distinct rugae. Promesonotum in profile forming a single dome (Fig. 4), lacking a distinct mound or prominence on the posterior slope. Promesonotal dorsum usually foveolate and never with distinct transverse striae. Postpetiole not swollen relative to petiole (Fig. 3). Postpetiole relatively broad; distinctly more than 2× petiolar width in dorsal view (Fig. 31). Gaster with at least anterior 1/3 of first tergite matte (Fig. 33). **Minor** HW 0.44–0.50, HL 0.54–0.59. SL 0.55–0.58, CI 79–85, SI 114–125 (n=14, Longino pers. comm.). Head, including the area mesad of the frontal carinae, entirely covered by reticulated network of punctures (Fig. 37). Posterior head margin relatively narrow (Fig. 58). Antennal scapes lack standing hairs (Fig. 55); scapes surpass posterior head margin by a distance equal to or greater than eye (Fig. 40); scapes relatively long (SI 103–125). Promesonotum in profile forming a single dome (Fig. 42), lacking a distinct mound or prominence on the posterior slope. Hairs on mesosoma stout, stiff, of equal length and arranged in pairs (Fig. 53). Postpetiole not swollen relative to petiole (Fig. 3). Postpetiole broad in dorsal view, distinctly broader than petiole (Fig. 62). Gaster with at least anterior 1/3 of first tergite matte (Fig. 33).

#### Identification, taxonomy and systematics.

*Pheidole punctatissima* is a small species with entirely punctate minor workers that are usually dark red brown to nearly black. The major workers are easily recognizable by the distinct bicolored head which is dark anteriorly and yellowish white posteriorly. *Pheidole punctatissima* is a member of the Neotropical *Pheidole punctatissima* clade, together with *Pheidole anastasii* and *Pheidole bilimeki* (Economo et al. 2015). Among species treated here, it is easily confused with the aforementioned and with members of the *Pheidole flavens* complex. Minor workers can also be confused with those of *Pheidole parva*. Within the *Pheidole punctatissima* clade, the major workers of *Pheidole punctatissima* are immediately distinguished from those of both *Pheidole anastasii* and *Pheidole bilimeki* by their bicolored heads (Fig. 33). The minor workers of *Pheidole punctatissima* tend to have relatively narrower posterior head margins and longer antennal scapes than those of *Pheidole anastasii* and *Pheidole bilimeki*, but separation can be difficult. See section under *Pheidole anastasii* for identification notes.

#### Biology.

*Pheidole punctatissima* is a weedy species that tends to be arboreal and prefers open, disturbed habitat (Longino and Cox 2009). It is most commonly found nesting in dead wood on the ground or in dead tree branches. Wilson (2003) reported winged reproductives were found in nests during April and July. Specimen records retrieved from Antweb.org indicate the species was collected from 10–2500 m elevation (570 m average). *Pheidole punctatissima* has also managed to establish indoors in several European countries. Colonies were found in Denmark infesting a hospital and in Norway inhabiting private homes and a nursing home (Birkemoe and Aak 2008). Birkemoe and Aak (2008) speculated that the species was inadvertently imported along with nursery plants.

#### Distribution.

*Pheidole punctatissima* is considered here as broadly native to the Neotropics from southern Mexico to northern South America. We tentatively treat the Caribbean records as native but these might represent more recent human-mediated dispersal events. The records from southern Brazil, reported at least in part from 10 different urban centers (Lutinski et al. 2013), have not been verified with specimen examination. Should the records refer to *Pheidole punctatissima* Mayr and not one of its many morphologically similar congeners we would consider this to be an introduced population. Indoor colonies were found in Denmark and Norway (Birkemoe and Aak 2008).

#### Risk statement.

*Pheidole punctatissima* is considered a nuisance pest that can infest structures both in its native and introduced ranges (Longino and Cox 2009). The presence of this species in hospitals and nursing homes suggest it could be a potential nuisance.

### Pheidole rugosula

Taxon classificationAnimaliaHymenopteraFormicidae

Forel

Figs 86 , 88N

Pheidole ***rugosula***. Pheidole variabilis var. rugosulaForel 1902a: 423 (s.w.) AUSTRALIA, Bong-Bong, N.S.W. (Froggatt). Raised to species Berry et al. 1997: 29.
Pheidole Note: The elevation to species rank proposed by Berry et al. (1997) had been heretofore overlooked by Bolton (2014).

#### Diagnosis among introduced *Pheidole*.

Yellowish brown. **Major** HW 0.88, HL 0.94, SL 0.45, CI 94, SI 51 (n=1). Head subquadrate (Fig. 7); with distinct parallel rugae extending from frontal lobes posterior to apices of frontal carinae. Shorter lengths of rugae present across entire posterior region of head and extending to posterior margin in full-face view (Fig. 24). Promesonotum in profile forming a single dome (Fig. 4), lacking a distinct mound or prominence on the posterior slope. Promesonotal dorsum glossy with thin but distinct subparallel striae running oblique to the longitudinal midline (Fig. 20). Pronotal striae in dorsal view mostly oblique (Fig. 20). Postpetiole not swollen relative to petiole (Fig. 3). **Minor** HW 0.45, HL 0.48, SL 0.41, CI 95, SI 91 (n=1). Head with well-defined, long segments of rugae running longitudinally from below the eyes to the posterior head margin (Fig. 38). Frontal carinae distinct and reaching towards the posterior head margin, although they may occasionally be interrupted (Fig. 38). Punctate ground sculpture present on lateral surfaces of head and just mesad of the frontal carinae, but median portion of head with a large glossy section (Fig. 38). Antennal scapes reach but do not surpass posterior head margin (Fig. 41). Promesonotum in profile forming a single dome (Fig. 42), lacking a distinct mound or prominence on the posterior slope. Postpetiole not swollen relative to petiole (Fig. 3).

#### Identification, taxonomy and systematics.

*Pheidole rugosula* is a small, brownish yellow, short-limbed species with moderate head sculpturing that most likely belongs to the Australian-New Guinea clade that includes close relatives of *Pheidole variabilis* Mayr. The head sculpturing of both the major (Fig. 10) and the minor (Fig. 38) is distinct among all other *Pheidole* species treated here. These characters easily separate *Pheidole rugosula* from *Pheidole megacephala* (Fig. 24, Fig. 36). These same characters, together with a more sculptured promesonotal dorsum (Fig. 20, major) and stout propodeal spines, can be used to separate *Pheidole rugosula* from its two other congeners that are established in New Zealand, *Pheidole proxima* and *Pheidole vigilans* (which is also much larger, major HW > 1.20 mm). There is a bewildering diversity of native Australian (and to a lesser extent New Guinea) *Pheidole* that approach the morphology of *Pheidole rugosula*. Additional characters for identifying New Zealand *Pheidole* species are provided in Berry et al. (1997). A significant revision of the *Pheidole variabilis* group is required before *Pheidole rugosula* can be reliably separated from these species.

#### Biology.

In New Zealand, *Pheidole rugosula* is strongly associated with human disturbance and is the most commonly encountered of the four *Pheidole* species established in New Zealand (Berry et al. 1997). It has been recorded frequently from gardens, orchards, structures and urban areas. The species was reported nesting in compost, in the soil of vegetable gardens, in the soil of lawns, and near rubbish baskets (Berry et al. 1997; Harris et al. 2005b). It has also been recorded as scavenging dead arthropods, human food waste, nuts and seeds, and is often found associated with rotting fruit (Berry et al. 1997; Harris et al. 2005b). Other collection records suggest *Pheidole rugosula* will forage arboreally. Berry et al. (1997) also mention that label data suggests the species was collected several times attacking ootheca of mantids, including those of the native mantid *Orthodera novaezealandiae* (Colenso).

#### Distribution.

*Pheidole rugosula* is believed to be native to the New South Wales region of Australia. The only country where the species has established is New Zealand (Berry et al. 1997). Berry et al. (1997) published museum records of *Pheidole rugosula* from New Zealand. The first known occurrence of *Pheidole rugosula* in New Zealand is from Takapuna, where it was collected in 1958 and it had reached Auckland by 1963. Since then it has been found across the Auckland and Waikato regions.

#### Risk statement.

*Pheidole rugosula* is considered to be a nuisance pest around urban areas in New Zealand (Harris et al. 2005b), where foragers are attracted to pet food left out and to windfall fruit. Although it occurs in native habitats in New Zealand, its impacts are unknown. Collection data indicating a *Pheidole rugosula* attack of native mantids suggest it could have some negative impact on native biodiversity, however.

### Pheidole vigilans

Taxon classificationAnimaliaHymenopteraFormicidae

(F. Smith)

Figs 87 , 88O

Pheidole ***vigilans***. *Atta vigilans* Smith, F. 1858: 166 (w.) AUSTRALIA, Melbourne [BMNH, MCZC]. Combination in *Aphaenogaster*: Dalla Torre 1893:108; in *Pheidole*: Emery 1915a: 69.
Pheidole *dolichocephala*. *Pheidole dolichocephala*André 1896: 262 (s.) AUSTRALIA, Western Australia [MNHN]. Junior synonym of *vigilans*: Brown 1971: 13.
Pheidole *parallela*. Pheidole ampla var. parallelaForel 1902a: 435 (s.w.m.) AUSTRALIA, N.S.W. (Froggatt) [ANIC]. Junior synonym of *vigilans*: Brown 1971: 13.
Pheidole *yarrensis*. Pheidole ampla var. yarrensisForel 1902a: 434 (s.w.q.) AUSTRALIA, Yarra districts, Victoria (Froggatt) [MHNG]. Junior synonym of *vigilans*: Brown 1971: 13.
Pheidole *norfolkensis*. Pheidole ampla subsp. norfolkensis Wheeler, W.M. 1927: 134, fig. 3 (s.w.) AUSTRALIA, Norfolk Island (A.M. Lea) [MCZC]. Donisthorpe 1941: 91 (q.m.). Junior synonym of *vigilans*: Brown 1971: 13.

#### Diagnosis among introduced *Pheidole*.

Smooth yellowish to reddish brown. **Major** HW 1.30, HL 1.43, SL 0.68, CI 91, SI 52. Head subquadrate (Fig. 7); glossy, lacking foveolate ground sculpture. Posterolateral lobes lacking sculpture (including foveolate ground sculpture, carinae and rugae) posterior to maximum extent of antennal scapes in repose (Fig. 9). Hypostomal bridge with two well-developed inner teeth but lacking a median tooth (Fig. 19). Promesonotal dorsum glossy, lacking foveolate ground sculpture or striae. Promesonotum in profile forming a single dome (Fig. 4), lacking a distinct mound or prominence on the posterior slope. Metapleuron almost completely glossy with strongly reduced carinulae and lacking punctation (Fig. 17). Petiolar node mostly glossy (Fig. 17), not covered by punctate sculpture. Postpetiole not swollen relative to petiole (Fig. 3). **Minor** HW 0.55, HL 0.58, SI 0.55, CI 95, SI 101. Head predominantly glossy (Fig. 36), lacking punctation and or rugae above eye level. Antennal scapes surpass posterior head margin by approximate distance of eye length (Fig. 40). Mesopleuron entirely glossy (Fig. 51a). Promesonotum in profile forming a single dome (Fig. 42), lacking a distinct mound or prominence on the posterior slope. Propodeal spines weakly produced and dentiform (Fig. 51b). Petiole almost entirely glossy. Postpetiole not swollen relative to petiole (Fig. 3).

#### Identification, taxonomy and systematics.

*Pheidole vigilans* is a large, light colored, glossy species native to Australia and introduced in New Zealand. The species belongs to an Old World clade centered in Australia. The glossy head of the majors and minors give it a superficial appearance to *Pheidole megacephala*, but it is substantially larger than that species. Additionally, the postpetiole of *Pheidole proxima* is not swollen relative to the petiole (Fig. 3) as in *Pheidole megacephala* (Fig. 1), and the head of the major is subquadrate (Fig. 7), while that of *Pheidole megacephala* is more heart-shaped (Fig. 6). Readers are referred to the section under *Pheidole proxima* and *Pheidole rugosula* for a discussion of how to differentiate it from the other *Pheidole* species established in New Zealand. Additional taxonomy of these species is discussed in (Berry et al. 1997). Within Australia, there are many taxa similar to *Pheidole vigilans* and its close relative *Pheidole ampla* Forel. However, a revision of that fauna is required before it can be reliably diagnosed there.

#### Biology.

Records show it has established in urban areas and been found with fruit, in gardens, indoors and nesting in failing pasture (Berry et al. 1997).

#### Distribution.

*Pheidole vigilans* is considered endemic to the south eastern corner of Australia (Brown 1971). Heterick et al. (2013) reported *Pheidole vigilans* as introduced to Perth in Western Australia. The species was first collected outside of Australia in Kerikeri, New Zealand in 1956, and remains the least frequently collected *Pheidole* species in New Zealand (Berry et al. 1997; Cumber 1959). Although Pheidole ampla subsp. norfolkensis Wheeler was originally described as endemic to the Norfolk Islands, Brown (1971) later proposed that the species was introduced to those islands.

#### Risk statement.

*Pheidole vigilans* is not considered a pest in New Zealand, but it has been collected from urban areas and may be a minor garden nuisance (Harris et al. 2005c).

### Dubious and erroneous records of introduced *Pheidole* species

The following species were reported by McGlynn (1999) as introduced in Hawaii based on Nishida (1996a): *Pheidole barbata* W.M. Wheeler, *Pheidole fervida* F. Smith, *Pheidole hyatti* Emery, *Pheidole punctatissima*, and *Pheidole noda*. These records refer to quarantine interceptions (Wheeler 1934b), and it is doubtful that any of the aforementioned species ever established in Hawaii. We propose that *Pheidole barbata*, and *Pheidole hyatti* be removed from future lists of tramp ants. The URL referred to in Nishida (1996a) is obsolete, but the data is available at Nishida (1996b).

*Pheidole fervida* is also reported by McGlynn (1999) as introduced in Tahiti based on putative specimens at the LACM. These records are unverified, however, and it is possible that the Tahiti specimens actually refer to *Pheidole fervens*. While *Pheidole fervida*, as it is currently recognized (e.g. Eguchi 2008) is reported as occurring widely across Asia (Guenard and Dunn 2012), we do not consider any of these records to represent recent introductions outside of its native range. However, the species does exhibit synanthropic habits and may yet prove itself as an important tramp ant.

*Pheidole guineensis* Fabricius is a West African species that has likely never established outside of its native range. The species most often misidentified in museum collections and in the literature as *Pheidole guineensis* is *Tetramorium bicarinatum* (Nylander). Between 1862 (Mayr 1862; Roger 1862) and 1977 (Bolton 1977) The latter species was universally and mistakenly referred to as *Tetramorium guineensis* Fabricius. Readers are referred to Bolton (1977) for a thorough explanation of the taxonomic history.

*Pheidole micula* Wheeler is a species native to the southwestern United States (Moody and Francke 1982; Wheeler and Wheeler 1973; Wilson 2003). Ward (2005) unintentionally reported the species as introduced in California (pers. comm. December 17, 2013).

*Pheidole umbonata* Mayr is reported by McGlynn (1999) as introduced to Polynesia and possibly New Caledonia. Although this synanthropic species is widely distributed across Oceania, and is very tolerant of disturbed habitats (Sarnat and Economo 2012), we consider *Pheidole umbonata* to be native throughout the Pacific. Morphological (Wilson and Taylor 1967) and molecular (unpublished) variation throughout the Pacific populations suggest that the species is not a recent introduction to any of the Polynesian, Micronesia or Melanesian countries where it is found. The New Caledonia record reported by Emery (1914) was determined by Wilson and Taylor (1967) as referring to a different species. Although we are unaware of any confirmed records from New Caledonia, it is quite possible the species does occur there.

## Plates

**Figure 74.**
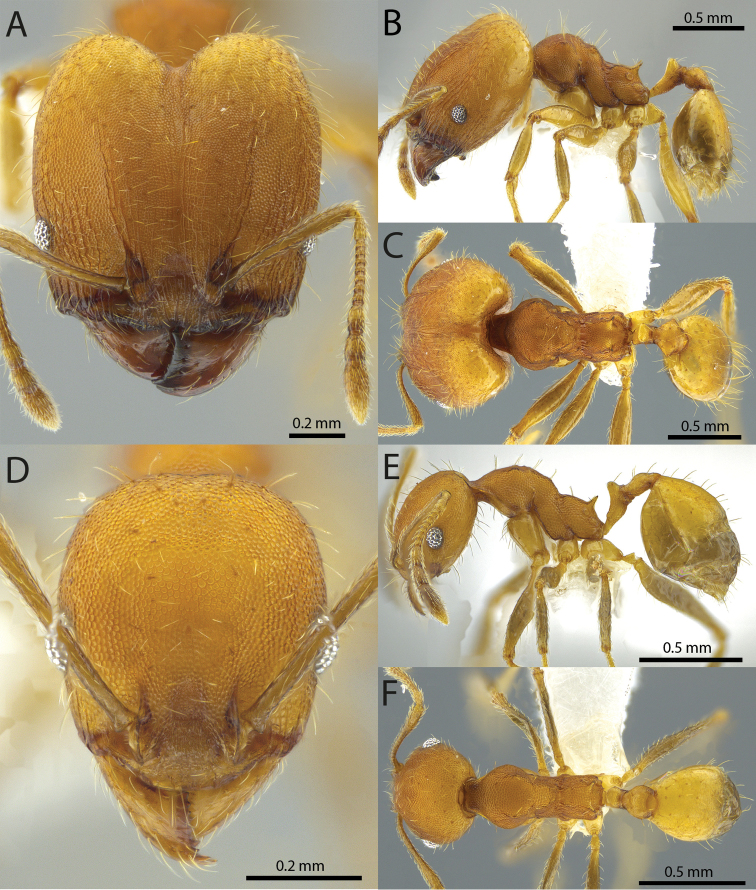
*Pheidole anastasii* Mayr. Major worker, CASENT0613680: **A** full-face view **B** lateral view **C** dorsal view. Minor worker, CASENT0619900: **D** full-face view **E** profile view **F** dorsal view. From Antweb.org, photograph by Jeremy Pillow.

**Figure 75.**
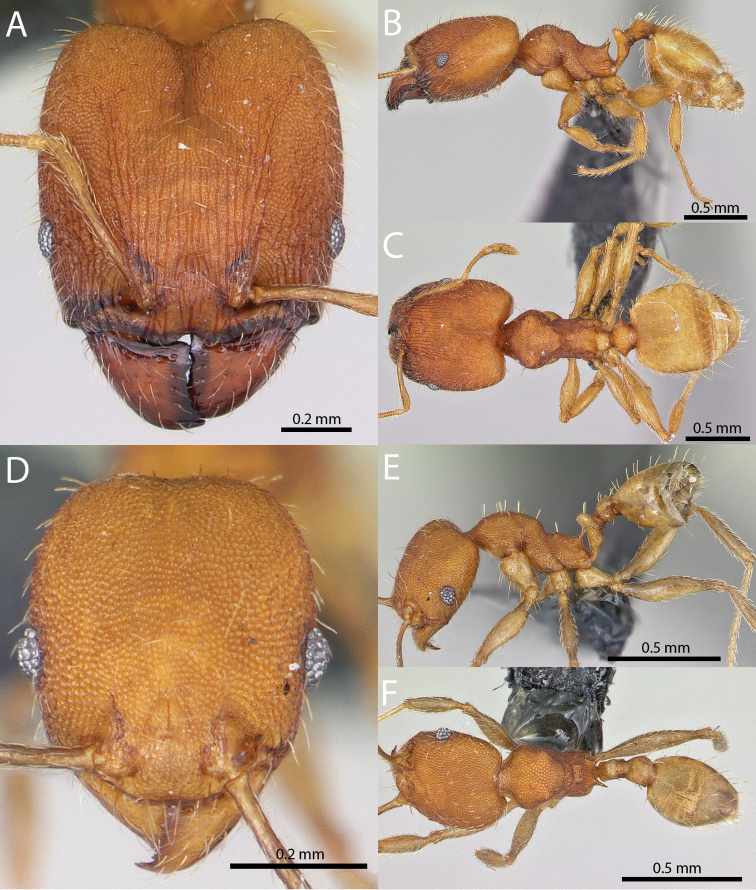
*Pheidole bilimeki* Mayr. Major worker, CASENT0173659: **A** full-face view **B** lateral view **C** dorsal view. Minor worker, CASENT0173658: **D** full-face view **E** profile view **F** dorsal view. From Antweb.org, photographs by April Nobile.

**Figure 76.**
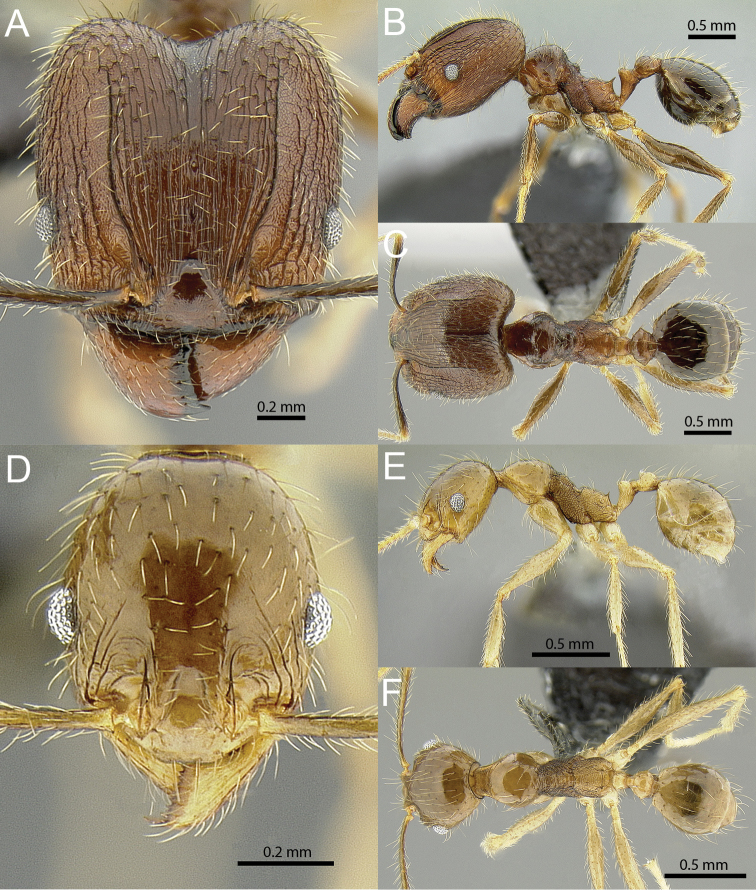
*Pheidole fervens* F. Smith. Major worker, CASENT0171099: **A** full-face view **B** lateral view **C** dorsal view. Minor worker, CASENT0171076: **D** full-face view **E** profile view **F** dorsal view. From Antweb.org, photographs by Eli Sarnat.

**Figure 77.**
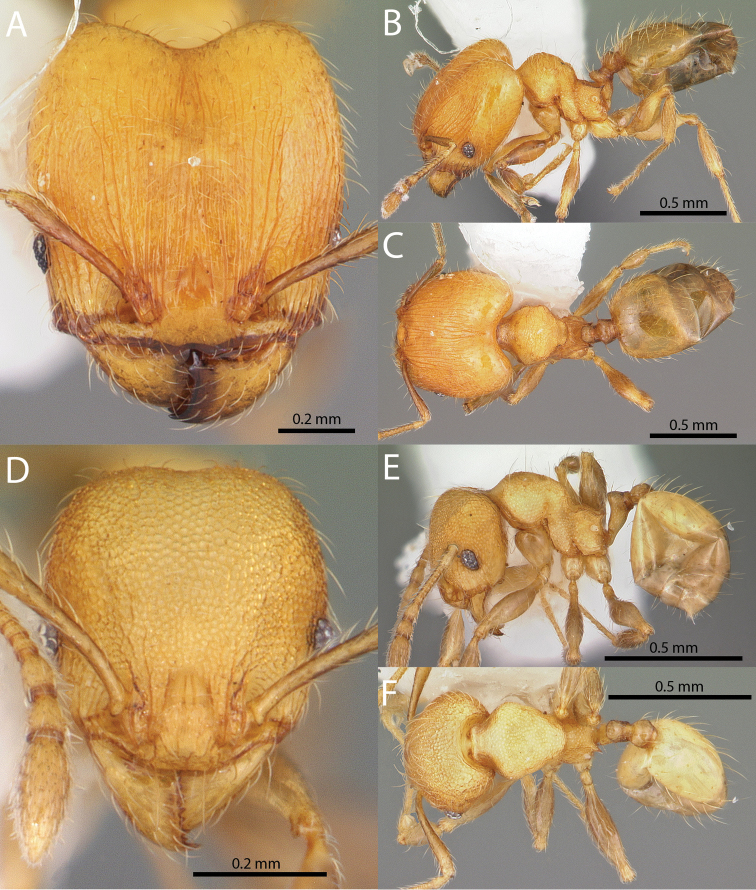
*Pheidole flavens* Roger. Major worker, CASENT0104398: **A** full-face view **B** lateral view **C** dorsal view. Minor worker, CASENT0104397: **D** full-face view **E** profile view **F** dorsal view. From Antweb.org, photographs by April Nobile.

**Figure 78.**
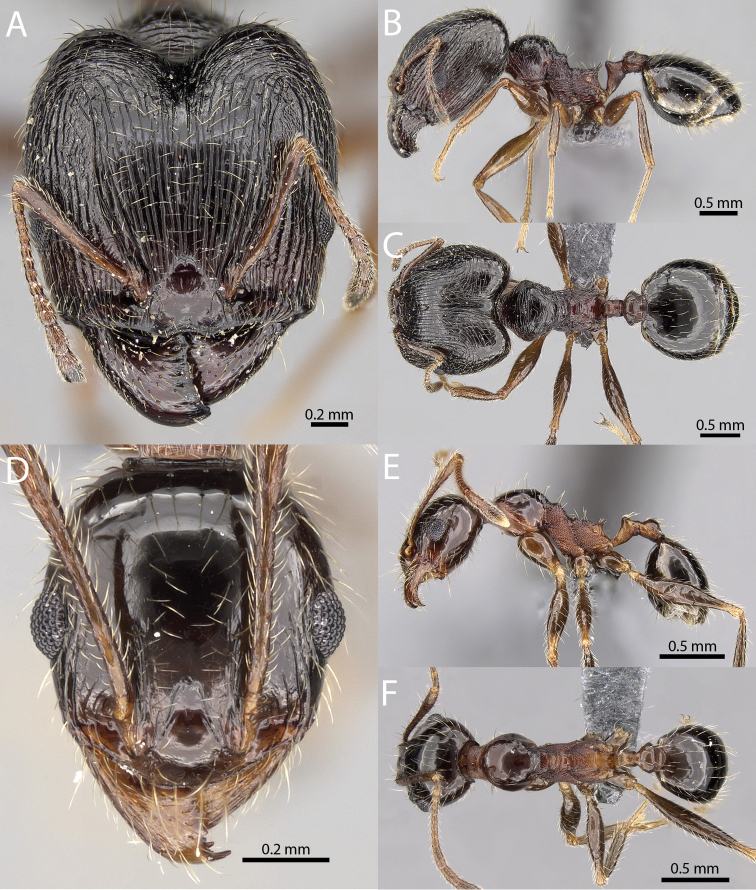
*Pheidole indica* Mayr. Major worker, CASENT0264427: **A** full-face view **B** lateral view **C** dorsal view. Minor worker, CASENT0263700: **D** full-face view **E** profile view **F** dorsal view. From Antweb.org, photographs by Estella Ortega.

**Figure 79.**
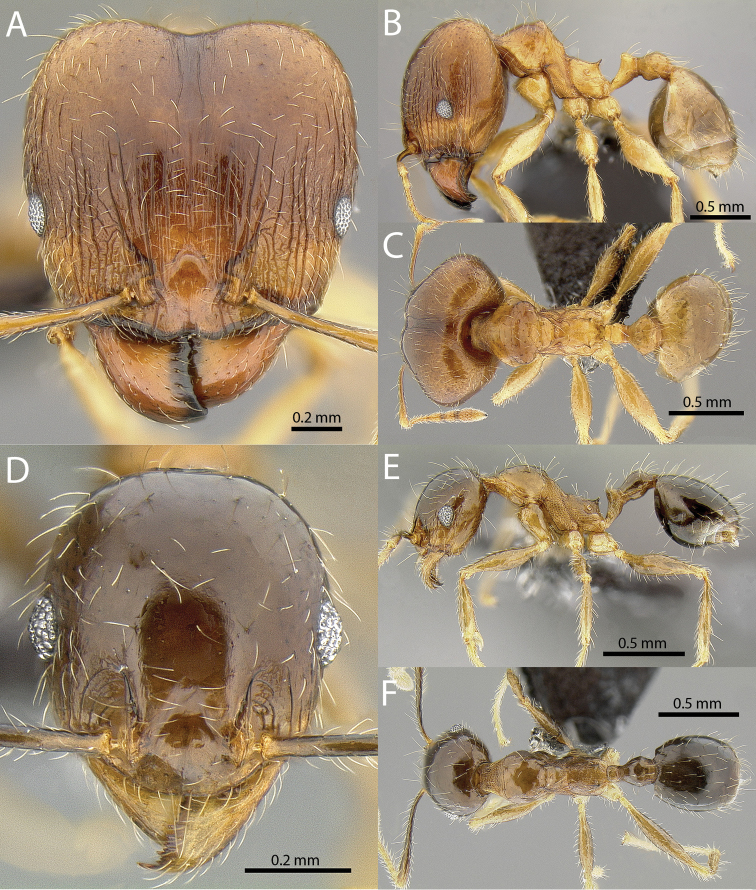
*Pheidole megacephala* (Fabricius). Major worker, CASENT0171036: **A** full-face view **B** lateral view **C** dorsal view. Minor worker, CASENT0171092: **D** full-face view **E** profile view **F** dorsal view. From Antweb.org, photographs by Eli Sarnat.

**Figure 80.**
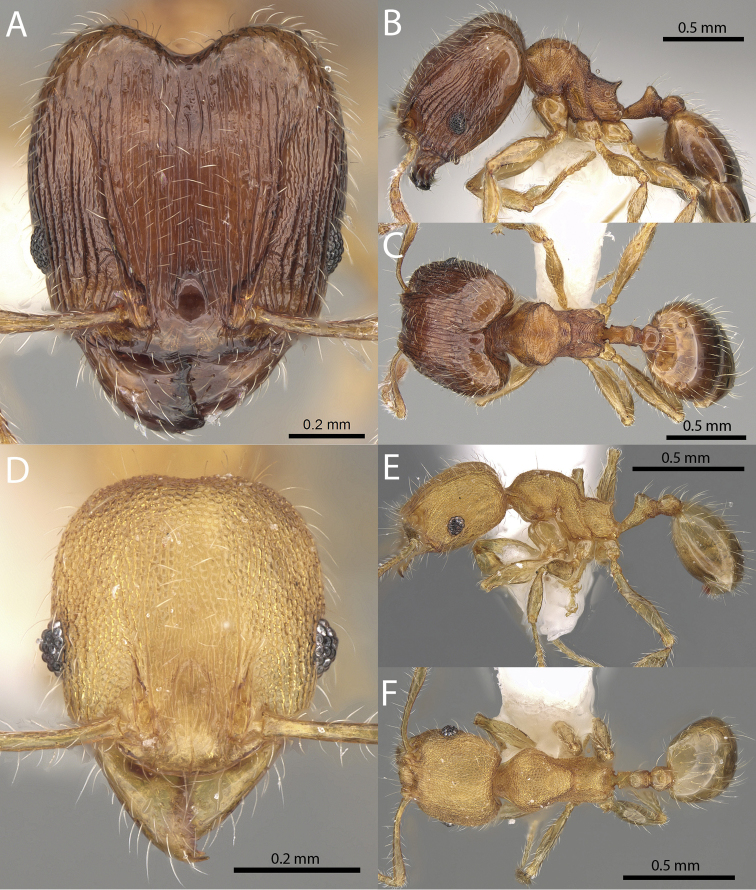
*Pheidole navigans* Forel. Major worker, BPBMENT2006029775: **A** full-face view **B** lateral view **C** dorsal view. Minor worker, BPBMENT2006029771: **D** full-face view **E** profile view **F** dorsal view. From Antweb.org, photographs by Eli Sarnat.

**Figure 81.**
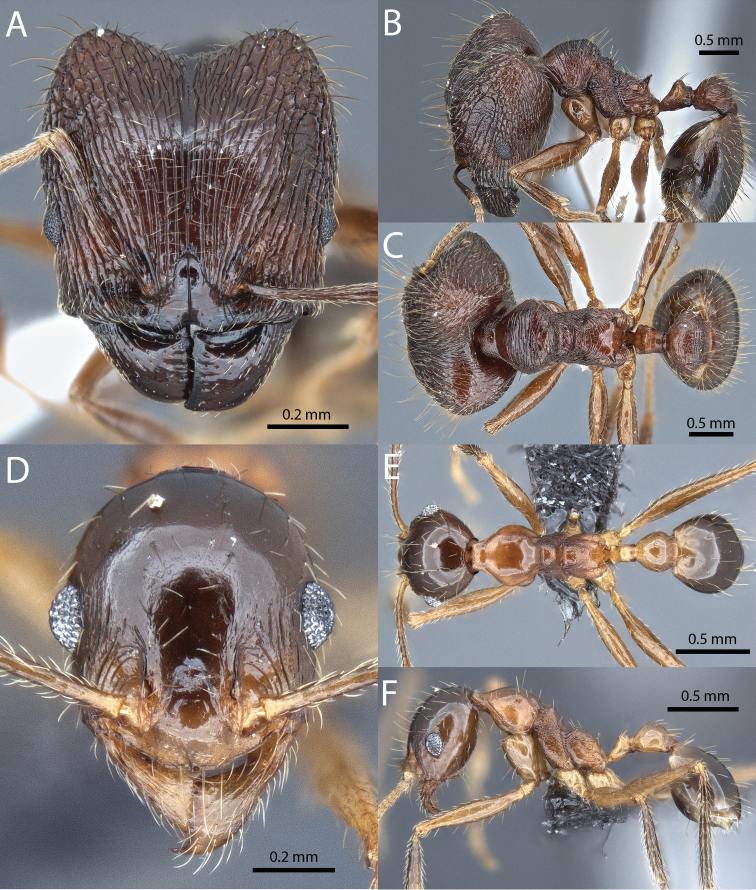
*Pheidole noda* F. Smith. Major worker, CASENT0282545: **A** full-face view **B** lateral view **C** dorsal view. Minor worker, CASENT0741212: **D** full-face view **E** profile view **F** dorsal view. From Antweb.org, photographs by Masako Ogasawara.

**Figure 82.**
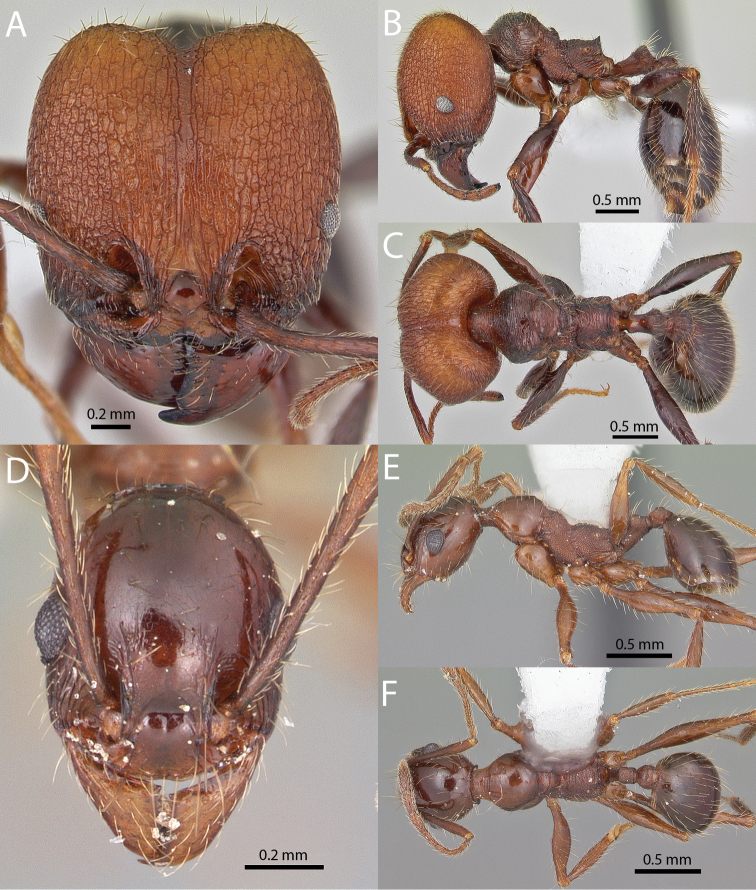
*Pheidole obscurithorax* Naves. Major worker, CASENT0178041: **A** full-face view **B** lateral view **C** dorsal view. Minor worker, CASENT0104420: **D** full-face view **E** profile view **F** dorsal view. From Antweb.org, photographs by April Nobile.

**Figure 83.**
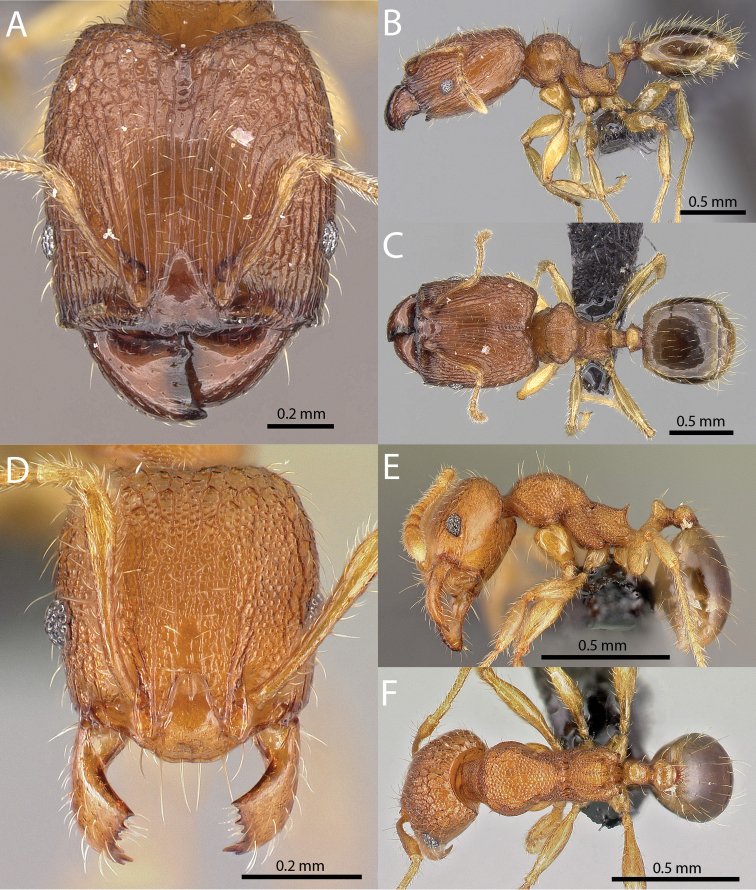
*Pheidole parva* Mayr. Major worker, CASENT0160280: **A** full-face view **B** lateral view **C** dorsal view. Minor worker, CASENT0160528: **D** full-face view **E** profile view **F** dorsal view. From Antweb.org, photographs by Estella Ortega.

**Figure 84.**
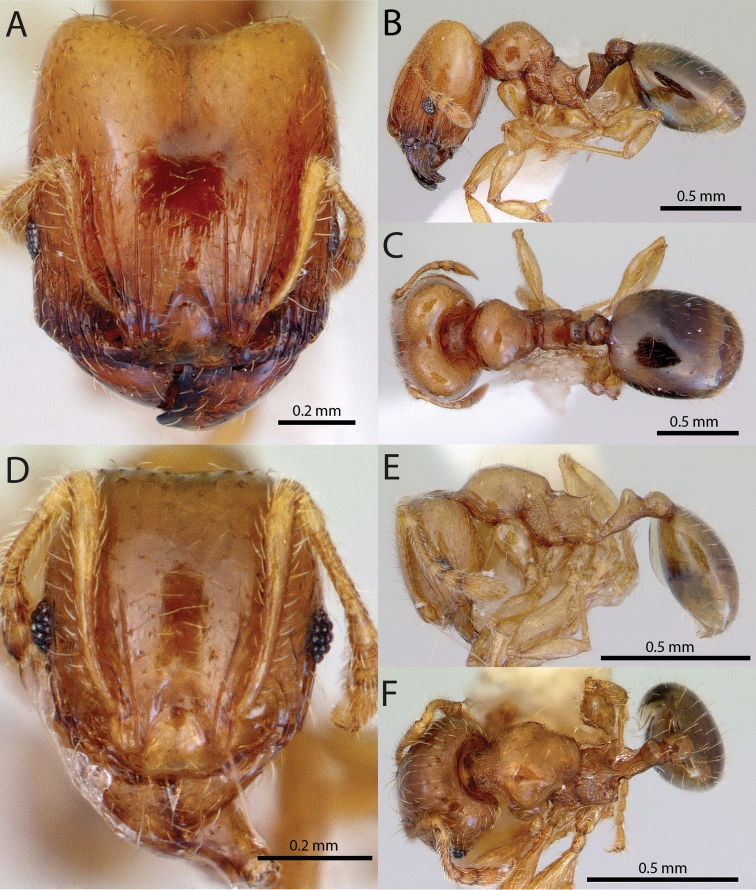
*Pheidole proxima* Mayr. Major worker, CASENT0172362: **A** full-face view **B** lateral view **C** dorsal view. Minor worker, CASENT0172363: **D** full-face view **E** profile view **F** dorsal view. From Antweb.org, photographs by April Nobile.

**Figure 85.**
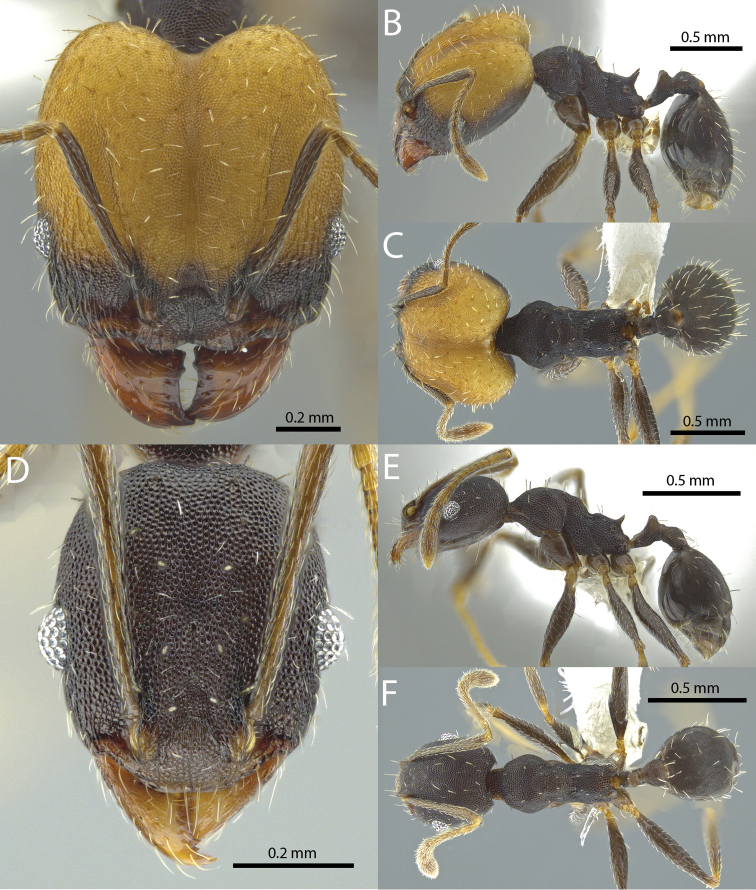
*Pheidole punctatissima* Mayr. Major worker, CASENT0619681: **A** full-face view **B** lateral view **C** dorsal view. Minor worker, CASENT0619442: **D** full-face view **E** profile view **F** dorsal view. From Antweb.org, photograph by Jeremy Pillow.

**Figure 86.**
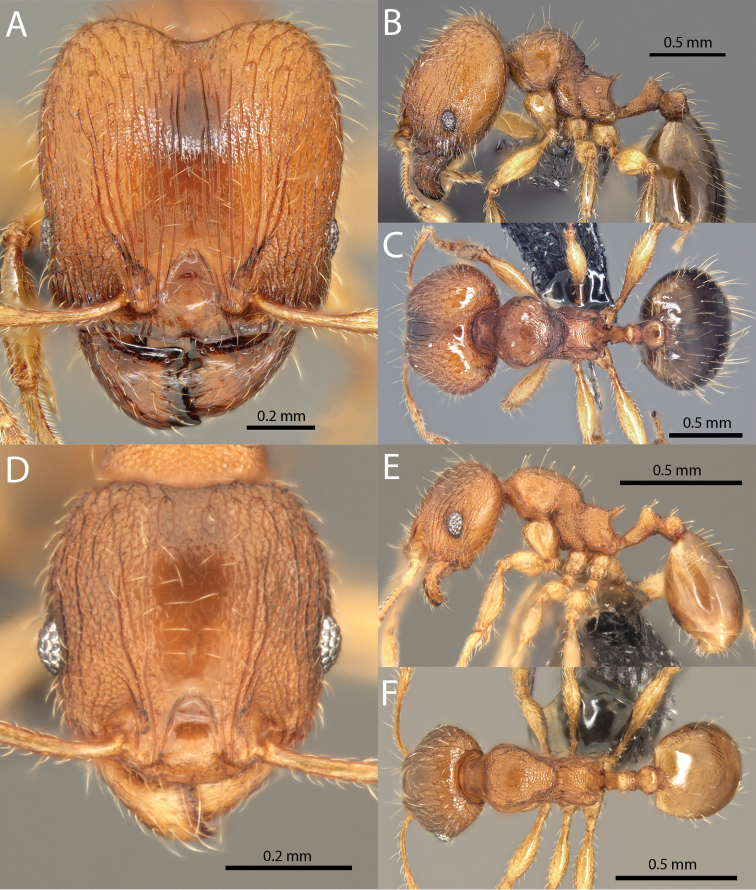
*Pheidole rugosula* Forel. Major worker, CASENT0717051: **A** full-face view **B** lateral view **C** dorsal view. Minor worker, CASENT0717052: **D** full-face view **E** profile view **F** dorsal view. From Antweb.org, photographs by Masako Ogasawara.

**Figure 87.**
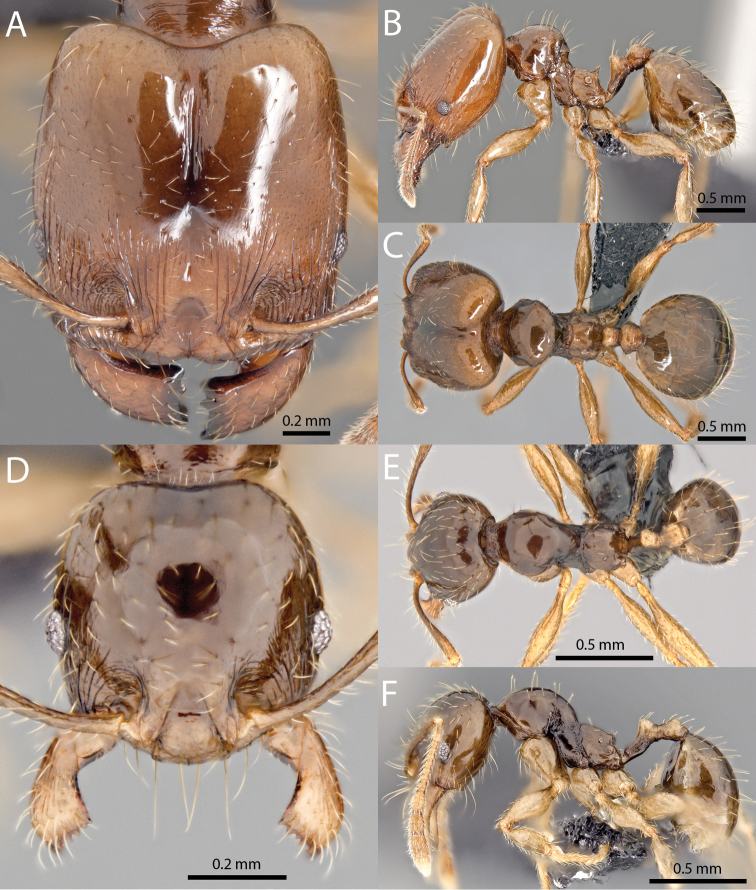
*Pheidole vigilans* F. Smith. Major worker, CASENT0717430: **A** full-face view **B** lateral view **C** dorsal view. Minor worker, CASENT0717429: **D** full-face view **E** profile view **F** dorsal view. From Antweb.org, photographs by Masako Ogasawara.

**Figure 88.**
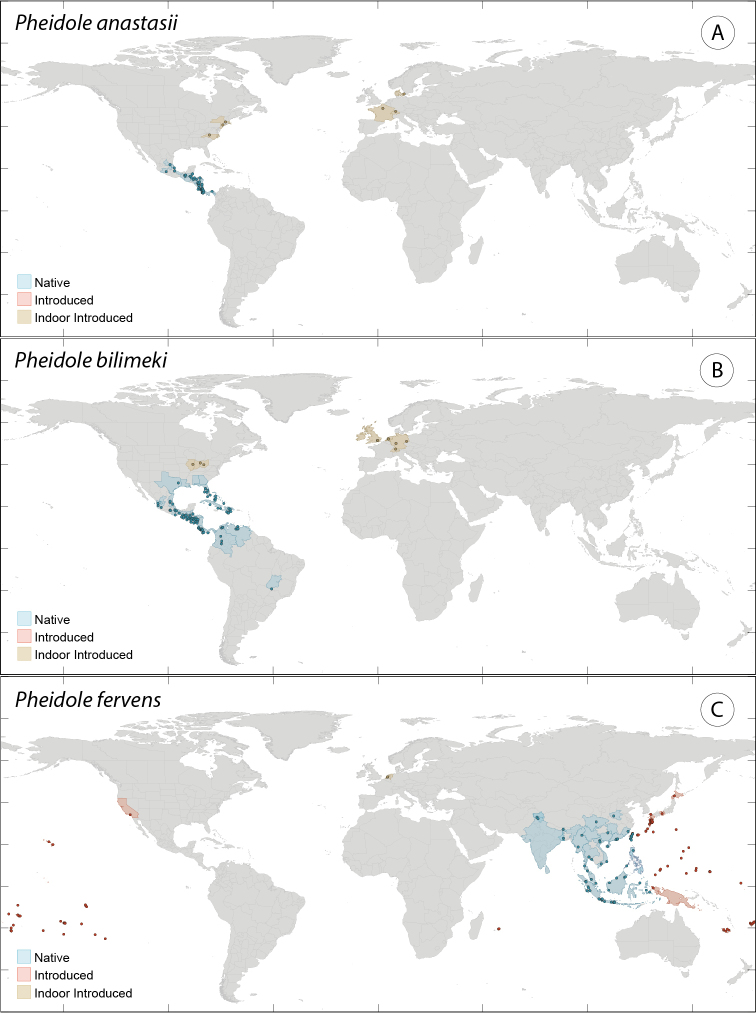

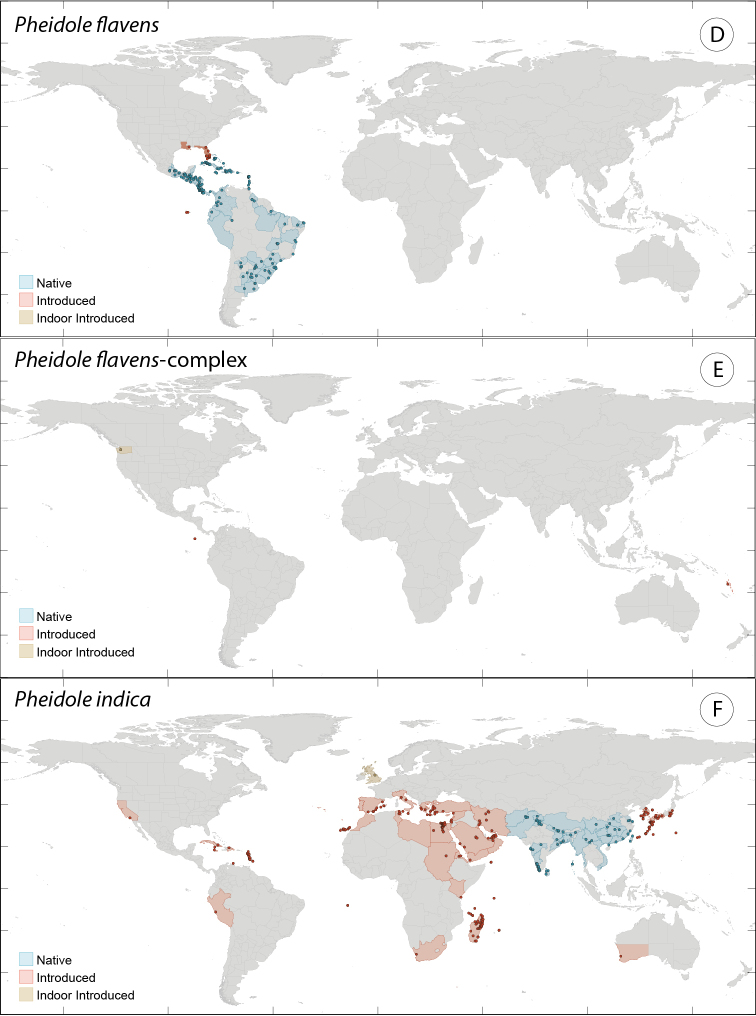

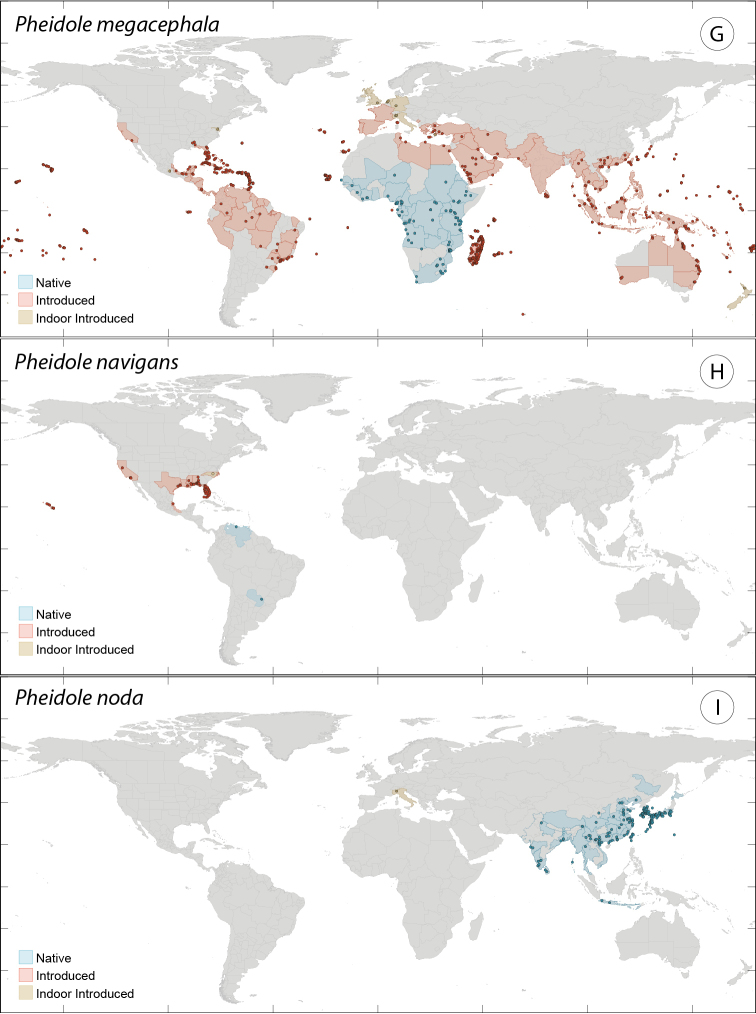

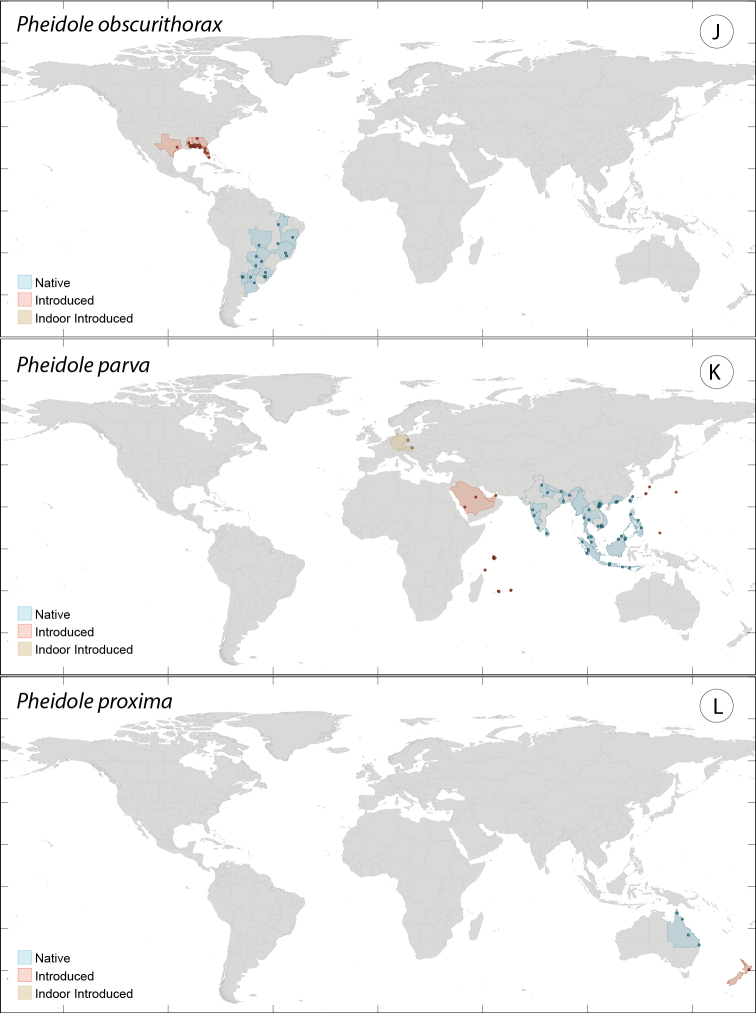

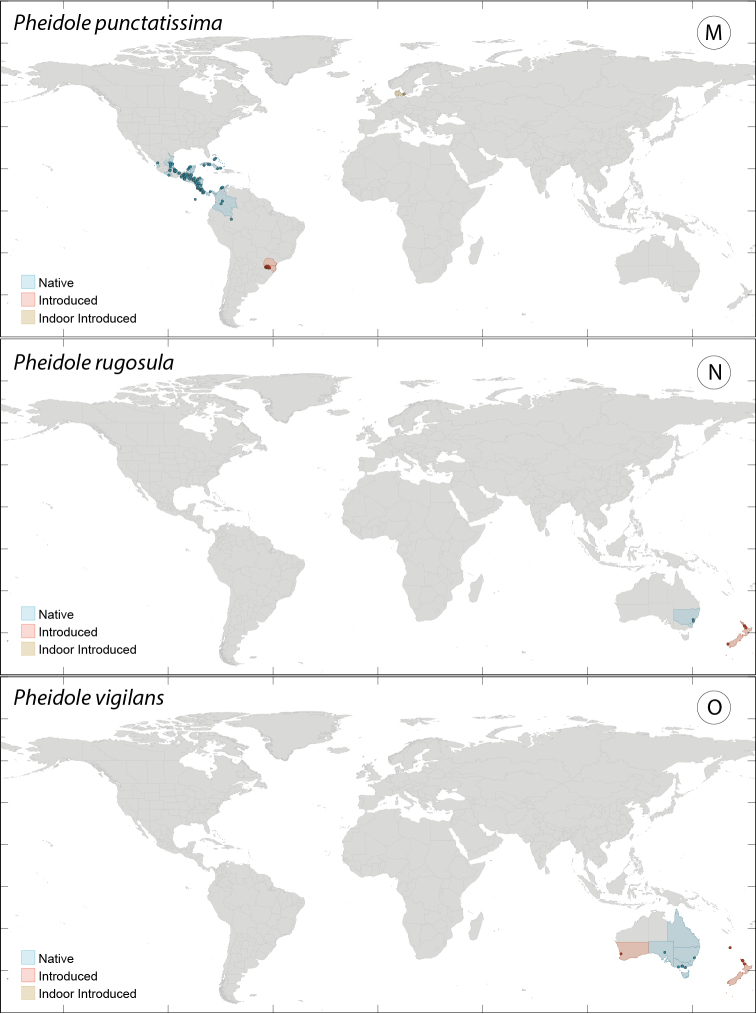
Geographic distribution of introduced *Pheidole* species. **A** *Pheidole anastasii* Emery **B** *Pheidole bilimeki* Mayr **C** *Pheidole fervens* F. Smith. Circle symbols represent georeferenced localities. Shaded polygons represent administrative units from which the respective species have been recorded as occurring. Larger countries are subdivided into states or provinces for increased geographic resolution. Blue = putative native occurrence records. Red = putative introduced occurrence records. Yellow = records for indoor occurrences (heated buildings, greenhouses, etc.) in regions where the species is incapable of year-round outdoor survival. Geographic distribution of introduced *Pheidole* species. **D** *Pheidole flavens* Roger **E** *Pheidole flavens*-complex (excluding determined records of *Pheidole flavens* Roger and *Pheidole navigans* Forel) **F** *Pheidole indica* Mayr. Circle symbols represent georeferenced localities. Shaded polygons represent administrative units from which the respective species have been recorded as occurring. Larger countries are subdivided into states or provinces for increased geographic resolution. Blue = putative native occurrence records. Red = putative introduced occurrence records. Yellow = records for indoor occurrences (heated buildings, greenhouses, etc.) in regions where the species is incapable of year-round outdoor survival. Geographic distribution of introduced *Pheidole* species. **G** *Pheidole megacephala* (Fabricius) **H** *Pheidole navigans* Forel **I** *Pheidole noda* F. Smith. Circle symbols represent georeferenced localities. Shaded polygons represent administrative units from which the respective species have been recorded as occurring. Larger countries are subdivided into states or provinces for increased geographic resolution. Blue = putative native occurrence records. Red = putative introduced occurrence records. Yellow = records for indoor occurrences (heated buildings, greenhouses, etc.) in regions where the species is incapable of year-round outdoor survival. Geographic distribution of introduced *Pheidole* species. **J** *Pheidole obscurithorax* Naves **K** *Pheidole parva* Mayr **L** *Pheidole proxima* Mayr. Circle symbols represent georeferenced localities. Shaded polygons represent administrative units from which the respective species have been recorded as occurring. Larger countries are subdivided into states or provinces for increased geographic resolution. Blue = putative native occurrence records. Red = putative introduced occurrence records. Yellow = records for indoor occurrences (heated buildings, greenhouses, etc.) in regions where the species is incapable of year-round outdoor survival. Geographic distribution of introduced *Pheidole* species. **M** *Pheidole punctatissima* Mayr **N** *Pheidole rugosula* Forel **O** *Pheidole vigilans* (F. Smith).Circle symbols represent georeferenced localities. Shaded polygons represent administrative units from which the respective species have been recorded as occurring. Larger countries are subdivided into states or provinces for increased geographic resolution. Blue = putative native occurrence records. Red = putative introduced occurrence records. Yellow = records for indoor occurrences (heated buildings, greenhouses, etc.) in regions where the species is incapable of year-round outdoor survival.

## Supplementary Material

XML Treatment for Pheidole anastasii

XML Treatment for Pheidole bilimeki

XML Treatment for Pheidole fervens

XML Treatment for Pheidole flavens

XML Treatment for Pheidole indica

XML Treatment for Pheidole megacephala

XML Treatment for Pheidole navigans

XML Treatment for Pheidole noda

XML Treatment for Pheidole obscurithorax

XML Treatment for Pheidole parva

XML Treatment for Pheidole proxima

XML Treatment for Pheidole punctatissima

XML Treatment for Pheidole rugosula

XML Treatment for Pheidole vigilans

## Supplementary materials

Supplementary material 1 Biogeographic data Data type: specimens data Explanation note: Occurrence data used for analyses and generation of distribution maps (Figure 88) sorted by GABI number. GABI number refers to unique code assigned to each individual record of the Global Ant Biodiversity Informatics. ‘Accession number’ refers to specific accession codes for each individual record within the database. If the record refers to specimen data, the unique specimen identifier code is included in the Accession number field. ‘Bentity2’ refers to the geographic unit used to map species distribution (Guénard et al., in prep.). The Precision field provides a rough estimate of the geocoded location accuracy. Manual: location was manually geocoded after correcting the locality (old names, changing borders, wrongly translated). Source: coordinates are taken from the original data (the published coordinates). All other values are derived through geocoding the locations with the Bing geocode API. These values give an estimate of the accuracy which can include anything from an exact address to a small island. The more precise this value describes a location the more likely it is for a location to be accurately geocoded. An ‘address’ is for example likely to be more accurate than a ‘bay’. This dataset is made available under the Open Database License (http://opendatacommons.org/licenses/odbl/1.0/). The Open Database License (ODbL) is a license agreement intended to allow users to freely share, modify, and use this Dataset while maintaining this same freedom for others, provided that the original source and author(s) are credited.Eli M. Sarnat, Georg Fischer, Benoit Guénard, Evan P. Economo
